# Blood-Based Biomarkers for Glioma in the Context of Gliomagenesis: A Systematic Review

**DOI:** 10.3389/fonc.2021.665235

**Published:** 2021-06-04

**Authors:** Hamza Ali, Romée Harting, Ralph de Vries, Meedie Ali, Thomas Wurdinger, Myron G. Best

**Affiliations:** ^1^ Department of Neurosurgery, Brain Tumor Center Amsterdam, Cancer Center Amsterdam, Amsterdam UMC, VU University Medical Center and Academic Medical Center, Amsterdam, Netherlands; ^2^ Medical Library, Vrije Universiteit, Amsterdam, Netherlands

**Keywords:** diagnostics, liquid biopsy, blood, glioblastoma, glioma

## Abstract

**Background:**

Gliomas are the most common and aggressive tumors of the central nervous system. A robust and widely used blood-based biomarker for glioma has not yet been identified. In recent years, a plethora of new research on blood-based biomarkers for glial tumors has been published. In this review, we question which molecules, including proteins, nucleic acids, circulating cells, and metabolomics, are most promising blood-based biomarkers for glioma diagnosis, prognosis, monitoring and other purposes, and align them to the seminal processes of cancer.

**Methods:**

The Pubmed and Embase databases were systematically searched. Biomarkers were categorized in the identified biomolecules and biosources. Biomarker characteristics were assessed using the area under the curve (AUC), accuracy, sensitivity and/or specificity values and the degree of statistical significance among the assessed clinical groups was reported.

**Results:**

7,919 references were identified: 3,596 in PubMed and 4,323 in Embase. Following screening of titles, abstracts and availability of full-text, 262 articles were included in the final systematic review. Panels of multiple biomarkers together consistently reached AUCs >0.8 and accuracies >80% for various purposes but especially for diagnostics. The accuracy of single biomarkers, consisting of only one measurement, was far more variable, but single microRNAs and proteins are generally more promising as compared to other biomarker types.

**Conclusion:**

Panels of microRNAs and proteins are most promising biomarkers, while single biomarkers such as GFAP, IL-10 and individual miRNAs also hold promise. It is possible that panels are more accurate once these are involved in different, complementary cancer-related molecular pathways, because not all pathways may be dysregulated in cancer patients. As biomarkers seem to be increasingly dysregulated in patients with short survival, higher tumor grades and more pathological tumor types, it can be hypothesized that more pathways are dysregulated as the degree of malignancy of the glial tumor increases. Despite, none of the biomarkers found in the literature search seem to be currently ready for clinical implementation, and most of the studies report only preliminary application of the identified biomarkers. Hence, large-scale validation of currently identified and potential novel biomarkers to show clinical utility is warranted.

## Introduction

**Figure 1:**
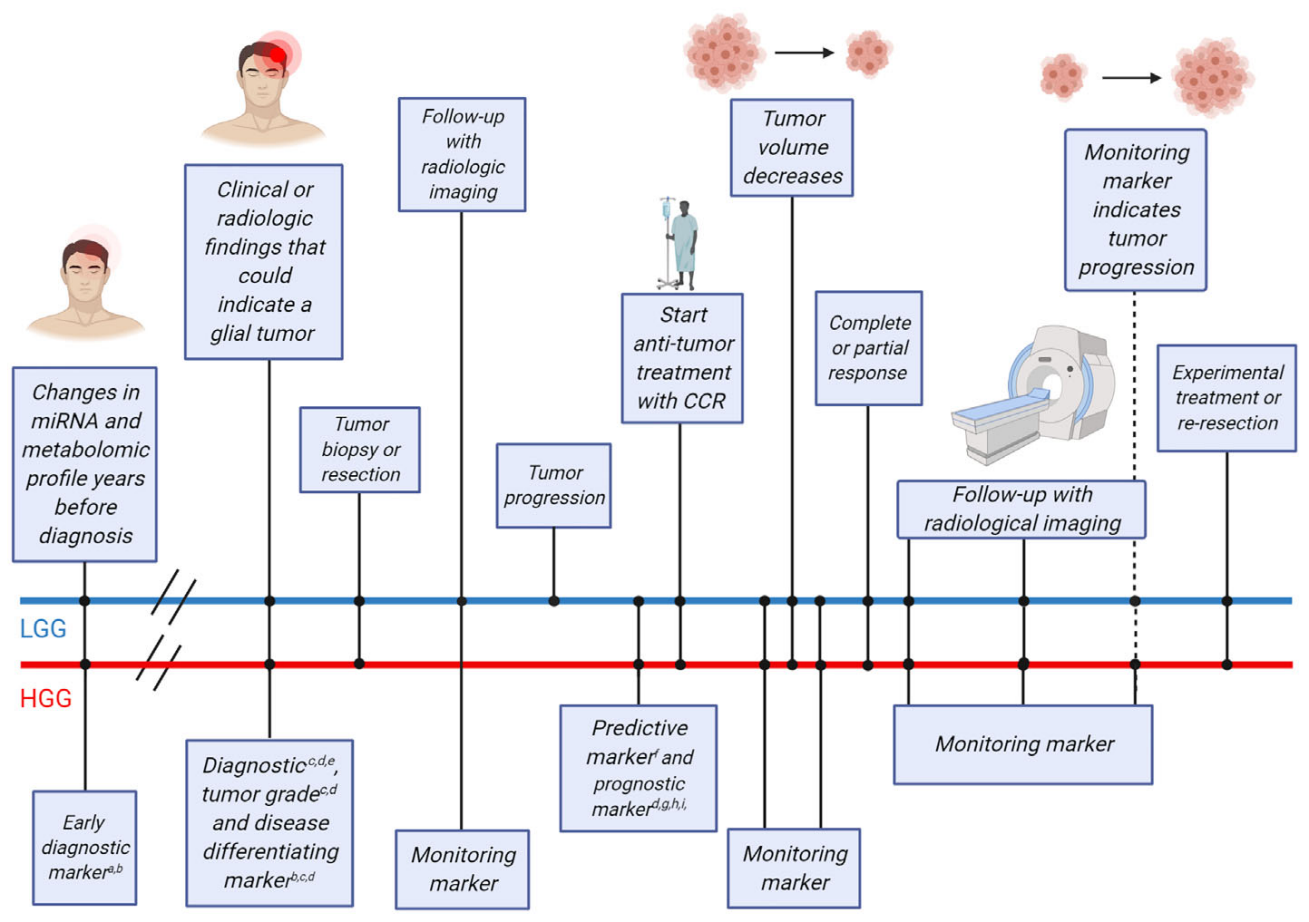
Timeline of clinical events for glioma patients and possible blood-based biomarkers that could be employed at different points in time. The straight lines indicate timelines for two example glioma patients [upper blue line for a lower-grade glioma (LGG) patient, lower red line for a high-grade glioma (HGG) patient]. Clinical events that occur on either timeline are indicated using dots and the clinical events are described in boxes connected to the dots. Early diagnostic markers have been found more than two decades before glioma diagnosis and could be used as a screening tool in the healthy population for patients older than 50 years. At the time of clinical or radiological findings that may indicate the growth of a glial tumor, diagnostic, tumor grade and disease differentiating biomarkers may be used to supplement the diagnostic procedure. Following, surgery (tumor tissue biopsy and/or tumor resection) may be performed, including either tumor resection or only a tumor tissue biopsy for definite histopathological diagnosis. At this point, the brain tumor is identified as a HGG or LGG. Following discussion of the case in a multidisciplinary tumor board, treatment may be initiated in patients with more malignant tumor types, while patients with less malignant tumor types may be subjected to frequent follow-up using monitoring markers and radiological imaging to monitor potential tumor progression. At the moment of tumor progression in patients with less malignant tumors or directly after surgical resection in patients with malignant tumors, predictive markers may provide additional information on the potential benefit of adjuvant treatment. Anti-tumor treatment with conventional chemo- and/or radiotherapy (CCR) is currently usually initiated at this point. Monitoring blood markers can detect tumor volume decrease over time. Patients with complete or partial response can be followed using radiological imaging and monitoring markers to distinguish between tumor progression or pseudoprogression. Patients with stable disease, progressive disease or tumor progression after complete or partial response may be admitted for experimental treatments. For each biomarker purpose, several potential blood-based biomarkers are listed *^a^Tocopherols; ^b^miR-21; ^c^GFAP; ^d^Panels of miRNAs, proteins and metabolites; ^e^IL-10; ^f^NLR; ^g^YKL-40; ^h^F-NLR; ^i^F-NLR-AGR.* Figure was adapted from “Cell Transfer Protocol”, by BioRender.com (2021). Retrieved from: https://app.biorender.com/biorender-templates.

**Figure 2:**
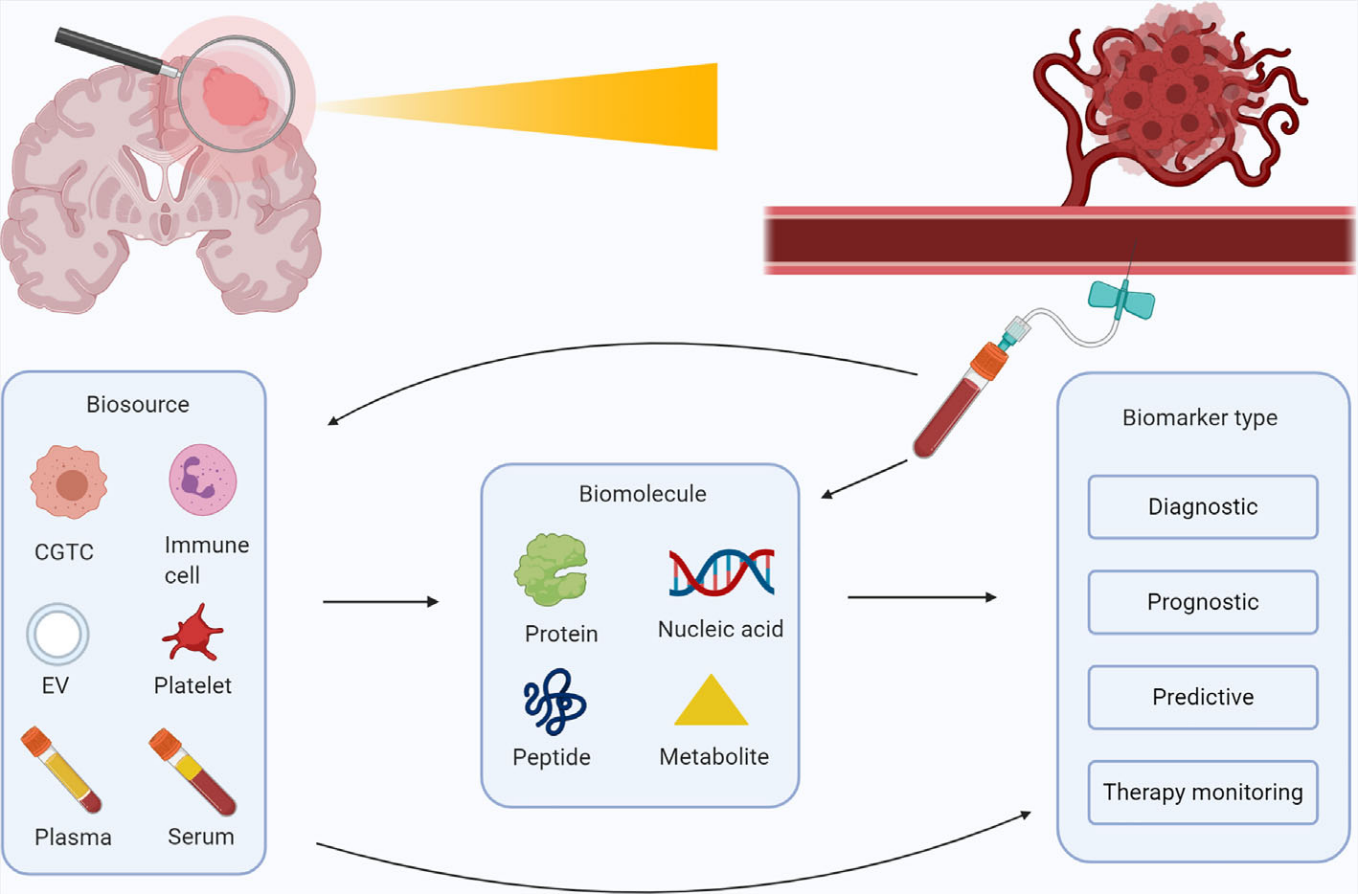
Overview of possible blood-based biomarkers for glioma and their purposes. Schematic overview of the several biosources (plasma, serum, extracellular vesicles, blood platelets, circulating immune cells, and circulating glioma tumor cells) and biomolecules (proteins, nucleic acids, metabolomics and peptides) that are identified for patients with glioma. These biomolecules can be collected in a vial of blood, and employed as a diagnostic, prognostic, predictive, or therapy monitoring marker. Figure was created with BioRender.com.

Gliomas, and especially glioblastomas, are one of the most devastating primary tumors of the central nervous system with a dismal prognosis. Definite diagnosis of the disease is particularly dependent on tumor tissue assessment, though repetitive collection of tumor tissue to track tumor molecular evolution and/or tumor progression and regression is not desired. Part of such follow-up monitoring can be done *via* (advanced) imaging techniques. Also, the past years a plethora of research has been published in which blood-based biomarkers for glioma were utilized with various purposes. This is in line with the upcoming field of so-called ‘liquid biopsies’ in other (solid) tumor types. Blood-based biomarkers were found to be helpful as (early) diagnostic markers, including tumor grade and brain disease differentiating markers, prognostic, predictive, and monitoring markers (1–3) in glioma patients. Early diagnostic blood markers are biomarkers that can be utilized to predict development of glioma in individuals years before clinical or radiological signs can be noticed. These markers may be useful to screen patients with familial disorders such as neurofibromatosis type I, Li–Fraumeni syndrome and others that are at risk of development of a glioma (4). The term ‘diagnostic marker’ in this systematic review implies markers that were used to differentiate between healthy individuals and glioma patients. The terms ‘tumor grade and brain disease/tumor type-differentiating markers’ are employed to further classify glial tumors in glioma patients. Predictive markers can be employed to predict response to therapy and thus aid in correct therapy selection by examining the expression of histopathological features present in the glial tumor. Lastly, monitoring markers can be used to monitor tumor volume or monitor tumor progression as opposed to pseudoprogression after treatment. Tumor volume monitoring biomarkers that are stated in this review, were mainly used to predict tumor volume pre-treatment, but may also have use as volume monitoring markers after treatment. An example of how the different biomarker types may be employed during the clinical course of a typical glioma patient is detailed in **Figure 1**. Here, the timing of different biomarker types during and before treatment of future glioma patients is illustrated, along a timeline of clinical events in high- and low-grade glioma patients. Currently, it remains unclear which biomarkers or which combination of biomarkers will have most clinical utility. The aim of this systematic review is to identify and highlight the most promising and well-researched blood-based biomarkers for patients with glioma. Identification of a novel biomarker should start with the desired clinical groups to separate in mind. Distinguishing these groups should have clinical relevance, e.g. monitoring progression of lower-grade glioma patients to a secondary glioblastoma thereby tailoring treatment and providing prognostic information, or identification of patients with glioblastoma on treatment that develop tumor pseudo-progression as opposed to true-progression, thereby optimizing treatment schedules. With this, we believe that a promising biomarker should meet several criteria. First, the accuracy of the biomarker should be sufficiently high, measure exactly the difference between the clinically relevant groups without contribution from confounding variables, and adjusted towards its clinical context. For example, a diagnostic biomarker should be very precise, whereas predictive biomarkers should be very specific In order to not withhold patients potential therapeutic options. Second, a biomarker should be resistant to inter- and intra-individual factors, such as diurnal variation, body temperature, comorbidities, medication, radiation therapy, exercise, fasting, sex, and race. Following, the analytical devices that are used to measure the biomarker should be relatively cheap, easy to operate, sensitive in determining low concentrations of biomarker and specific for the biomarker, avoiding false-positive test results. Lastly, the biomarker should have been tested in several (preferably independent) studies with large patient populations, which include independent validation cohorts. Here, we provide a useful and easily accessible overview of the studies performed so far, after which we discuss the most promising markers that may deserve further validation. The review has been subdivided into several biosources and biomolecules as illustrated in **Figure 2**, and will close with a discussion of this dynamic field.

## Methods

### Search Strategy and Study Selection

**Figure 3:**
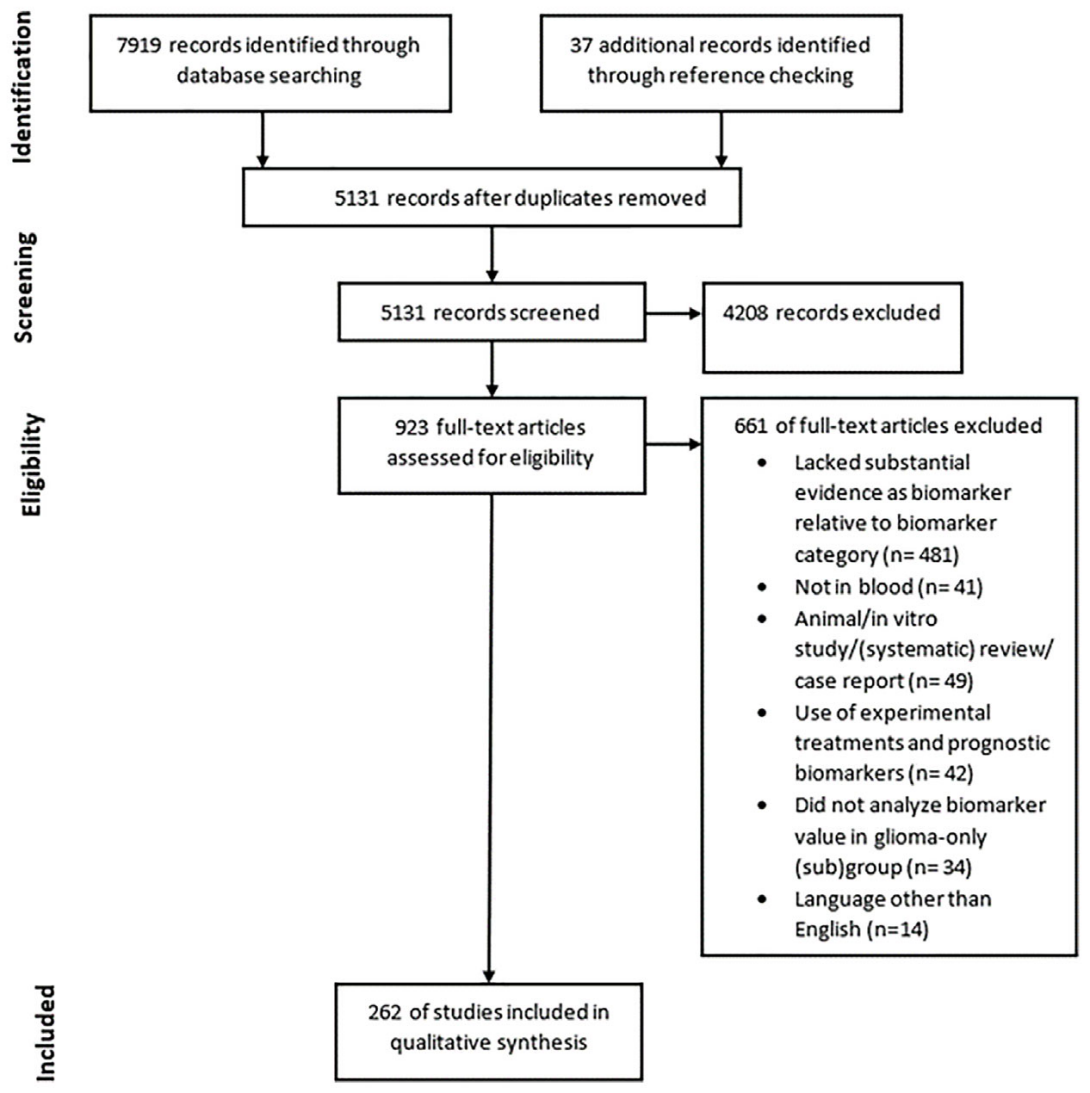
PRISMA diagram showing the amount of records found through database searching and reference checking, the amount of records screened and removed using exclusion criteria and the amount of records included in the final qualitative synthesis.

We conducted systematic searches in the bibliographic databases PubMed and Embase from inception up to August 7, 2020. The following terms, including synonyms and closely related words, as index terms or free-text words were used: “Glioma”, “Blood”, “Biomarkers”. These were combined with possible purposes of biomarkers such as prognosis, diagnosis, monitoring and other related terms. Duplicate articles were excluded. The references of the identified articles were searched for relevant publications. The full search strategies for PubMed and Embase can be found in **Supplemental Tables 1** and **2**. Three authors independently screened all potentially relevant titles and abstracts for eligibility. If necessary, the full-text article was reassessed for the eligibility criteria. Differences in judgement were resolved through a consensus procedure. Studies were included if they met all of the following criteria: i) Histologically proven glial tumors; ii) Measured biomarker concentrations in whole blood, serum or plasma; iii) Correlation of the biomarkers with at least one of the following: glial tumor diagnosis, glial tumor grade, (glial) tumor type such as astrocytoma or oligodendroglioma, overall survival of patients, glial tumor manifestation prior to diagnosis, and tumor burden; iv) Included measures, such as Area Under the Curve (AUC), accuracy, hazard ratio (HR), sensitivity, specificity values and/or the degree of significance using a p-value. We excluded studies if they i) Reported on biomarkers found in CSF, tumor tissue or other non-hematogenous fluids such as cyst fluid; ii) Were of the following publication types: editorials, letters, interviews, case reports, animal studies, in vitro studies, pediatric studies, or (systematic) reviews; iii) Did not analyze biomarker value in a glioma-only (sub)group; iv) Reported on prognostic biomarkers when patients with glial tumors were treated with experimental treatments; v) Were published in languages other than English; vi) Described biomarker(s) which lacked substantial evidence relative to the biomarker categories. Substantial evidence is quantified as able to differentiate between clinically relevant groups in at least four independent studies. The process of retrieving all articles relevant to our systematic review is summarized in a Preferred Reporting Items for Systematic Reviews and Meta-Analyses (PRISMA) flowchart (see **Figure 3**).

### Data Extraction and Study Quality Assessment

Details per study (e.g. biosource and biomolecule), study population type (e.g. glioma or glioblastoma patients) and marker clinical group separating ability quantified as AUC, sensitivity, specificity, accuracy or hazard ratio can be found in the **Supplementary Materials**. In the **Supplementary Tables**, biomarkers are separated by purpose as diagnostic (**Supplemental Tables 3**–**5**), prognostic (**Supplemental Table 6**), predictive (**Supplemental Table 7**), and therapy monitoring (**Supplemental Tables 8** and **9**) markers. A separate table with panels of biomarkers and their potential function has been added as well (**Supplemental Table 10**). The summarized methodologies and results of included studies were used to critically assess the quality of the included studies. The evaluation of study quality is discussed in the results section.

## Results

### Study Selection and Characteristics of Selected Studies

The literature search generated a total of 7,919 references of which 3,596 were identified in PubMed and 4,323 in Embase, of which 262 studies were eligible for inclusion (**Figure 3**). A plethora of biomarkers were identified that could differentiate between clinically relevant patient groups. However, most markers were only found to be dysregulated in one group as compared to the other in only one or two studies. Therefore, we describe in this systematic review only markers that could differentiate between clinically relevant groups with significant results in at least four independent studies. We regarded these as the most promising biomarkers. Many studies did not include large patient or control populations of >100 patients or any validation cohort at all. Also, studies often did not report biomarker accuracies. The markers were divided into four relevant biomolecule groups: proteins, nucleic acids, circulating cells and metabolomics, and the most promising markers within these categories are discussed below. Due to word restrictions, we decided to report on glioma patients in general, and in most cases not per histopathological subtype separately, though we do understand that such separation is of clinical importance. The histopathological classification of gliomas is continuously developing with implementation of multiple (novel) molecular tissue markers (4). Hence, in retrospect it is not always possible to correlate the patients’ diagnoses as provided in the identified studies to the current standards. We decided to report the diagnosis as provided in the referenced studies.

## Proteins and Peptides

### Interleukins

Interleukins (ILs) are a group of cytokine proteins usually secreted by inflammatory cells by means of inter-inflammatory cell communication. Interleukins can promote or inhibit carcinogenesis. It is possible that glial tumors create a protumor environment by actively secreting (5, 6) and/or recruiting brain-resident cells such as microglia to stimulate the secretion of cytokines with pro-tumorigenic functions (7). Interleukins such as IL-1b (8–12), IL-6 (8–10, 13–17) and IL-10 (8, 10, 13, 18–23) have been found to be increased in glioma patients compared to healthy individuals. Accuracies of AUC = 0.9-1.0 (13) and a sensitivity 95% and specificity of 85% (19), have been found. However, IL-1b (21, 24) and IL-6 (21, 22, 25, 26) concentrations were also found to not be changed compared to controls or even decreased in glioma patients compared to controls. IL-1b (12) and IL-6 (17) levels may also be increased in patients with higher glioma grades, however other studies could not find a significant difference in IL-6 concentrations between patients with higher and lower glioma grades (25–27). Furthermore, IL-6 (17, 28) and IL-10 (21) have been found to be correlated with worse survival, but other studies could not confirm this for IL-6 (14, 25, 29–31). Thus, interleukins may be potential biomarkers, especially for glioma diagnosis.

### S100 Protein Superfamily

Several S100-family members have been reported to contribute *in vivo* to tumor growth, metastasis, angiogenesis and immune invasion (32). Proteins from the S100 protein family including S100A8, S100A9 and S100B have been found to be increased in the blood of glioma patients compared to healthy individuals in multiple studies (33–39). However, it has also been reported that S100B is not changed in glioma patients compared to controls (40). Furthermore, it is unclear whether proteins of the S100-family are correlated with tumor grade (38, 40), tumor volume (39, 40) and survival (34, 41, 42). The accuracy of the inflammatory biomarker S100A8 is promising with a diagnostic AUC of 0.9 in glioblastoma patients (34). Glioblastoma and anaplastic astrocytoma patients could be differentiated with an AUC of 0.7 (34).

### TNF Protein Superfamily

Tumor necrosis factor (TNF) was reported to be a major mediator of cancer-related inflammation and is elevated in cancer patients with poor prognosis (43). In vitro studies with glioma cells have shown that TNF can stimulate angiogenesis, downregulate the tumor suppressor gene PTEN and increase glioma cell invasiveness (44). Currently it is unclear whether TNF-alfa and TNF-beta are increased (9, 10, 14, 45), decreased (16, 20) or not changed in the blood of glioma patients (8, 13, 31).

### Acute-Phase Reactant Proteins and Other Inflammatory Protein Markers

Acute-phase (reactant) proteins (APRPs) are proteins that become increased (positive APRPs) or decreased (negative APRPs) in serum or plasma by at least 25% in response to an inflammatory stimulus (46). As gliomas and other cancers are characterized by chronic inflammation, it is possible that APRPs are altered in patients with cancer and can be employed as biomarkers. Indeed, in many other cancer types positive APRPs such as α1-antitrypsin and ceruloplasmin have been found to be increased, while negative APRPs such as kininogen and α2-HS glycoprotein are found to be decreased (47). Similarly, in glioma patients many positive APRPs such as haptoglobin (48–51) or CRP (14, 48, 52, 53) were increased compared to healthy individuals with AUCs around 0.8 (50, 52, 53) (see **Supplemental Table 3**). However, it is not clear whether negative APRPs and markers of reduced inflammation such as albumin, the prognostic nutritional index (PNI) and the albumin-globulin-ratio are decreased or remain unchanged (54, 55). Glioma tumor grade may be correlated with an increase of positive APRPs and a decrease of negative APRPs. The positive APRP fibrinogen was increased in patients with higher tumor grades (56–58) and similar results were also found for the related inflammatory marker F-NLR-AGR (57). The negative APRP marker albumin was decreased (56, 58) in patients with higher tumor grades, as is for the serum markers Albumin-Globulin-Ratio (AGR) and PNI (54–56, 58, 59), but these significant results were refuted in other studies (55, 57). The grade discriminative AUCs of both positive and negative APRPs were between 0.6 and 0.7 (54, 56, 58). 

Several positive APRPs have been found to be dysregulated in patients with glioma with longer compared to shorter survival. The inflammatory marker CRP has been found to be decreased in the serum of patients with longer survival (52, 60, 61), but the prognostic value was low [(HR)=1.0] (52). However, significant results could not be confirmed elsewhere despite large patients series and multivariate analyses (14, 28, 62, 63). Furthermore, fibrinogen (56, 57, 64), fibrinogen-NLR score (64), F-NLR-AGR (57) and fibrinogen-albumin score (65) were all increased in glioma patients with worse survival. HRs were between 1.5-3.8 for fibrinogen and its related markers. Negative APRPs and markers of reduced inflammation such as albumin (56, 66–68), AGR (57, 69, 70), PNI (59, 69, 71), and the Sanbo Scoring System (72), were elevated in patients with prolonged survival compared to patients with shorter survival. However, other studies could not find a significant relationship between albumin (70, 71, 73, 74), AGR (75), PNI (74, 76) and survival. Lastly, APRPs may also have use as a marker to differentiate between glioma patients and patients with other intracranial diseases and as markers to detect IDH1-mutation and MGMT-methylation status. However, research on these topics is scarce at this moment. In all, it seems that positive APRPs and markers of increased inflammation are increased while negative APRPs and markers of reduced inflammation are decreased in patients with glial tumors and in particular patients with more malignant glial tumors (see **Supplemental Tables 3**–**8**).

### GFAP

Glial fibrillary acidic protein (GFAP) is a protein that is mainly expressed by astrocytes and aids in the maintenance of astrocytic structure and stability. Blood levels of GFAP can be increased after injury of the brain through strokes (77), traumatic brain injuries (78), and after brain surgery, including glioma resection (79–81). The blood levels are typically increased in the context of destruction of glial cells and opening of the blood-brain-barrier. As both usually do not occur in non-acute brain diseases such as multiple sclerosis or brain metastases, GFAP may be a specific marker for gliomas. Indeed, GFAP values (38, 80, 82–85) were found to be elevated in glioblastoma patients, but diagnostic sensitivities were rather variable: between 33% and 86% of glioblastoma had elevated GFAP concentrations (38, 82–86). GFAP diagnostic specificities were more uniform and ranged between 85-100% (80, 82, 84). However, GFAP concentrations were not elevated in the circulation of glioma patients with tumor grades lower than grade IV (80, 82, 85). Furthermore, GFAP was increased in patients with glioblastoma as compared to patients with lower tumor grades (38, 80, 82–85, 87) and in glioblastoma patients compared to patients with other brain pathologies such as brain metastases, meningioma or pituitary adenoma (38, 81, 82, 84–86, 88). Also, GFAP levels were increased in patients with worse survival (80, 84), greater tumor volume (40, 80, 82, 86, 88), higher Ki67 proliferation index and lack of IDH1-mutation (80). However, it was not always confirmed that circulating GFAP is correlated to tumor volume and survival (83). Thus, GFAP is a promising marker and might have value as biomarker for glioblastoma diagnosis, grade and tumor type differentiation.

### YKL-40

YKL-40 is a glycoprotein that is secreted by macrophages, chondrocytes and several cancer cell types (89), including glioma cells (90). The exact functions of YKL-40 in cancer are unknown, however, it may stimulate angiogenesis, cell proliferation, prevent cell apoptosis (91), and aid in tissue remodeling during inflammation (89). YKL-40 is found to be increased in cancer and in inflammatory diseases such as Crohn’s disease, COPD, ulcerative colitis and others (89). YKL-40 was found to be increased in glioma patients as compared to healthy individuals (86, 92–95). The AUC in one study was 0.9 (93). Furthermore, YKL-40 is increased in patients with high-grade glioma compared to patients with low-grade glioma (93, 94, 96). Also, high baseline YKL-40 and increases in YKL-40 during treatment were correlated with worse survival in glioma patients with hazard ratios between 1-2.2 (25, 95–98). However, it was also found that YKL-40 was not correlated with survival (99) and tumor volume (94, 96, 97). In all, YKL-40 is an interesting marker, especially for predicting patient survival.

### VEGF

The vascular endothelial growth factor (VEGF) is one of the growth factors that aids in glioma neovascularization and a well-studied biomarker in glioma patients. VEGF has been researched extensively and has been found to be increased in glioma patients (10, 12, 14, 16, 100–107). However, multiple other studies did not find a significant difference (22, 24, 108–111). The same controversial results were also found in other studies when VEGF was used as a blood biomarker for other purposes such as tumor grade differentiating marker (12, 102, 103, 112, 113), tumor type differentiating marker for patients with glioblastoma and patients with intracranial metastases (103, 104, 114) and prognostic marker in patients that received several types of therapies (14, 19, 29, 31, 112, 115). It remains unclear why such differences have been found. It is possible that patient populations were too small to find a significant effect as both in studies with and without a significant effect of VEGF, most of the studies included small patient populations (<100 patients). Hence, VEGF does not seem to be a promising blood biomarker at this moment.

### Coagulation in Glioma

It is well known that cancer causes hypercoagulability that can result in venous thromboembolisms (VTE), disseminated intravascular coagulation and other coagulation disorders. The relation between brain cancer and thrombo-embolic events seems to be especially strong, as brain tumor patients had the second highest rate of thrombo-embolic events from malignancies in 18 organs (116). It is possible that this hypercoagulable state can be retraced in the blood of glioma patients if procoagulant factors are increased while anti-coagulant factors are decreased. It was seen that a multitude of coagulation markers and procoagulant factors were significantly increased in the circulation of glioma patients compared to healthy individuals such as prothrombin factor 1 + 2 (14), tissue factor (117), coagulation factor VII (19) and P-selectin (118). Procoagulant markers correlated with tumor grade and worse prognosis (see **Supplemental Tables 4** and **6**). Especially fibrinogen is well researched and often found to be correlated with grade (56–58) and survival in glioma (56, 57) and glioblastoma (56, 64) patients with moderate grade differentiating abilities (56, 58) and moderate prognostic abilities (HR=1.5) (64). Contrary, anti-clotting factors were also found to be increased (see **Supplemental Tables 3**, **4** and **6**). Here, it may be possible that anti-clotting factors are reactively increased as a response to the prothrombotic state that is created by the tumor. However, it is also possible that the tumor stimulates the increase in anti-thrombotic proteins, as these may facilitate metastasis by degrading the extracellular matrix and allowing tumor cells to invade blood vessels (119).

### Panels of Peptides and Proteins

Biomarker panels of two to over 100 markers were used with various purposes in glioma patients. In general, larger panels could differentiate between patients and controls or different patient groups with different grades, tumor types or survival with higher accuracies. Inflammation, immune response and cell proliferation related markers were dysregulated such as interleukins (13), TNF-alfa (13), CRP (52), YKL-40 (86) and FGF-basic (13). Functional analysis revealed enrichment of pathways that are dysregulated in cancer cells such as apoptosis pathways, immune function pathways and others (13, 52). Several protein and peptide panels could differentiate between glioma patients and healthy individuals with high accuracies with sensitivities and specificities >85% (13, 52, 86, 120–123). Only two panels had modest value as diagnostic markers with an AUC of 0.6 (16) and 74% accuracy (124). One protein panel (122) and several proteins or peptides from other panels (120, 123) could also differentiate between glioma patients with different tumor grades. Lastly, panels could differentiate between patients with better and worse prognosis (16, 125), and between patients with different intracranial tumors (123).

## Nucleic Acids

### MicroRNAs

miRNAs are short, single-stranded RNAs of approximately 22 nucleotides in length, which bind and regulate translational repression or degradation of messenger and other RNAs (126). MicroRNAs may be ideal blood-based biomarkers as they are easily accessible in body fluids (127), are stable under harsh extrinsic conditions such as significant changes in temperature (128), and are protected from intrinsic conditions such as degradation by RNAses (129). Indeed, microRNAs can be found in biofluids such as serum, plasma, urine and cerebrospinal fluid and have shown to be deregulated in various cancer types such as renal cell carcinoma (130) and melanoma (131). Also, research is accumulating indicating that the blood of glioma patients has a unique miRNA expression pattern. However, it has been noted that miRNAs may not be good biomarkers as the brain has little influence on miRNA concentrations in blood as compared to other organs (132) and because differences in blood cell counts may more prominently influence variation in circulating miRNA profiles (133, 134). Despite that, miR-21 (135–143), miR-182 (144–148) and miR-222 (139, 149, 150) were all found to be increased in the blood of glioma patients as compared to healthy individuals. However, miR-21 (151, 152) and miR-222 (136) were also found to not have significantly different results in patients compared to controls. Diagnostic sensitivities and specificities of the miRNAs in glioma patients ranged from 47% to perfect accuracy (136, 139, 141, 148, 149). miR-21 (136, 139), miR-182 (146, 147) and miR-222 (139) were also correlated with tumor grade and an AUC of 0.8 was reported for miR-21 (139). Furthermore, miR-21 (143) and miR-222 (139) might also have use as a marker to differentiate between glial tumors and other intracranial tumors. Lastly, miR-21 (141), miR-182 (148, 153) and miR-222 (149, 150) may have value as prognostic markers and HRs of 1.3 (148) and 2.8 (149) have been reported. Remarkably, it was also found that miR-21 was upregulated years before glioma manifestation in patients (154).

### Panels of microRNAs

In general, combination of microRNAs increased the accuracies of markers as compared to single microRNAs. Small panels of microRNAs which studied marker concentrations in two or three microRNAs could differentiate between glioma patients and controls with an AUC of 0.8 (139) and sensitivities and specificities between 70%-100% (138, 155–157). When larger miRNA panels were used, diagnostic accuracies of tests tended to increase. Using a panel of nine miRNAs as diagnostic markers, 50 and 90 glioma patients could be differentiated from healthy individuals with high accuracy with an AUC of 1.0 (151) and accuracy of 99.8% (137), respectively. However, a 180-miRNA panel in whole blood could distinguish between glioblastoma patients and healthy individuals with ‘just’ 81% accuracy (158). Patients with glioblastoma could be differentiated from lower grade patients with an AUC of 0.9 (159), also certain miRNA combinations were highly prognostic for glioma patients with HRs of 3.1 (151) and 0.4 (160), or could differentiate between patients with different brain tumors with an AUC of 0.8 (157). Lastly, the development of pulmonary embolisms could be predicted in glioma patients with an AUC of 0.8 (161).

### Cell-Free DNA

Cell-free DNA (cfDNA) refers to fragmented DNA freely circulating outside of cells in blood plasma. cfDNA partly consists of DNA derived from tumor cells. cfDNA is often analyzed by examining circulating DNA from patients and searching whether there are tumor-specific mutations, deletions and/or amplifications present. The majority of cfDNA is released by non-tumor cells including (neighboring) inflammatory, stromal and other (healthy) cells, thereby searching for tumor-derived materials is considered to be a needle in a haystack. While both serum and plasma were used as biosource for cfDNA, it has been reported that serum contains around six times as much amounts of free cfDNA as compared to plasma with low levels of contaminating extraneous DNA released from leukocytes (162). Evidence is accumulating that the amount of cfDNA molecules and individual sequences of cfDNA can be employed as tumor biomarkers. First, it has been shown that total number of cfDNA molecules can be used as diagnostic marker to differentiate between glioma patients and controls (163, 164), tumor type differentiating marker (163), tumor progression marker (165), and prognostic marker (164). However, it remains unclear whether total cfDNA can also be used as a marker to estimate and monitor tumor burden (164–166). Furthermore, mutations and copy number variations in cfDNA can also be utilized to differentiate between glioma patients and controls (164, 166–170). Diagnostic sensitivities ranged from 50% to near perfect accuracies. Especially selection of cfDNA fragments between 90-150 base-pairs drastically improved detection accuracies. Moreover, mutations in therapeutically relevant genes such as TP53 and EGFR could also be found in cfDNA (164, 167) but were not always concordant with mutations in tumor tissue.

An alternative and highly potential biomarker may be methylation patterns in cfDNA of glioma patients. DNA methylation is one of three epigenetic mechanisms used to alter gene expression and can contribute to cancer development through regional hypermethylation and global hypomethylation (171). Methylation of tumor suppressor genes can silence tumor suppressor genes, while global hypomethylation of repetitive genomic elements can lead to elevated expression of oncogenes and chromosomal instability (171). In cfDNA of glioma patients, global hypomethylation of repetitive Alu elements and regional methylation of tumor suppressor genes such as MGMT were studied. Global hypomethylation of Alu elements was correlated with glioma diagnosis, higher tumor grade, shorter survival and lower Karnofsky Performance Score (172, 173). Also, it was recently reported that cfDNA methylation profiles have remarkable diagnostic capabilities in high-grade as well as in low-grade glioma with AUCs near 1.0. cfDNA methylation profiles also displayed high brain tumor differentiating capabilities with AUCs between 0.7-0.8 (174). Concordance of promoter methylation in tumor suppressor genes such as MGMT in cfDNA with their counterparts inside tumors, was observed with varying sensitivities 31%-80% but with high specificities all near 100% (175–182). Lastly, lack of MGMT promoter methylation in cfDNA could be used as a prognostic marker with hazard ratios between 2.0-2.2 (180, 182). While cfDNA methylation methods are of interest as markers with multiple purposes, so far the patient populations in which these methods were studied were often small (50 or less patients).

## Circulating Cells, Extracellular Vesicles, and Metabolomics

### Circulating Glioma Cells

It has been suggested that circulating tumor cells (CTCs) are the driving cells of tumor metastasis. Extracranial metastases occur very rarely in patients with glioma and with an (estimated) incidence of less than 0.5% (183, 184). Despite this, several research efforts have been investigating the existence of circulating glial tumor cells (CGTCs) using a variety of methods, with highly variable results. Diagnostic sensitivities were reported between 21%-80% (185–192). Apart from diagnosis, CGTCs might also have other purposes. However, the cells were often not correlated with tumor grade (187, 190, 192), survival (188) or tumor burden (189) and they could not be used to differentiate between different glial tumor types (187). Interestingly, CGTCs have also been investigated as a tool to differentiate between tumor recurrence and radiation necrosis (192), and to differentiate between pseudoprogression and actual tumor progression (186, 190, 191), though such applications are definitely not ready for implementation in the current daily clinics.

### Blood Platelets

It is well documented that platelets influence cancer cells in multiple ways, for example, platelets are known to promote tumor angiogenesis, tumor cell proliferation, metastasis and aid in immune surveillance escape of tumor cells (193). Because platelets stimulate tumor activities to a large degree, it is possible that platelet counts and content are altered as well in patients with glioma. However, currently platelet counts have variable results as biomarkers in glioma. Platelet counts were found to be increased in glioma patients (93, 100, 194) as well as non-significantly changed (54, 55, 118, 194) compared to healthy individuals. In most studies, platelets were observed to be non-significantly altered in patients with higher grade glioma compared to lower grade glioma (54, 58, 93, 195–197). Furthermore, there is overwhelming evidence that platelet counts are not correlated with patient prognosis in glioma patients (62, 70, 71, 93, 198–205). Moreover, platelet counts are not different in glioma patients as compared to other intracranial pathologies such as epilepsy and non-glial brain tumors (54, 206, 207). Aside platelet counts, researchers, amongst us, have noted that platelets may have altered protein content (208) and RNA content, due to sequestration of tumor-derived RNAs. The RNA content of these so-called ‘tumor-educated-platelets’ (TEPs) may be employed to distinguish cancer patients from healthy individuals (209). Also other research groups have confirmed that TEPs have good accuracy in distinguishing between healthy individuals and patients with various types of cancer (210–214). There are many obstacles that can interfere with the results of the TEPs such as age-related factors (215, 216), pre-analytical variables, and inflammatory and cardiovascular disease (217). Despite this, our data suggests that platelet RNA profiles may be employed for diagnostics of lower-grade glioma and glioblastoma, and potentially tumor treatment monitoring (209, 218, 219). Hence, platelets may contain promising information regarding the presence and treatment response of glioma.

### White Blood Cells

In glioma patients it was often observed that WBC counts were increased compared to controls (21, 54, 55, 100, 118, 220). However, it was also found that leukocytes are not significantly changed in glioma patients compared to controls (21, 194), potentially due to dexamethasone use (221–224). Furthermore, it remains unclear whether WBCs are correlated with higher tumor grades (54, 55, 195, 201, 225, 226) and worse survival (23, 61, 62, 71, 201, 227) as many studies reported both statistically significant and non-significant results (144). Moreover, it was found that leukocyte counts were increased in glioma patients compared to patients with neuromas (54), non-lesional epilepsy (54) and meningioma (54, 161) and lack of IDH-mutation (201). However, it was also found that WBCs are not different in glioma as compared to meningioma patients (55). At this moment, total white blood cell counts are not considered promising as a blood-based marker for glioma.

### Lymphocytes

Lymphocytes mainly consist out of three groups: T-cells (CD-3+), B-cells (CD-20+) and NK-cells (CD-56+). It was found in multiples studies that total lymphocyte numbers are not changed in glioma patients as compared to controls (21, 55, 100, 194, 228, 229). However, significant decreases in total lymphocytes were noted as well in glioma patients (54, 206, 228). This significant decrease might be attributed partly due to the use of dexamethasone (229). Total lymphocyte counts were lower in patients with higher tumor grades (54, 55, 196, 197, 201) and one study reported a tumor grade differentiating AUC of 0.6. Also, many studies reported that total lymphocyte numbers were not correlated with survival in glioma patients (62, 70, 71, 74, 198, 201, 202, 204, 205, 227, 230–233) though two studies reported that increased numbers of total lymphocytes were associated with prolonged survival (234, 235). Furthermore, total lymphocytes were not changed in glioma patients as compared to patients with brain metastases (206, 207), but it remains unclear whether total lymphocytes are changed in glioma patients as compared to meningioma (54, 55, 206) and epilepsy patients (54, 206). However, lymphocytes were also not correlated to tumor grade in two other studies (58, 196) or with IDH-1/2 mutation status (201, 236).

Total T-cells numbers were seen to be significantly decreased in glioma patients in multiple studies with high statistical significance (20, 21, 23, 229, 237, 238). Two other studies did not find a difference in malignant glioma patients (21, 24). Corticosteroids usually did not influence total T-cell counts (21, 23, 223), however, in one study corticosteroids did cause a significant decrease in CD3+-cell counts (229). Thus, more research is needed to determine whether CD3+ cells are altered in glioma patients. It may be possible that T-cells are decreased because of a decrease in CD4+-cells, which has often been reported in glioma patients (20, 21, 23, 229, 238–240). However, other studies did not find a significant difference between glioma patients and controls (21, 241, 242) in terms of CD4+-counts in blood. CD4+-cells have been found to be negatively correlated with glial tumor grade (20, 243) as well as to not be correlated with increasing tumor grades in glioma patients. Decrease in CD4+-cell counts was inversely related to survival in glioma patients (233, 243), but not related to IDH1-status (236).

There is little evidence for NK-cells as blood-based glioma biomarker. In several studies NK-cells (CD3+/CD56+, CD3-/CD56+ or CD16+/CD56+) were not significantly altered as compared to healthy individuals in glioma patients (23, 229, 244, 245). However, certain NK-cell populations were seen to be significantly decreased (23, 245) or increased (238) in glioma patients. Also, CD16+/CD56+-NK-cells had prognostic value (23). CD8+-cell counts were not altered in glioma patients in most studies (21, 238, 239, 242). It remains unclear whether CD8-cell counts are correlated with patient survival (23, 233) and lower tumor grades (20). There is a lot of controversial evidence concerning the value of lymphocytes and subpopulations of lymphocytes as biomarkers in glioma and glioblastoma patients. However, the majority of studies agree that total T-cells and CD4+-cells may be promising as a diagnostic marker.

### Neutrophils

Neutrophils were found to be increased in glioma patients compared to controls in the majority of the studies (21, 54, 55, 194, 220). Furthermore, higher-grade glioma patients were often reported to have increased neutrophil counts as compared to patients with lower-grade glioma (54, 55, 58, 196, 197, 201, 226, 246). Grade differentiating AUCs between 0.6-0.7 were reported (55, 58, 201). It remains unclear whether neutrophils are related to IDH mutation status (201, 236). Moreover, glioma patients had higher neutrophils compared to patients with a meningioma (54, 161), neuromas (54) or epilepsy (54, 206). It was also found that there was no difference between glioma or glioblastoma patients and meningioma patients (55, 206), between glioma or glioblastoma and metastases (206, 207) and grade III and grade IV glioma patients (227) in terms of neutrophil counts. Multiple studies reported that neutrophil counts had no prognostic value in glioma patients (62, 70, 71, 198, 204, 205, 230, 234), however, other studies found a negative correlation of neutrophil counts with survival in glioma (201, 227, 231, 247) with HRs around 1.6 (227, 231). Thus, neutrophil count might be valuable as diagnostic and grade differentiating marker.

### Monocytes

It remains unclear whether monocyte counts (CD14+-cells and/or CD16+-cells) are changed in glioma patients compared to controls (54, 55, 106, 194, 229, 244, 248) or are related to tumor grade (54, 55, 58, 197, 225, 246). However, monocytes with reduced immune function and with mainly immunosuppressive functions such as M2-macrophages (245, 249, 250) and HLA-DR-low and HLA-DR-negative monocytes were significantly increased in glioma patients as compared to controls (21, 244, 251), but cell counts might be confounded by dexamethasone use (229). Also, less pro-inflammatory M1-macrophages were observed in glioma patients (249, 250). Total monocyte counts could not be correlated to prognosis (62, 71, 227, 231). Monocytes were found to not be different in glioblastoma patients as compared to patients with brain metastases (207) and increased in glioma patients compared to epilepsy (54), meningioma (54, 55), or acoustic neuroma (54).

### Neutrophil-Lymphocyte-Ratio

The Neutrophil-lymphocyte-ratio (NLR) may be a promising marker for multiple types of cancers (252–255) and has the potential to fulfill various biomarker roles. It is unclear how the NLR can be dysregulated. However, a hypothesis is that tumors, including glioblastoma (256), secrete hematopoietic factors such as granulocyte-colony stimulating factor, granulocyte macrophage-colony stimulating factor and IL-1 and IL-6, which stimulate proliferation of neutrophils (257, 258). Also, tumors can secrete neutrophil attractant chemokines (259) and turn neutrophils from foe into friend *via* the secretion of TGF-beta (260). Tumor-associated neutrophils can stimulate vascularization of the tumor and inhibit lymphocyte function, weakening the antitumor response (261). NLR and the derived NLR (dNLR; absolute neutrophil count/(WBC count minus absolute neutrophil count) were increased in glioma patients compared to controls (54, 55, 194, 206, 262). Glioma patients with low NLR or derived NLR had longer survival in multiple studies with large patient populations (57, 63, 64, 69, 75, 93, 201, 203, 204, 225, 226, 230, 234, 262–269) with HRs mostly between 1.7 and 2.4. On the contrary, multiple studies including those with larger patient populations could not find a correlation between NLR and survival in glioma patients (62, 71, 198, 200, 205, 227, 247, 270). Furthermore, there is overwhelming evidence that NLR is significantly increased in patients with higher-grade glioma as compared to patients with lower-grade glioma (54–57, 93, 195, 197, 200, 201, 213, 266, 271, 272). The AUC to differentiate between patients with higher-grade and lower-grade glioma was mostly between 0.6 and 0.7. It remains unclear whether NLR values are increased in glioma patients compared to patients with meningioma (54, 55, 206) or intracerebral metastases (206, 207), although it may be increased as compared to patients with epilepsy (54, 206) or acoustic neuroma (54). Also, NLR values might be correlated with IDH-mutation status (56, 63, 69, 201, 225, 226, 267) and increased tissue Ki-67 expression (267, 271). Finally, high NLR correlated with tumor relapse (264), and decrease in NLR during treatment with radiotherapy and concomitant temozolomide was correlated with pseudoprogression (265). To conclude, NLR might be correlated with clinicopathological markers, survival and tumor grade. However, there is a lot of conflicting evidence for most of these markers. There is overwhelming evidence that NLR is related to tumor grade, but the accuracies reported are too limited to apply NLR as a definite diagnostics biomarker in the clinics.

### Platelet-Lymphocyte-Ratio and Monocyte-Lymphocyte Ratio

It remains unclear whether the platelet-lymphocyte-ratio (PLR) and monocyte-lymphocyte-ratio may have use as a blood-based marker in glioma. Controversial results have been found for both PLR (54, 55, 206) and MLR (54, 194) as diagnostic markers to differentiate between glioma patients and controls. Similar controversial results have also been found for PLR (54–56, 197, 201, 263, 266, 271) and MLR (54, 58, 197, 266, 271) as tumor grade differentiating markers. PLR (63, 64, 69, 71, 75, 198, 204, 205, 263, 266, 267, 269) as well as MLR (69, 71, 75, 267, 271) were found to not be prognostic in glioma patients in the majority of studies. However, PLR and MLR might have some value as brain disease differentiating marker for glioma patients as these markers were significantly different in glioma patients as compared to patients with epilepsy (54, 206) and non-glial brain malignancies metastases (54, 55, 206, 207). Both markers were rarely correlated with tumor tissue IDH-mutation (56, 63, 69, 201, 267) or MGMT-methylation status, and Ki-67 proliferation index (267, 271).

### Systemic Immune Inflammation Index

The systemic immune inflammation (SII) index can be calculated as follows: platelets * (neutrophils/lymphocytes). A high SII-index was correlated with short survival in patients with different cancer types (273–275). In glioma, an increased SII-index was found in patients with higher tumor grades (58, 70, 195, 196) with AUCs of 0.6-0.8 (58, 196), respectively. The SII-index was correlated with poorer prognosis (70, 267), and patients with tumors with higher tissue Ki-67 proliferation index (196), but was not correlated with tumor size (70).

### Dendritic Cells

Dendritic cells are antigen presenting cells that can present antigens for example from tumor cells to T-cells, which subsequently activates these T-cells. Total dendritic cells and its subpopulations (myeloid/conventional dendritic cells and plasmacytoid dendritic cells) were found to be decreased in blood of glioma patients compared to controls (21, 243, 276), and these cell populations were also decreased in glioblastoma patients compared to patients with lower tumor grades (243). Furthermore, it was reported that an immature dendritic cell population with increased immunoinhibitory effects on cells (277) becomes increased in glioma patients, especially in patients with higher tumor grades (243). Therefore, glial tumors might actively weaken a patient’s immune system.

### Myeloid-Derived Suppressor Cells

Myeloid-derived suppressor cells (MDSCs) are immunoinhibitory cells originating from monocytes. MDSCs might be formed during direct cell-cell contact with tumor cells possibly during infiltration of the glial tumor (106). There are variable results concerning in which glioma patients MDSC counts are changed. Total MDSCs (33, 278–280), monocytic MDSCs (21, 33, 280, 281) and granulocytic MDSCs (33, 278–281) were often significantly increased in glioblastoma patients but non-significantly altered in patients with lower grades. Furthermore, MDSCs were increased in patients with poor prognosis (30) and in glioblastoma patients as compared to other patients with intracranial tumors such as anaplastic glioma or meningioma (251).

### Regulatory T-Cells

Tregs are known for their immunosuppressive functions (282) and have been shown to be associated with poor patient prognosis in various cancer types (283). Tregs have been found to be significantly increased in the blood of glioblastoma patients as compared to healthy individuals (23, 229, 240, 284, 285). However, Tregs were also found to be non-significantly altered in glioma (21, 23, 241, 286) or even significantly decreased (237, 240).

### Extracellular Vesicles

Extracellular vesicles (EVs) are microparticles that are 30 to 10.000 nanometer in diameter. These vesicles are released by cells and can carry proteins, lipids and nucleic acids from one cell to another, thereby facilitating communication between cells (287). Extracellular vesicles can be released from the plasma membrane itself as microvesicles or can be released after fusion of endosomes inside a cell with the plasma membrane as exosomes (288). In glioma patients numbers of EVs (289, 290), microparticles (291) and exosomes (292) in blood were increased as compared to healthy individuals. EVs could potentially also be used as markers for tumor relapse (289) or tumor progression as opposed to pseudoprogression (293). Furthermore, the cargo of EVs can be employed as biomarkers. The protein cargo level – that is the total amount of protein loaded – from glioma patients might have value as diagnostic marker (294, 295). Also, the protein cargo itself is dysregulated and can be used to differentiate between a group of healthy individuals and patients with less malignant glial tumors, which have similar protein cargo, and patients with highly malignant glial tumors (296). EV protein cargo from glioblastoma patients was enriched in proteins that were associated with inflammation, immune response, members of the complement coagulation cascade and others (289). Other studies found a decrease in immune system related proteins IFN-γ, IL-10, and IL-3 within plasma exosomes from glioma patients (292). Furthermore, RNA inside exosomes may increase tumor cell invasion and repress apoptosis (297). Lastly, the surface protein profile of EVs are dysregulated (298, 299) and can be used as biomarkers to differentiate between glioma patients and healthy individuals with high accuracy.

### Single Metabolites and Metabolomic Panels

Metabolomics is the analysis of small molecules in a biofluid, cell, tissue, organ or organism (300) and can be used to study metabolic pathways within the organism. Combinations of metabolites such as creatine, glucose and lactate could differentiate patients with brain tumors, glioblastoma, oligodendroglioma, glial tumor, or astrocytoma from healthy individuals with very high accuracy (AUC: 0.9-1.0) (301). Patients with higher grade and lower grade tumors could be differentiated with AUC of 0.7 (301) or 91% accuracy (302). Tumor type differentiating metabolomic panels had variable accuracies with AUCs between 0.4-0.8 (301, 303). Tumor tissue IDH-mutation status could be predicted with an accuracy of 94% (302). Single metabolites (303, 304) and metabolite combinations (303) could predict survival of glioma patients, even with near perfect accuracies. Remarkably, serum metabolites such as tocopherols were found in two studies that could predict glioblastoma up to 22 years before manifestation (305) and glioma patients up to 9 years before manifestation (306). The metabolic pathways that were dysregulated were often involved energy metabolism including amino acid metabolism (302, 303, 306, 307), lipid metabolism (303, 306, 307), nucleic acid metabolism (302) and carbohydrate metabolism (302, 306, 307). Glucose and lactate in particular are interesting markers and had value as blood biomarkers with several purposes, possibly due to their role in the Warburg effect. Glucose levels were reported to be increased in patients with higher tumor grades (308) or worse survival (308–311). One study reported that this was independent of the degree of disability, tumor grade, diabetes, prolonged dexamethasone use, or subsequent treatment modalities (309). Furthermore, it has been found that glucose was related to tumor progression and it was higher in patients with glial brain tumors such as glioblastoma and oligodendrogliomas, but not in meningioma, as compared to healthy individuals (301). Pre-treatment lactate levels (302, 312, 313) were increased in patients with high-grade glioma compared to low-grade glioma patients with AUCs of 0.7 (312) and 1.0 (313), and could potentially also be used as a diagnostic marker (307).

### Assessment of Risk of Bias and Reproducibility of Included Studies

Using summaries of the methodology and results of the studies that we referenced here (see **Supplemental Tables 3**–**9**), we assessed risk of bias in the biomarker studies, similar to some degree to the QUADAS-2 (314) and REMARK (315) guidelines for quality assessment of diagnostic and prognostic biomarker studies. We noted several limitations in the studies that were reviewed concerning study population size, presentation of results and registration of effect of intrinsic and extrinsic factors that could influence marker levels. Apart from markers that are measured on a routine basis such as some APRPs or inflammatory cell populations in clinical chemistry labs, the study populations of markers are often small (often <100 individuals included). Also, small validation cohorts are used or validation cohorts were not included at all. Primarily glioblastoma patients are included in the studies that we referenced here. Patients with lower-grade gliomas are rarely included or comprise a small portion of the entire patient population. Therefore, it is unclear whether the biomarkers that we selected as being most promising, will be of value in particularly these patients. Furthermore, the majority of studies only reported the p-values of biomarkers and not the value of biomarkers quantified as accuracy, sensitivity, specificity and/or hazard ratio, and these could not be deduced from the available and presented data. Therefore, it is unclear what the clinical value of most biomarkers is. Lastly, it is largely unknown to what extent biomarkers are affected by extrinsic factors such as anti-tumor therapy, use of (co-)medication, choice of analytical methods, and by intra-individual factors such as race, comorbidities and others. Co-medication use by patients before sampling, such as corticosteroid use, is reported in some studies. Use of other medication, e.g. anti-diabetic and anti-epileptic drugs, is rarely reported. Most studies that reported use of these drugs did not (statistically) analyze the effect of these drugs on the biomarkers that were studied, a potential bias that should be taken into account when interpreting these data.

In order to assess the potential reproducibility, and ultimately clinical validation, of the most promising markers GFAP, IL-10, and miR-21, we precisely evaluated the available studies using dedicated guidelines. The MIQE guideline was employed for the miRNA (316), whereas we had to adjust existing guidelines to assess the studies for GFAP and IL-10 (**Supplementary Table 12**), as to the best of our knowledge no such guidelines are available for ELISA/immunoassays. As can be seen in **Supplementary Figure 1**, for GFAP and IL-10 essential factors of the study design such as the number of included patients, protein detection methods and kits were almost always mentioned. However, other important factors such as the used (analytical) instrumentation, sample storage and sample preparation procedures were rarely reported. Also, it was often not reported whether samples were quality controlled by evaluating intra-assay and inter-assay variability. Furthermore, test accuracy is often not reported which makes it unclear whether the tests will have value in the clinical settings. Lastly, factors such as comedication use, histopathological marker presence and tumor volume are rarely reported, which can have significant impact on biomarker concentrations. For miR-21, several categories of the guidelines were often sufficiently described such as experimental design, sample processing and storage. However, other categories such as ‘nucleic acid extraction’, ‘qPCR target information’ and ‘qPCR protocol’ were rarely sufficiently described or not described at all (**Supplementary Figure 2**). In all, it again highlights that adequate reporting of employed methods is of importance to ensure reproducibility of the identified biomarker.

Thus, it can be concluded that there is room for improvement in biomarker studies in multiple domains of methodology and results presentation, as has been reported by other (systematic) reviews (317–319). The biomarker studies that we referenced here may not be of the highest possible quality and cannot be used to determine immediately which biomarkers will have clinical value. However, they can still be used to determine which biomarkers are promising for further research, as markers that have shown great clinical group differentiating abilities in multiple studies may still hold clinical value despite the bias in results and methodologies present in the studies.

## Discussion

Glioma is still one of the most devastating diseases with high burden. Any additional information that can be obtained from the patient regarding tumor development, growth, behavior, and vulnerabilities, in a least minimally invasive way is desired. Many studies have been published, and included in this systematic review, that identified potential circulating biomarkers for patients with glioma, at each point in the glioma patients clinical course (**Figure 1**). Unfortunately, none of the biomarkers is in our opinion ready for direct clinical implementation.

**Figure 4:**
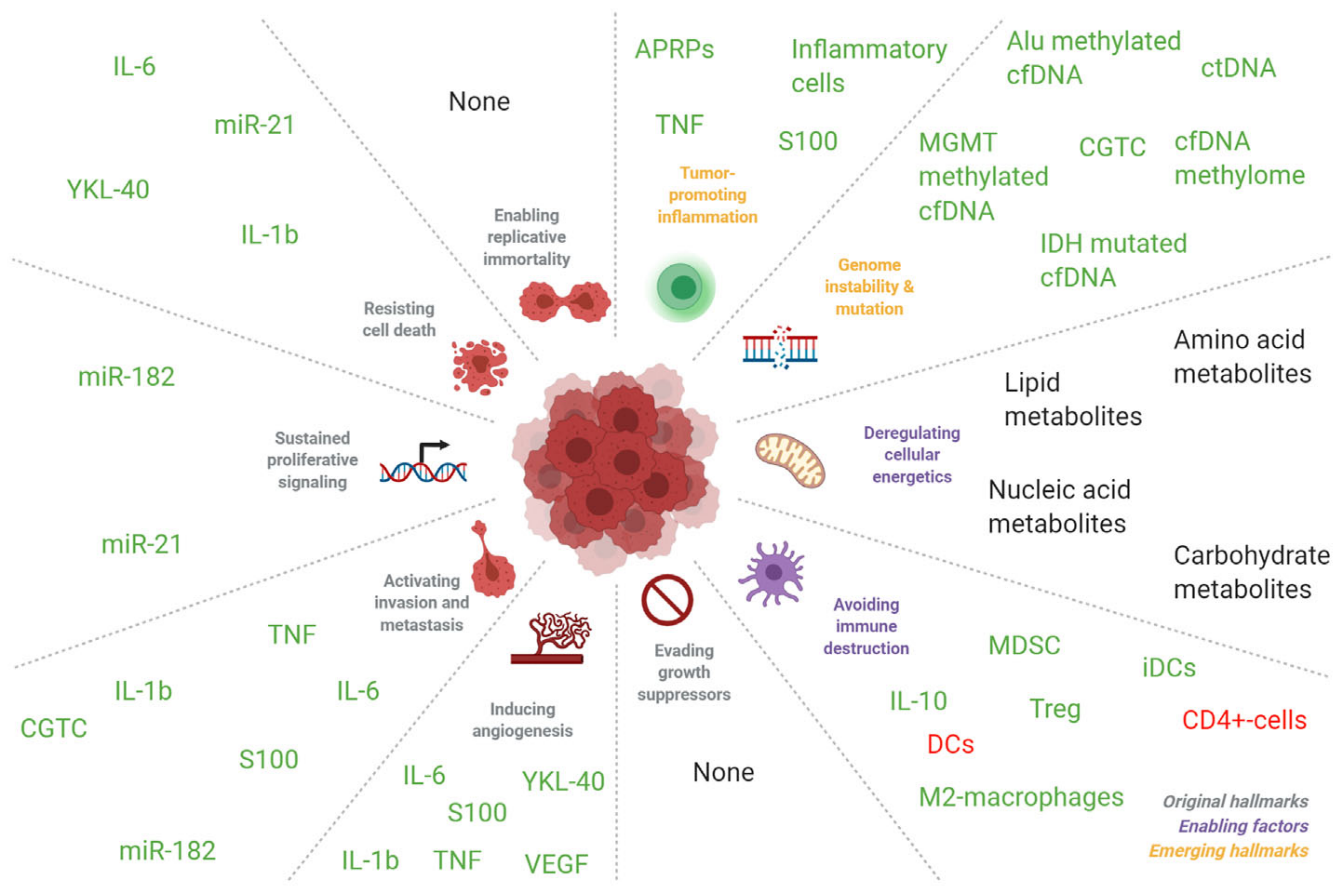
Correlation of blood-based biomarkers in patients with glioma with seminal events in tumorigenesis. Blood biomarkers in glioma patients are implicated in the molecular pathways as detailed by Hanahan and Weinberg (321). Markers colored in green were mostly found to be increased in the circulation of glioma patients compared to healthy individuals, and in glioma patients with more malignant tumors compared to patients with less malignant tumors. Markers that were inversely correlated were colored in red. Markers without color were found to be either significantly increased or decreased in the formerly mentioned groups. The abbreviation “DC” indicates dendritic cells and “iDC” indicates immature dendritic cells. Adapted from “Hallmarks of Cancer: Circle”, by BioRender.com (2021). Retrieved from: https://app.biorender.com/biorender-templates.

As opposed to tumor tissue biomarkers, such as MGMT methylation, 1p/19q codeletion, IDH1-mutations, and the recently introduced methylation profiling (320) in glioma, blood-based markers often reflect local or systemic responses of the endogenous processes to the presence of a tumor. Direct measurement of glioma-derived circulating cells and genomic aberrations is an exception in this view. Cells, cell ratios and APRPs that are often measured in complete blood counts (CBCs), are attractive biomarkers as CBCs are regularly used in the clinic and extensive research has already been performed on their utility as biomarkers. However, they are possibly insufficiently accurate biomarkers for clinical utility as a single marker or in combination with other cells, cell ratios or APRPs. An explanation for the worse performance of single markers as opposed to panels of markers may be interpreted using the framework of hallmarks and enabling characteristics of cancer, as formulated in the seminal article by Hanahan and Weinberg (321). Hallmarks are traits unique to cancer cells and enabling characteristics are traits that lead to the development of such hallmarks. In this framework, blood biomarkers including VEGF, miR-182 and YKL-40 may be mediating factors that enable cancer cells to contain the hallmarks ‘inducing angiogenesis’, ‘resisting cell death’ and ‘tissue invasion’. Other biomarkers such as lactate concentrations and CGTC can be seen as an expression of the hallmarks or enabling characteristics ‘deregulated cellular energetics’ and ‘activating invasion and metastasis’. Inflammatory cells may be promoting the enabling characteristic ‘tumor promoting inflammation’ (see **Supplemental Table 11** and **Figure 4**). As single blood markers have low to modest accuracies and value as biomarkers but panels of biomarkers often have higher accuracies, it can be hypothesized that screening of multiple markers involved in multiple hallmarks or enabling characteristics may improve biomarker accuracy. This hypothesis can be supported by the fact that diagnostic sensitivity of inflammatory cells such as NLR, PLR, neutrophils and others is limited with diagnostic and tumor grade and tumor type differentiating AUCs between 0.6-0.7 (54–56, 58, 201). Similar results also have been found for APRPs with AUCs between 0.5-0.7 (54–56, 58). Combination of inflammatory cell populations (54, 56, 58) or APRPs (56), as well as combination of inflammatory cell populations with APRPs (54, 56), does not increase accuracy in a meaningful way (144). Thus, it is possible that both APRPs as well as inflammatory cell populations already reflect alterations in the inflammation enabling characteristic and combination of these markers does not further improve marker accuracies. Also, panels of biomarkers often have higher accuracies than single biomarkers irrespective of the biomarker function and these panels contain biomarkers involved in multiple pathways related to the hallmarks of cancer and its enabling characteristics (13, 52, 155). Furthermore, our analysis indicates that biomarker levels become increasingly dysregulated as tumors increase in malignancy, as biomarker levels are often positively correlated with tumor grade, worse survival and/or more malignant tumor types. Therefore, cellular hallmarks might develop in more cancer cells as tumor malignancy increases and this may be reflected in the dysregulation of circulating biomarkers. With this in mind, we propose to introduce multi-biosource, multi-biomolecule-based blood tests for glioma patients. Keeping the criteria for biomarker test development as discussed above in mind, such tests may likely include (components of) a miRNA, protein, or platelet RNA panel, perhaps including the already promising single markers miR-21, IL-10, and GFAP. These panels likely include multiple components of the tumor progression, are less resistant to confounding variables due to its high dimensions, and far more accurate than a single measured biomarker. Also, complementary implementation of several biomarker types may make synergistically use of each other’s advantages, and perhaps at least partially reduce each other’s disadvantages (**Table 1**) (144).

**Table 1:** Advantages and disadvantages of the biomarkers.

Nucleic acids (miRNA, cfDNA, RNA, DNA methylation)	
Advantages	Disadvantages
If well-designed highly specific	Long turn-around when using next-generation sequencing approaches
For certain methods such as digital droplet PCR highly sensitive, also depending on patient population and tumor stage	Expensive test requirements, esp. with next-generation sequencing
Measurements can be multiplexed and analysis of panels is possible	Requires high-quality RNA isolates
Well-established isolation and detection methods	Clonal hematopoiesis may confound mutation analysis
Provides information on (epi)genomic and transcriptomic levels	May not provide actionable information
**Proteins and peptides**	
**Advantages**	**Disadvantages**
Long-term experience with protein-based tests in current clinical practice	Can be less specific
Usually low costs for tests	Limited stability
Easily standardized protocols	
Sensitive test methodologies	
**Circulating cells (white blood cells, blood platelets, lymphocytes, etc.)**	
**Advantages**	**Disadvantages**
Measurement routinely available in clinical chemistry labs	Reduced specificity
Rapid test results	No direct measurement of tumor-derived materials; surrogate markers
	Some circulating cells, esp. immune cells, require more specialized isolation and quantification methodologies
**Circulating glioma cells and extracellular vesicles**	
**Advantages**	**Disadvantages**
Directly tumor-derived markers, therefore highly specific	May require expensive, technically-challenging, and time-consuming isolation procedures
Enables for testing of panels of (genetic) markers	No gold standard for isolation
Protects markers from degrading enzymes in plasma	Reduced sensitivity, esp. in lower tumor stages
Circulating glioma cells may allow for functional analysis and drug screens	Long turn-around when using next-generation sequencing approaches

Hence, additional validation of the currently most promising markers (**Figure 4**) is also required. Aside analysis of blood, other biofluids such as urine or perhaps cerebrospinal fluid may also be rich sources of biomarkers. Recent analysis has shown that tumor evolution could be tracked *via* repeated CSF samplings (322). Similarly, perhaps also other body fluids such as saliva, sputum, or breathing air may contain molecular information traceable to a primary glioma. We believe that blood-based biomarkers may currently only at maximum complement the current methods to diagnose and/or monitor a glioma, such as clinical symptoms, imaging, and tissue collection *via* tumor resection or (stereotactic) biopsy. It may very well be anticipated that blood-based biomarkers are included in a future setting in clinical decision making, for example in multidisciplinary tumor boards, once such biomarkers are thoroughly validated. For this, systematic biobanking of blood from glioma patients is required. Such biobanking requires research funds that support these efforts, as well as research project that in a dedicated way screen for relevant and valuable biomarkers in well-annotated, large, and homogeneous patient series. It is of importance that any future biomarker discovery or validation research is reported according to the highest standards, facilitating reproducibility of the found results. Alternatively, we believe that any clinical trial, even in a phase 1 stage, should include a blood-biomarker branch in the trial design, in order to at least aim to discover a companion diagnostics biomarker. Also, blood-based biomarkers that may complement current imaging methods for the identification of true tumor progression versus pseudo-tumor progression is required.

In all, the glioma research community should be encouraged towards additional identification and inclusion of blood-based biomarker research in a clinical setting. While currently at the stage of analytical validation and start of clinical validation, further studies should focus on demonstrating its clinical utility.

## Data Availability Statement

The original contributions presented in the study are included in the article/**Supplementary Material**. Further inquiries can be directed to the corresponding author.

## Author Contributions

HA, RH, and MGB developed the search strategy and developed the selection criteria. HA, RH, and MA selected studies for inclusion through title, abstract, and full-text screening. HA, TW, and MGB drafted the manuscript. All authors contributed to the article and approved the submitted version.

## Funding

Financial support was provided by stichting STOPHersentumoren.nl (TW and MGB).

## Conflict of Interest

The authors declare that the research was conducted in the absence of any commercial or financial relationships that could be construed as a potential conflict of interest.
