# Supplementary material for: Blood-Based Biomarkers for Glioma in the Context of Gliomagenesis: A Systematic Review
Source: Front Oncol. 2021 Jun 4;11:665235. doi: 10.3389/fonc.2021.665235 (PMC8211985; doi:10.3389/fonc.2021.665235)
Supplement: Supplementary file 1 [file Table_1.docx]

Index of supplementary material

[Supplemental Table 1: Pubmed search results 1](#_Toc63446737)

[Supplemental Table 2: Embase search results 2](#_Toc63446738)

[Supplemental Table 3: Diagnostic marker table 5](#_Toc63446739)

[Supplemental Table 4: Tumor grade-differentiating marker table 29](#_Toc63446740)

[Supplemental Table 5: Table for differentiating markers of glial tumors compared to other intracranial pathologies 40](#_Toc63446741)

[Supplemental Table 6: Prognostic marker table 50](#_Toc63446742)

[Supplemental Table 7: Predictive marker table 64](#_Toc63446743)

[Supplemental Table 8: Therapy monitoring marker table for tumor volume 70](#_Toc63446744)

[Supplemental Table 9: Therapy monitoring marker table for tumor progression 72](#_Toc63446745)

[Supplemental Table 10: Panels of Biomarkers 73](#_Toc63446746)

[Supplementary table 11: Glioma biomarkers involved in hallmarks or enabling characteristics of cancer 79](#_Toc63446747)

# Supplemental Table 1: Pubmed search results

**PubMed Session Results (07 Aug 2020)**

| Search | Query | Items found |
| --- | --- | --- |
| #7 | **#6 NOT ("Animals"[Mesh] NOT "Humans"[Mesh])** | 3,596 |
| #6 | **#4 AND #5** | 3,996 |
| #5 | **"prognos*"[tiab] OR "diagnos*"[tiab] OR "monitor*"[tiab] OR "predict*"[tiab] OR "screen*"[tiab] OR "detect*"[tiab] OR "identif*"[tiab] OR "marker*"[tiab] OR "biomarker*"[tiab]** | 9,082,471 |
| #4 | **#1 AND #2 AND #3** | 6,412 |
| #3 | **"Biomarkers"[Mesh] OR "biomarker*"[tiab] OR "bioindicator*"[tiab] OR "biological indicator*"[tiab] OR "marker*"[tiab] OR "surrogate endpoint*"[tiab] OR "surrogate end point*"[tiab] OR "Neoplastic Cells, Circulating"[Mesh] OR "Nucleic Acids"[Mesh] OR "circulating tumor*"[tiab] OR "circulating tumour*"[tiab] OR "Neoplasm Circulating Cell*"[tiab] OR "Neoplastic Circulating Cell"[tiab] OR "Neoplastic Circulating Cells"[tiab] OR "Circulating Neoplastic Cell*"[tiab] OR "Cell Free Nucleic Acid*"[tiab] OR "Circulating Nucleic Acid*"[tiab] OR "DNA"[tiab] OR "RNA"[tiab] OR "mirna"[tiab] OR "microRNA"[tiab] OR "cfDNA"[tiab] OR "cirDNA"[tiab] OR "cfRNA"[tiab] OR "cirRNA"[tiab] OR "protein"[tiab] OR "proteins"[tiab] OR "Metabolome"[Mesh] OR "metabolom*"[tiab] OR "metabolic profile*"[tiab]** | 5,370,388 |
| #2 | **"Liquid Biopsy"[Mesh] OR "Blood"[Mesh] OR "blood"[Subheading] OR "plasma"[tiab] OR "serum"[tiab] OR "serologic"[tiab] OR "blood"[tiab] OR "hemostat*"[tiab] OR "haemostat*"[tiab] OR "hematol*"[tiab] OR "haematol*"[tiab] OR "circulat*"[tiab]** | 4,835,066 |
| #1 | **"Glioma"[Mesh] OR "glioma*"[tiab] OR "astrocytoma*"[tiab] OR "glioblastoma*"[tiab] OR "ependymoma*"[tiab] OR "ganglioglioma*"[tiab] OR "gliosarcoma*"[tiab] OR "medulloblastoma*"[tiab] OR "medullo-blastoma*"[tiab] OR "oligodendroglioma*"[tiab]** | 114,557 |

# Supplemental Table 2: Embase search results

**Embase.com Session Results (07 Aug 2020)**

| Search | Query | Items found |
| --- | --- | --- |
| #8 | **#7 NOT (conference*:it OR letter*:it OR editorial*:it)** | 4,323 |
| #7 | **#6 NOT ([animals]/lim NOT [humans]/lim)** | 7,292 |
| #6 | **#4 AND #5** | 8,465 |
| #5 | **prognos*:ab,ti,kw OR diagnos*:ab,ti,kw OR monitor*:ab,ti,kw OR predict*:ab,ti,kw OR screen*:ab,ti,kw OR detect*:ab,ti,kw OR identif*:ab,ti,kw OR marker*:ab,ti,kw OR biomarker*:ab,ti,kw** | 12,003,907 |
| #4 | **#1 AND #2 AND #3** | 12,367 |
| #3 | **'biological marker'/exp OR 'tumor marker'/exp OR biomarker*:ab,ti,kw OR bioindicator*:ab,ti,kw OR 'biological indicator*':ab,ti,kw OR marker*:ab,ti,kw OR 'surrogate endpoint*':ab,ti,kw OR 'surrogate end point*':ab,ti,kw OR 'nucleic acid'/exp OR 'circulating tumor*':ab,ti,kw OR 'circulating tumour*':ab,ti,kw OR 'Neoplas* Circulating Cell*':ab,ti,kw OR 'Circulating Neoplas* Cell*':ab,ti,kw OR 'Cell Free Nucleic Acid*':ab,ti,kw OR 'Circulating Nucleic Acid*':ab,ti,kw OR DNA:ab,ti,kw OR RNA:ab,ti,kw OR mirna:ab,ti,kw OR microRNA:ab,ti,kw OR cfDNA:ab,ti,kw OR cirDNA:ab,ti,kw OR cfRNA:ab,ti,kw OR cirRNA:ab,ti,kw OR protein:ab,ti,kw OR proteins:ab,ti,kw OR 'metabolome'/exp OR metabolom*:ab,ti,kw OR 'metabolic profile*':ab,ti,kw** | 6,480,344 |
| #2 | **'liquid biopsy'/exp OR 'blood'/exp OR plasma:ab,ti,kw OR serum:ab,ti,kw OR serologic:ab,ti,kw OR blood:ab,ti,kw OR hemostat*:ab,ti,kw OR haemostat*:ab,ti,kw OR hematol*:ab,ti,kw OR haematol*:ab,ti,kw OR circulat*:ab,ti,kw** | 6,018,486 |
| #1 | **'glioma'/exp OR glioma*:ab,ti,kw OR astrocytoma*:ab,ti,kw OR glioblastoma*:ab,ti,kw OR ependymoma*:ab,ti,kw OR ganglioglioma*:ab,ti,kw OR gliosarcoma*:ab,ti,kw OR medulloblastoma*:ab,ti,kw OR 'medullo blastoma*':ab,ti,kw OR oligodendroglioma*:ab,ti,kw** | 173,663 |

# Supplemental Table 3: Diagnostic marker table

| Marker (reference number) | Biosource | Glial tumor grades of glioma patients in study population if specified | Control population size | Biomarker detection methodology | Significantly increased | Significantly decreased | Non-significantly changed | Marker AUC, accuracy or sensitivity (SE) and specificity (SP) if measured | Medication use before blood sampling in all or some patients if reported |
| --- | --- | --- | --- | --- | --- | --- | --- | --- | --- |
| IL-1b (1) | Serum | II: 24; III: 22; IV:148 | 26 | Bead array | X |  |  |  |  |
| IL-1b (2) | Serum | 32 glioma patients | 32 | ELISA | X |  |  |  |  |
| IL-1b (3) | Plasma | 120 glioma patients | 120 | ELISA | X |  |  |  |  |
| IL-1b (4) | Serum | IV: 13 | 13 | ELISA | X |  |  |  |  |
| IL-1b (5) | Serum | IV: 55 | 20 | ELISA | X |  |  |  |  |
| IL-1b (6) | Plasma | III: 8; IV: 53 | 9 | Immunoassay |  |  | X |  | Corticosteroid were used by some patients |
| IL-1b (7) | Plasma | IV: 15 | 15 | ELISA |  |  | X |  |  |
| IL-6 (8) | Serum | IV: 148, II: 24, III: 22 | 26 | ELISA | X |  |  | AUC: 0.9 |  |
| IL-6 (9) | Serum | I-II: 18; III: 25; IV: 43 | 18 | ELISA | X |  |  |  |  |
| IL-6 (3) | Plasma | 120 glioma patients | 120 | ELISA | X |  |  |  |  |
| IL-6 (1) | Serum | II: 24; III: 22; IV:148 | 26 | Bead array | X |  |  |  |  |
| IL-6 (5) | Serum | IV: 55 | 20 | ELISA | X |  |  |  |  |
| IL-6 (10) | Serum | IV: 47 | 60 | ELISA | X |  |  |  | Corticosteroid were used by some patients |
| IL-6 (11) | Plasma | IV: 30 | 20 | ELISA | X |  |  |  |  |
| IL-6 (12) | Plasma | IV: 18 | 17 | Antibody microarray | X |  |  |  | Corticosteroid were used by some patients |
| IL-6 (13) | Serum | I-II: 18; III-IV: 20 | 26 | ELISA |  | X |  |  |  |
| IL-6 (14) | Plasma | IV: 7 | 7 | Cytometric bead array |  | X |  |  | Corticosteroid were used by some patients |
| IL-6 (15) | Serum | I; 2; II: 14; III:2; IV: 17 | 20 | ELISA |  |  | X |  |  |
| IL-6 (16) | Serum | III-IV: 44 | 44 | ELISA |  |  | X |  |  |
| IL-6 (17) | Serum | IV: 50 | 25 | ATR-FTIR spectroscopy |  |  | X |  |  |
| IL-6 (6) | Plasma | III: 8; IV: 53 | 9 | Immunoassay |  |  | X |  | Corticosteroid were used by some patients |
| IL-6 (18) | Plasma | II-IV: 158 | Not specified | ELISA |  |  | X |  | Corticosteroid were used by some patients |
| IL-10 (8) | Serum | IV: 148, II: 24, III: 22 | 26 | ELISA | X |  |  | AUC: 1.0 |  |
| IL-10 (17) | Serum | IV: 50 | 25 | ATR-FTIR spectroscopy |  |  | X |  |  |
| IL-10 (19) | Serum | IV: 20 | 1278 | Immunoassay | X |  |  | SE: 95%; SP: 85% |  |
| IL-10 (20) | Serum | II: 16; III: 7; IV: 26 | 30 | ELISA | X |  |  |  | Corticosteroids or anti-epileptic drugs were not used by patients |
| IL-10 (6) | Plasma | III: 8; IV: 53 | 9 | Immunoassay | X |  |  |  | Corticosteroid were used by some patients |
| IL-10 (1) | Serum | II: 24; III: 22; IV:148 | 26 | Bead array | X |  |  |  |  |
| IL-10 (5) | Serum | IV: 55 | 20 | ELISA | X |  |  |  |  |
| IL-10 (14) | Plasma | IV: 7 | 7 | Cytometric bead array | X |  |  |  | Corticosteroid were used by some patients |
| IL-10 (21) | Plasma | IV: 51 | 36 | ELISA | X |  |  |  | Corticosteroid were used by some patients |
| S100a8 (22) | Serum | IV: 125 | 42 | ELISA | X |  |  |  |  |
| S100a8 (23) | Serum | IV: 35 | 30 | ELISA | X |  |  |  |  |
| S100a9 (22) | Serum | IV: 125 | 42 | ELISA | X |  |  |  |  |
| S100a9 (23) | Serum | IV: 35 | 30 | ELISA | X |  |  |  |  |
| S100a9 (24) | Serum | IV: 10 | 10 | ELISA | X |  |  |  |  |
| S100a9 (25) | Serum | IV: 17 | 17 | Antibody array | X |  |  |  | No drugs used one month prior to sampling |
| S100a8/9 (26) | Serum | II: 8, III: 13, IV:20 | 17 | ELISA | X |  |  |  | Corticosteroid were used by some patients |
| S100b (27) | Serum | III-IV: 14 | 14 | ELISA | X |  |  |  |  |
| S100b (28) | Serum | III: 4; IV: 27 | Not specified | Turbidimetric method |  |  | X |  | Corticosteroid were used by some patients |
| S100 (29) | Serum | III: 6; IV: 42 | 69 | Immunoenzyme assay | X |  |  |  |  |
| S100 (29) | Serum | III: 6; IV: 42 | 69 | Immunoenzyme assay | X |  |  |  |  |
| TNF-alfa (5) | Serum | IV: 55 | 20 | ELISA | X |  |  |  |  |
| TNF-alfa (10) | Plasma | IV: 47 | 60 | ELISA | X |  |  |  | Corticosteroid were used by some patients |
| TNF-alfa (30) | Plasma | IV: 42 | 6 | Bead-based flow cytometry assay | X |  |  |  | Corticosteroid were used by some patients |
| TNF-alfa (3) | Plasma | 120 glioma patients | 120 | ELISA | X |  |  |  |  |
| TNF-alfa (20) | Serum | II: 16; III: 7; IV: 26 | 30 | ELISA |  | X |  |  | Corticosteroids or anti-epileptic drugs were not used by patients |
| TNF-alfa (12) | Plasma | IV: 18 | 17 | Antibody microarray |  | X |  |  | Corticosteroid were used by some patients |
| TNF-alfa (1) | Serum | II: 24; III: 22; IV:148 | 26 | Bead array |  |  | X |  |  |
| TNF-alfa (31) | Serum | IV: 14 | 32 | ELISA |  |  | X |  |  |
| TNF-alfa (8) | Serum | IV: 148, II: 24, III: 22 | 26 | ELISA |  |  | X |  |  |
| TNF-beta (1) | Serum |  |  |  |  |  | X |  |  |
| sTNF-R1 (32) | Plasma | IV: 112 | 58 | ELISA |  | X |  |  | Corticosteroid were used by some patients |
| sTNF-R2 (32) | Plasma | IV: 112 | 58 | ELISA |  | X |  |  | Corticosteroid were used by some patients |
| Haptoglobin (33) | Serum | I-III: 10; IV: 8 | 36 | Radial immunodiffusion | X |  |  |  | Corticosteroid were used by some patients |
| Haptoglobin (34) | Serum | IV: 16 | 9 | Radial immunodiffusion | X |  |  |  |  |
| Haptoglobin (35) | Serum | II: 26; III: 49; IV: 113 | 32 | ELISA | X |  |  | AUC: 0.8 |  |
| Haptoglobin (36) | Serum | IV: 40 | 40 | Western blot | X |  |  |  | Corticosteroid were used by some patients |
| CRP (33) | Serum | I-III: 10; IV: 8 | 36 | Radial immunodiffusion | X |  |  |  |  |
| CRP (37) | Serum | IV: 28 | 27 | Antibody microarray | X |  |  | AUC: 0.8 |  |
| CRP (10) | Plasma | IV: 47 | 60 | ELISA | X |  |  |  | Corticosteroid were used by some patients |
| CRP (38) | Plasma | IV: 14 | 15 | Quantitative targeted absolute proteomics | X |  |  | AUC: 0.8 |  |
| CRP (39) | Serum | IV: 91; III: 15; II: 36 | 58 | Turbidimetric method |  |  | X |  | Corticosteroid were used by some patients |
| Alpha 1- antichymotrypsin (36) | Serum | IV: 40 | 40 | Western blot | X |  |  |  | Corticosteroid were used by some patients |
| Alpha 1- antichymotrypsi n (38) | Plasma | IV: 14 | 15 | Quantitative targeted absolute proteomics | X |  |  |  |  |
| Alpha 1- antitrypsin (34) | Serum | IV: 16 | 9 | Radial immunodiffusion | X |  |  |  |  |
| Alpha 1- antitrypsin (33) | Serum | I-III: 10; IV: 8 | 36 | Radial immunodiffusion | X |  |  |  |  |
| A2 macroglobulin (19) | Serum | IV: 20 | 1278 | Immunoassay | X |  |  |  |  |
| A1 acid glycoprotein (34) | Serum | IV: 16 | 9 | Radial immunodiffusion | x |  |  |  |  |
| A1 acid glycoprotein (33) | Serum | I-III: 10; IV: 8 | 36 | Radial immunodiffusion | X |  |  |  |  |
| IL-1Ra (1) | Serum | II: 24; III: 22; IV:148 | 26 | Bead array | X |  |  |  |  |
| IL-1Ra (8) | Serum | IV: 148, II: 24, III: 22 | 26 | ELISA | X |  |  | AUC: 0.8 |  |
| Ceruloplasmin (36) | Serum | IV: 40 | 40 | Western blot | X |  |  |  | Corticosteroid were used by some patients |
| Ferritin (40) | Serum | IV: 57 | Not specified | TINA-Quant ferritin assay | X |  |  |  |  |
| Ferritin (21) | Plasma | IV: 51 | 36 | ELISA | X |  |  |  | Corticosteroid were used by some patients |
| Fibrinogen (19) | Plasma | III: 5; IV: 23 | 367 | Immunoassay | X |  |  |  |  |
| Fibrinogen (10) | Serum | IV: 47 | 60 | ELISA | X |  |  |  | Corticosteroid were used by some patients |
| Complement c4 (20) | Serum | II: 16; III: 7; IV: 26 | 30 | ELISA |  | X |  |  | Corticosteroids or anti-epileptic drugs were not used by patients |
| Complement factor b (12) | Plasma | IV: 18 | 17 | Antibody microarray |  | X |  |  | Corticosteroid were used by some patients |
| Complement c5 (12) | Plasma | IV: 18 | 17 | Antibody microarray | X |  |  |  | Corticosteroid were used by some patients |
| Complement component C9 (38) | Plasma | IV: 14 | 15 | Quantitative targeted absolute proteomics | X |  |  |  |  |
| Albumin (41) | Serum | I: 81; II: 208; III: 169; IV: 292 | 682 | Not specified |  | X |  |  |  |
| Albumin (42) | Serum | I-II: 39; III-IV: 34 | 49 | Not specified |  |  | X |  | Corticosteroid were used by some patients |
| Prognostic nutritional index (41) | Serum | I: 81; II: 208; III: 169; IV: 292 | 682 | Not specified |  |  |  |  |  |
| Prognostic nutritional index (42) | Serum | I-II: 39; III-IV: 34 | 49 | Not specified |  |  |  |  | Corticosteroid were used by some patients |
| Albumin globulin ratio (42) | Serum | I-II: 39; III-IV: 34 | 49 | Not specified |  |  |  |  | Corticosteroid were used by some patients |
| GFAP (29) | Serum | III: 6; IV: 42 | 69 | Immunoenzyme assay |  |  |  | SE: 83% in glioblastoma patients  SE: 75% in high-grade glioma patients |  |
| GFAP (43) | Serum | IV: 14 | 13 | ELISA | X |  |  | AUC: 0.9 in glioblastoma patients; SE: 86% in glioblastoma patients; SP: 85% in glioblastoma patients | Corticosteroid were used by some patients |
| GFAP (44) | Serum | IV: 50 | 50 | ELISA | X |  |  | SE: 76%  SP: 100%  AUC: 0.9 | Corticosteroid were used by some patients |
| GFAP (43) | Serum | III: 13 | 13 | ELISA |  |  | X | SE: 23% in anaplastic glioma patients |  |
| GFAP (44) | Serum | II: 17; III: 14 | 50 | ELISA |  |  | X | SE: 0% | Corticosteroid were used by some patients |
| GFAP (45) | Serum | IV: 33 | Not specified | immunofluorescence assay | X |  |  | SE: 42% |  |
| GFAP (45) | Serum | I-III: 4 | Not specified | immunofluorescence assay |  |  | X |  |  |
| GFAP (46) | Serum | IV: 91 | ELISA |  | X |  |  | SE: 89% in glioblastoma  SP: 96% |  |
| GFAP (46) | Serum | II-III: 39 | ELISA |  |  |  | X | SE: 26% in astrocytoma grade II-III |  |
| GFAP (47) | Plasma | IV: 111 | 99 | ELISA | X |  |  |  |  |
| GFAP (48) | Plasma | IV: 34 | 26 | ELISA | X |  |  | SE: 38%; SP: 96% |  |
| GFAP (48) | Plasma | I-II: 7; III: 10 | 26 | ELISA |  |  | X | SE: 0%; SP: 96% |  |
| YKL-40 (49) | Serum | III: 14; IV: 22 | 33 | ELISA | X |  |  |  |  |
| YKL-40 (51) | Plasma | II:23; III:19; IV:30 | 30 | ELISA | X |  |  | AUC: 0.9  SE: 81%  SP: 83 | Corticosteroid were used by some patients |
| YKL-40 (47) | Plasma | IV: 111 | 99 | ELISA | X |  |  |  |  |
| YKL-40 (149) | Serum | II-III: 20; IV: 45 | 14 | ELISA |  |  |  |  |  |
| YKL-40 (197) | Serum | IV 60 | 22 | Immunoassay |  |  |  |  |  |
| VEGF (2) | Serum | 32 glioma patients | 32 | ELISA | X |  |  |  |  |
| VEGF (52) | Serum | I; 12; II:18; III:19; IV: 11 | 30 | ELISA | X |  |  |  |  |
| VEGF (53) | Serum | I-II: 7; III-IV: 23 | 50 | ELISA | X |  |  |  |  |
| VEGF (54) | Plasma | III-IV: 78 | 34 | ELISA | X |  |  |  |  |
| VEGF (55) | Plasma | III: 4; IV: 21 | 23 | ELISA | X |  |  |  |  |
| VEGF (5) | Serum | IV: 55 | 20 | ELISA | X |  |  |  |  |
| VEGF (10) | Serum | IV: 47 | 60 | ELISA | X |  |  |  | Corticosteroid were used by some patients |
| VEGF (56) | Serum | IV: 12 | 10 | ELISA | X |  |  |  |  |
| VEGF (57) | Serum | IV: 27 | 27 | ELISA | X |  |  |  |  |
| VEGF (58) | Serum | IV: 17 | 8 | ELISA | X |  |  |  | Corticosteroid were used by some patients |
| VEGF (12) | Plasma | IV: 18 | 17 | Antibody microarray | X |  |  |  | Corticosteroid were used by some patients |
| VEGF (59) | Plasma | IV: 70 | 23 | ELISA | X |  |  |  |  |
| VEGF (60) | Serum | II: 7 | 3 | ELISA |  |  | X |  |  |
| VEGF (61) | Serum | I-II: 3; III: 7; IV: 5 | 5 | ELISA |  |  | X |  |  |
| VEGF (62) | Serum | I: 2; II:1; IV: 6 | 145 | ELISA |  |  | X |  |  |
| VEGF (7) | Plasma | IV: 15 | 15 | ELISA |  |  | X |  |  |
| VEGF (14) | Plasma | IV: 7 | 7 | Cytometric bead array |  |  | X |  | Corticosteroid were used by some patients |
| VEGF (63) | Serum | IV: 36 | 5 | Cytokines Antibody Array |  |  | X |  | Corticosteroid were used by some patients |
| Endogen thrombin generation (10) | Whole blood | IV: 47 | 60 | ELISA | X |  |  |  | Corticosteroid were used by some patients |
| Prothrombin factor 1+2 (10) | Plasma | IV: 47 | 60 | ELISA | X |  |  |  | Corticosteroid were used by some patients |
| Prothrombin preprotein (24) | Plasma | IV: 2 | 2 | iTRAQ and LC-MS/MS | X |  |  |  |  |
| Tissue factor antigen (64) | Plasma | 24 glioma | 30 | ELISA | X |  |  |  |  |
| P-selectin (65) | Serum | IV: 21 | 21 | ELISA | X |  |  |  | Dexamethason, levitiracetam, thrombosisprophylaxis with low molecular weight heparin and proton-pump-inhibitors were used by some patients |
| Tissue factor (10) | Plasma | IV: 47 | 60 | ELISA | X |  | X |  | Corticosteroid were used by some patients |
| Plasminogen activator inhibitor (66) | Serum | I-II: 26; III-IV: 31 | 34 | ELISA | X |  |  |  | Corticosteroids were not used by patients |
| Factor VII (19) | Plasma | III: 5; IV: 23 | 367 | Immunoassay | X |  |  |  |  |
| Factor VII (19) | Serum | IV: 20 | 1278 | Immunoassay | X |  |  |  |  |
| Platelet Factor 4 (23) | Serum | IV: 35 | 30 | ELISA | X |  |  |  |  |
| Tissue factor pathway inhibitor (64) | Plasma | 24 glioma | 30 | ELISA | X |  |  |  |  |
| Protein c (64) | Plasma | 24 glioma | 30 | ELISA | X |  |  |  |  |
| Plasminogen precursor (36) | Serum | IV: 40 | 40 | Western blot | X |  |  |  | Corticosteroid were used by some patients |
| Antithrombin III (36) | Serum | IV: 40 | 40 | Western blot | X |  |  |  | Corticosteroid were used by some patients |
| Plasminogen (36) | Serum | IV: 40 | 40 | Western blot | X |  |  |  | Corticosteroid were used by some patients |
| Plasminogen isoform 1 precursor (24) | Plasma | IV: 2 | 2 | iTRAQ and LC-MS/MS | X |  |  |  |  |
| Protein S alpha preprotein (24) | Plasma | IV: 2 | 2 | iTRAQ and LC-MS/MS | X |  |  |  |  |
| MicroRNA-21 (68) | Serum | III: 5; IV: 23 | 10 | RT-PCR | X |  |  |  |  |
| MicroRNA-21 (69) | Plasma | II: 10; III: 10; IV: 10 | 10 | qRT-PCR | X |  |  | Auc: 0.9; SE: 90%; SP: 100% |  |
| MicroRNA-21 (70) | Serum | I-II: 47; IV: 44 | 17 | Droplet digital PCR | X |  |  | AUC: 0.6 |  |
| MicroRNA-21 (71) | Plasma | II: 6; III: 8; IV: 16 | 30 | qRT-PCR | X |  |  |  |  |
| MicroRNA-21 (72) | Serum | I: 2; II: 13; III: 16; IV: 69 | 30 | qRT-PCR | X |  |  | SE: 75% in LGG  SE: 81% in HGG  SE: 84% in glioblastoma  SP: 47% in LGG  SP: 77% in HGG  SP: 77% in glioblastoma |  |
| MicroRNA-21 (73) | Serum | III-IV: 15 | 11 | qRT-PCR | X |  |  |  |  |
| MicroRNA-21 (74) | Serum | IV: 20 | 20 | qRT-PCR | X |  |  | AUC: 1.0 |  |
| MicroRNA-21 (69) | Plasma | II: 10; III: 10; IV: 10 | 10 | qRT-PCR | X |  |  |  |  |
| MicroRNA-21 (75) | Plasma | IV: 10 | 10 | qRT-PCR | X |  |  |  |  |
| MicroRNA-21 (76) | Plasma | IV: 25 | 25 | qRT-PCR | X |  |  |  |  |
| MicroRNA-21 (77) | Serum | II: 28; III: 38; IV: 24 | 110 | qRT-PCR |  |  | X |  |  |
| MicroRNA-21 (78) | Serum | I-II: 25; III-IV: 45 | 25 | qRT-PCR |  |  | X |  |  |
| MicroRNA-182 (79) | Serum | 6 glioma patients | 3 | Electrochemical assay | X |  |  |  |  |
| MicroRNA-182 (80) | Serum | 6 glioma patients | 3 | Electrochemical assay | X |  |  |  |  |
| MicroRNA-182 (81) | Serum | 6 glioma patients | 3 | Electrochemical assay | X |  |  |  |  |
| MicroRNA-182 (82) | Serum | I-II: 24; III-IV: 30 | 10 | RT-PCR | X |  |  |  |  |
| MicroRNA-182 (83) | Plasma | I: 18; II: 23; III: 32; IV: 39 | 54 | qRT-PCR | X |  |  | AUC: 0.8 in glioma patients  SE: 59% in glioma patients  SP: 85% in glioma patients  AUC: 0.8 in HGG  AUC: 0.6 in LGG |  |
| MicroRNA-222 (72) | Serum | I: 2; II: 13; III: 16; IV: 69 | 30 | qRT-PCR | X |  |  |  |  |
| MicroRNA-222 (85) | Plasma | 50 glioma patients | 51 | qRT-PCR | X |  |  |  |  |
| MicroRNA-222 (69) | Plasma | II: 10; III: 10; IV: 10 | 10 | qRT-PCR |  |  | x |  |  |
| MicroRNA-222 (205) | Serum | IV 20 | 20 | qRT-PCR |  |  |  | SE: 85;  SP: 100 |  |
| Total number of cfDNA (86) | Serum | IV: 122 | 130 | Fluorimetry | X |  |  |  |  |
| Total number of cfDNA (87) | Plasma | IV: 42 | 42 | qRT-PCR | X |  |  | AUC: 0.99 |  |
| Cfdna methylome (88) | Plasma | 112 glioma patients | 59 | Illumina HumanBeadChip 850K array | X |  |  |  |  |
| Alu methylation (89) | Serum | I-II: 38; III-IV: 71 | 50 | DNA sequencing |  | X |  |  |  |
| Alu methylation (90) | Serum | I-II: 32; III-IV: 33 | 30 | DNA sequencing |  | X |  |  |  |
| ALU247/ALU11 5 ratio (91) | Serum | I: 3; II: 35; III: 14; IV: 18 | 22 | qPCR |  |  | X |  |  |
| Presence of circulating tumor DNA (92) | Plasma | IV: 34 | 65 | Whole-genome sequencing | X |  |  |  |  |
| Circulating glial tumor cells (93) | Whole blood | 10 glioma patients | 1 | Immuno-precipitation | X |  |  | SE: 80%; SP: |  |
| Circulating glial tumor cells (95) | Whole blood | II: 11; III: 9; IV: 12 | 178 | Immunofluorescence | X |  |  | SE: 59%; SP: 100% |  |
| Circulating glial tumor cells (96) | Whole blood | II-IV: 11 | 30 | telomerase promoter-based assay | X |  |  | SE: 72%; SP: 100% |  |
| Circulating glial tumor cells (97) | Whole blood | II: 11; III: 9; IV: 11 | 10 | SE-iFISH | X |  |  | SE: 77%; SP: 100% |  |
| Circulating glial tumor cells (98) | Whole blood | IV: 141 | 23 | Fluorescence immunocyto-chemistry | X |  |  | SE: 21%; SP: 100% |  |
| Circulating glial tumor cells (99) | Whole blood | IV: 33 | 6 | STEAM immunofluorescence | X |  |  | SE: 39%; SP: 100% |  |
| Circulating glial tumor cells (100) | Whole blood | IV: 13 | 5 | Immuno-precipitation | X |  |  | SE: 60%; SP: 100% |  |
| Circulating glial tumor cells (101) | Whole blood | IV: 13 | 3 | Immunostaining | X |  |  | SE: 54%; SP: 100% |  |
| Blood platelets (41) | Whole blood | I: 81 | 682 | Hematology analyzer | X |  |  |  |  |
| Blood platelets (51) | Whole blood | II:23; III:19; IV:30 | 30 | Hematology analyzer | X |  |  | AUC: 0.8 | Corticosteroid were used by some patients |
| Blood platelets (54) | Whole blood | III-IV: 78 | 34 | Hematology analyzer | X |  |  |  |  |
| Blood platelets (102) | Whole blood | 70 glioma patients | 216 | Hematology analyzer | X |  |  |  | Corticosteroids were not used by patients |
| Blood platelets (41) | Whole blood | IV: 292 | 682 | Hematology analyzer |  |  | X |  |  |
| Blood platelets (42) | Whole blood | I-II: 39; III-IV: 34 | 49 | Hematology analyzer |  |  | X |  | Corticosteroid were used by some patients |
| Blood platelets (41) | Whole blood | I: 81; II: 208; III: 169; IV: 292 | 682 | Hematology analyzer |  |  | X |  |  |
| Blood platelets (54) | Whole blood | III-IV: 78 | 34 | Hematology analyzer |  |  | X |  |  |
| Blood platelets (65) | Whole blood | IV: 21 | 21 | Hematology analyzer |  |  | X |  | Dexamethason, levitiracetam, thrombosisprophylaxis with low molecular weight heparin and proton-pump-inhibitors were used by some patients were used by some patients |
| Blood platelet RNA | Whole blood | IV: 347 | 306 | RNA sequencing |  |  |  | 1.0 |  |
| Blood platelet RNA | Whole blood | IV: 39 | 55 | RNA sequencing |  |  |  | 0.9 |  |
| White blood cells (6) | Whole blood | IV: 53 | 9 | Flow cytometry | X |  |  |  | Corticosteroid were used by some patients |
| White blood cells (41) | Whole blood | I: 81; II: 208; III: 169; IV: 292 | 682 | Hematology analyzer | X |  |  |  |  |
| White blood cells (42) | Whole blood | I-II: 39; III-IV: 34 | 49 | Hematology analyzer | X |  |  |  | Corticosteroid were used by some patients |
| White blood cells (54) | Whole blood | III-IV: 78 | 34 | Hematology analyzer | X |  |  |  |  |
| White blood cells (65) | Whole blood | IV: 21 | 21 | Hematology analyzer | X |  |  |  | Dexamethason, levitiracetam, thrombosisprophylaxis with low molecular weight heparin and proton-pump-inhibitors were used by some patients |
| White blood cells (106) | Whole blood | I-II: 31; III-IV: 74 | 50 | Hematology analyzer | X |  |  |  |  |
| White blood cells (6) | Whole blood | III: 8 | 9 | Flow cytometry |  |  | X |  | Corticosteroid were used by some patients |
| White blood cells (102) | Whole blood | 70 glioma patients | 216 | Hematology analyzer |  |  | X |  | Corticosteroids were not used by patients |
| Lymphocytes (41) | Whole blood | I: 81; II: 208; III: 169; IV: 292 | 682 | Hematology analyzer |  | X |  |  |  |
| Lymphocytes (107) | Whole blood | III-IV: 59 | 77 | Hematology analyzer |  | X |  |  |  |
| Lymphocytes (108) | Whole blood | IV: 37 | 30 | Hematology analyzer |  | X |  |  |  |
| Lymphocytes (6) | Whole blood | III: 8 | 9 | Flow cytometry |  |  | X |  | Corticosteroid were used by some patients |
| Lymphocytes (42) | Whole blood | I-II: 39; III-IV: 34 | 49 | Hematology analyzer |  |  | X |  | Corticosteroid were used by some patients |
| Lymphocytes (54) | Whole blood | III-IV: 78 | 34 | Hematology analyzer |  |  | X |  |  |
| Lymphocytes (102) | Whole blood | 70 glioma patients | 216 | Hematology analyzer |  |  | X |  | Corticosteroids were not used by patients |
| Lymphocytes (107) | Whole blood | I-II: 72 | 77 | Hematology analyzer |  |  | X |  |  |
| Lymphocytes (6) | Whole blood | IV: 53 | 6 | Flow cytometry |  |  | X |  | Corticosteroid were used by some patients |
| Lymphocytes (110) | Whole blood | IV: 26 | 15 | Flow cytometry |  |  | X |  | Corticosteroid were used by some patients |
| Total t cells (6) | Whole blood | IV: 53 | 6 | Flow cytometry |  | X |  |  | Corticosteroid were used by some patients |
| Total t cells (20) | Whole blood | II: 16; III: 7; IV: 26 | 30 | Flow cytometry |  | X |  |  | Corticosteroids or anti-epileptic drugs were not used by patients |
| Total t cells (21) | Whole blood | IV: 51 | 36 | Flow cytometry |  | X |  |  | Corticosteroid were used by some patients |
| Total t cells (110) | Whole blood | IV: 26 | 15 | Flow cytometry |  | X |  |  | Corticosteroid were used by some patients |
| Total t cells (111) | Whole blood | IV: 65 | 94 | Quantitative methylation specific PCR |  | X |  |  | Corticosteroid were used by some patients |
| Total t cells (112) | Whole blood | IV: 19 | 13 | Flow cytometry |  | X |  |  |  |
| Total t cells (6) | Whole blood | III: 8 | 6 | Flow cytometry |  |  | X |  | Corticosteroid were used by some patients |
| Total t cells (7) | Whole blood | IV: 15 | 15 | Flow cytometry |  |  | X |  |  |
| CD4+ cells (20) | Whole blood | II: 16; III: 7; IV: 26 | 30 | Flow cytometry |  | X |  |  | Corticosteroids or anti-epileptic drugs were not used by patients |
| CD4+ cells (113) | Whole blood | I-II: 24; IV: 15 | 21 | Flow cytometry |  | X |  |  | Corticosteroid were used by some patients |
| CD4+ cells (6) | Whole blood | IV: 53 | 6 | Flow cytometry |  | X |  |  |  |
| CD4+ cells (21) | Whole blood | IV: 51 | 36 | Flow cytometry |  | X |  |  | Corticosteroid were used by some patients |
| CD4+ cells (110) | Whole blood | IV: 26 | 15 | Flow cytometry |  | X |  |  | Corticosteroid were used by some patients |
| CD4+ cells (112) | Whole blood | IV: 19 | 13 | Flow cytometry |  | X |  |  |  |
| CD4+ cells (114) | Whole blood | IV: 8 | 6 | Flow cytometry |  | X |  |  | Corticosteroid were used by some patients |
| CD4+ cells (6) | Whole blood | III: 8 | 6 | Flow cytometry |  |  | X |  | Corticosteroid were used by some patients |
| CD4+ cells (115) | Whole blood | IV: 11 | 16 | Flow cytometry |  |  | X |  |  |
| CD4+ cells (116) | Whole blood | IV: 35 | 15 | Flow cytometry |  |  | X |  | Corticosteroid were used by some patients |
| PD1+ CD4+- cells (117) | Whole blood | II:6; III: 3; IV:9 | 7 | Flow cytometry |  |  |  |  |  |
| Tim-3 CD4+- cells (117) | Whole blood | II:6; III: 3; IV:9 | 7 | Flow cytometry |  |  |  |  |  |
| NK-cells (112) | Whole blood | IV: 19 | 13 | Flow cytometry | X |  |  |  |  |
| CD3-CD56+ NK-cells (118) | Whole blood | 25 glioma patients | 17 | Flow cytometry |  | X |  |  |  |
| CD3/CD56 NK-cells (21) | Whole blood | IV: 51 | 36 | Flow cytometry |  | X |  |  | Corticosteroid were used by some patients |
| CD3+/CD56+ NK-cells (118) | Whole blood | 25 glioma patients | 17 | Flow cytometry |  |  | X |  |  |
| CD16/CD56 NK-cells (21) | Whole blood | IV: 51 | 36 | Flow cytometry |  |  | X |  | Corticosteroid were used by some patients |
| NK-cells (110) | Whole blood | IV: 26 | 15 | Flow cytometry |  |  | X |  | Corticosteroid were used by some patients |
| NK-cells (119) | Whole blood | IV: 15 | 10 | Flow cytometry |  |  | X |  | Corticosteroid were used by some patients |
| CD8+ cells (6) | Whole blood | III: 8; IV: 53 | 9 | Flow cytometry |  |  | X |  | Corticosteroid were used by some patients |
| CD8+ cells (112) | Whole blood | IV: 19 | 13 | Flow cytometry |  |  | X |  |  |
| CD8+ cells (113) | Whole blood | I-II: 24; IV: 15 | 21 | Flow cytometry |  |  | X |  | Corticosteroid were used by some patients |
| CD8+ cells (116) | Whole blood | IV: 35 | 15 | Flow cytometry |  |  | X |  | Corticosteroid were used by some patients |
| Neutrophils (41) | Whole blood | I: 81; II: 208; III: 169; IV: 292 | 682 | Not specified | X |  |  |  |  |
| Neutrophils (42) | Whole blood | I-II: 39; III-IV: 34 | 49 | Not specified | X |  |  |  | Corticosteroid were used by some patients |
| Neutrophils (102) | Whole blood | 70 glioma patients | 216 | Not specified | X |  |  |  | Corticosteroids were not used by patients |
| Neutrophils (106) | Whole blood | I-II: 31; III-IV: 74 | 50 | Not specified | X |  |  |  |  |
| Neutrophils (6) | Whole blood | IV: 53 | 9 | Flow cytometry | X |  |  |  | Corticosteroid were used by some patients |
| Neutrophils (41) | Whole blood | IV: 292 | 682 | Hematology analyzer | X |  |  |  |  |
| Neutrophils (102) | Whole blood | IV: 36 | 216 | Hematology analyzer | X |  |  |  | Corticosteroids were not used by patients |
| Neutrophils (106) | Whole blood | I-II: 31 | 50 | Hematology analyzer | X |  |  |  |  |
| Neutrophils (106) | Whole blood | III-IV: 74 | 50 | Hematology analyzer | X |  |  |  |  |
| Neutrophils (6) | Whole blood | III: 8 | 9 | Flow cytometry |  |  | X |  | Corticosteroid were used by some patients |
| Monocytes (41) | Whole blood | IV: 292 | 682 | Hematology analyzer | X |  |  |  |  |
| Monocytes (42) | Whole blood | I-II: 39; III-IV: 34 | 49 | Hematology analyzer | X |  |  |  | Corticosteroid were used by some patients |
| Monocytes (41) | Whole blood | I: 81; II: 208; III: 169; IV: 292 | 682 | Hematology analyzer | X |  |  |  |  |
| Monocytes (119) | Whole blood | IV: 15 | 10 | Flow cytometry | X |  |  |  | Corticosteroid were used by some patients |
| Monocytes (120) | Whole blood | II: 7; III: 8; IV: 54 | 24 | Flow cytometry | X |  |  |  |  |
| Monocytes (102) | Whole blood | 70 glioma patients | 216 | Hematology analyzer |  | X |  |  | Corticosteroids were not used by patients |
| Monocytes (102) | Whole blood | IV: 36 | 216 | Hematology analyzer |  | X |  |  | Corticosteroids were not used by patients |
| Monocytes (58) | Whole blood | IV: 17 | 8 | Flow cytometry |  |  | X |  | Corticosteroid were used by some patients |
| Monocytes (110) | Whole blood | IV: 26 | 15 | Flow cytometry |  |  | X |  | Corticosteroid were used by some patients |
| M2- macrophages (118) | Whole blood | 25 glioma patients | 17 | Flow cytometry | X |  |  |  |  |
| M2- macrophages (121) | Whole blood | I: 2; II: 11; III: 7; IV: 20 | 38 | Flow cytometry | X |  |  |  |  |
| M2- macrophages (122) | Whole blood | 8 glioma patients | 8 | Flow cytometry | X |  |  |  |  |
| HLA-DR-low and HLA-DR negative monocytes (6) | Whole blood | IV: 53 | 9 | Flow cytometry | X |  |  |  | Corticosteroid were used by some patients |
| HLA-DR-low and HLA-DR negative monocytes (6) | Whole blood | III: 8 | 9 | Flow cytometry | X |  |  |  | Corticosteroid were used by some patients |
| HLA-DR-low and HLA-DR-negative monocytes (119) | Whole blood | IV: 15 | 10 | Flow cytometry | X |  |  |  | Corticosteroid were used by some patients |
| HLA-DR-low and HLA-DR-negative monocytes (123) | Whole blood | IV: 10 | 20 | Flow cytometry | X |  |  |  | Corticosteroid were used by some patients |
| M1- macrophages (121) | Whole blood | I: 2; II: 11; III: 7; IV: 20 | 38 | Flow cytometry |  | X |  |  |  |
| M1- macrophages (122) | Whole blood | 8 glioma patients | 8 | Flow cytometry |  | X |  |  |  |
| Neutrophil-Lymphocyte-Ratio (41) | Whole blood | I: 81; II: 208; III: 169; IV: 292 | 682 | Hematology analyzer | X |  |  |  |  |
| Neutrophil-Lymphocyte-Ratio (42) | Whole blood | I-II: 39; III-IV: 34 | 49 | Hematology analyzer | X |  |  |  | Corticosteroid were used by some patients |
| Neutrophil-Lymphocyte-Ratio (124) | Whole blood | II-III: 39; IV: 33 | Not specified | Hematology analyzer | X |  |  |  | Corticosteroid were used by some patients |
| Neutrophil-Lymphocyte-Ratio (41) | Whole blood | IV: 292 | 682 | Hematology analyzer | X |  |  |  |  |
| Neutrophil-Lymphocyte-Ratio (102) | Whole blood | IV: 36 | 216 | Hematology analyzer | X |  |  |  | Corticosteroids were not used by patients |
| Neutrophil-Lymphocyte-Ratio (108) | Whole blood | IV: 37 | 30 | Hematology analyzer | X |  |  |  |  |
| Platelet-Lymphocyte-Ratio (41) | Whole blood | I: 81; II: 208; III: 169; IV: 292 | 682 | Hematology analyzer | X |  |  |  |  |
| Platelet-Lymphocyte-Ratio (42) | Whole blood | I-II: 39; III-IV: 34 | 49 | Hematology analyzer |  |  | X |  | Corticosteroid were used by some patients |
| Platelet-Lymphocyte-Ratio (41) | Whole blood | IV: 292 | 682 | Hematology analyzer | X |  |  |  |  |
| Platelet-Lymphocyte-Ratio (108) | Whole blood | IV: 37 | 30 | Hematology analyzer | X |  |  |  |  |
| Monocyte-Lymphocyte-Ratio (41) | Whole blood | I: 81; II: 208; III: 169; IV: 292 | 682 | Hematology analyzer | X |  |  |  |  |
| Monocyte-Lymphocyte-Ratio (102) | Whole blood | IV: 36 | 216 | Hematology analyzer |  | X |  |  | Corticosteroids were not used by patients |
| Monocyte-Lymphocyte-Ratio (41) | Whole blood | IV: 292 | 682 | Hematology analyzer | X |  |  |  |  |
| Monocyte-Lymphocyte-Ratio (102) | Whole blood | 70 glioma patients | 216 | Hematology analyzer |  | X |  |  | Corticosteroids were not used by patients |
| Total amount of dendritic cells (125) | Whole blood | I: 3; II: 6; III: 3; IV: 17 | Not specified | Flow cytometry |  | X |  |  | Corticosteroid were used by some patients |
| Myeloid/convent ional dendritic cells(mDC/CD1 1+DC/CD1c+) (6) | Whole blood | IV: 53 | 9 | Flow cytometry |  | X |  |  | Corticosteroid were used by some patients |
| Myeloid/convent ional dendritic cells(mDC/CD1 1+DC/CD1c+) (125) | Whole blood | I: 3; II: 6; III: 3; IV: 17 | Not specified | Flow cytometry |  | X |  |  | Corticosteroid were used by some patients |
| Myeloid/convent ional dendritic cells(mDC/CD1 1+DC/CD1c+) (126) | Whole blood | IV: 6 | 11 | Flow cytometry |  | X |  |  |  |
| Myeloid/convent ional dendritic cells(mDC/CD1 1+DC/CD1c+) (6) | Whole blood | III: 8 | 9 | Flow cytometry |  |  | X |  | Corticosteroid were used by some patients |
| Plasmacytoid dendritic cells(pDC/CD12 3+ DC/CD303+) (6) | Whole blood | IV: 53 | 9 | Flow cytometry |  | X |  |  | Corticosteroid were used by some patients |
| Plasmacytoid dendritic cells(pDC/CD12 3+ DC/CD303+) (125) | Whole blood | I: 3; II: 6; III: 3; IV: 17 | Not specified | Flow cytometry |  | X |  |  | Corticosteroid were used by some patients |
| Plasmacytoid dendritic cells(pDC/CD12 3+ DC/CD303+) (126) | Whole blood | IV: 6 | 11 | Flow cytometry |  | X |  |  |  |
| Plasmacytoid dendritic cells(pDC/CD12 3+ DC/CD303+) (6) | Whole blood | III: 8 | 9 | Flow cytometry |  |  | X |  | Corticosteroid were used by some patients |
| Immature dendritic cells (HLA-DRhigh/CD11c- /CD123-) (126) | Whole blood | IV: 6 | 11 | Flow cytometry |  | X |  |  |  |
| Total myeloid derived suppressor cells (26) | Whole blood | II: 8, III: 13, IV:20 | 17 | Flow cytometry | X |  |  |  | Corticosteroid were used by some patients |
| Total myeloid derived suppressor cells (127) | Whole blood | IV: 28 | 11 | Flow cytometry | X |  |  |  | Corticosteroid were used by some patients |
| Total myeloid derived suppressor cells (128) | Whole blood | I-II: 33; III: 30; IV: 60 | 57 | Flow cytometry | X |  |  |  | Corticosteroid were used by some patients |
| Total myeloid derived suppressor cells (129) | Whole blood | IV: 11 | 12 | Flow cytometry | X |  |  |  | Corticosteroid were used by some patients |
| Total myeloid derived suppressor cells (129) | Whole blood | II: 4; III: 6; | 12 | Flow cytometry |  |  | X |  | Corticosteroid were used by some patients |
| Monocytic myeloid derived suppressor cells (6) | Whole blood | IV: 8 | 9 | Flow cytometry | X |  |  |  | Corticosteroid were used by some patients |
| Monocytic myeloid derived suppressor cells (26) | Whole blood | IV: 10 | 17 | Flow cytometry | X |  |  |  | Corticosteroid were used by some patients |
| Monocytic myeloid derived suppressor cells (129) | Whole blood | IV: 11 | 12 | Flow cytometry | X |  |  |  | Corticosteroid were used by some patients |
| Monocytic myeloid derived suppressor cells (130) | Whole blood | IV: 52 | 52 | Flow cytometry | X |  |  |  | Corticosteroid were used by some patients |
| Monocytic myeloid derived suppressor cells (6) | Whole blood | III: 53 | 9 | Flow cytometry |  |  | X |  | Corticosteroid were used by some patients |
| Monocytic myeloid derived suppressor cells (26) | Whole blood | II: 5; III: 4 | 17 | Flow cytometry |  |  | X |  | Corticosteroid were used by some patients |
| Monocytic myeloid derived suppressor cells (129) | Whole blood | II: 4; III: 6; | 12 | Flow cytometry |  |  | X |  | Corticosteroid were used by some patients |
| Granylocytic/pol ymorphonuclear MDSCs (26) | Whole blood | IV: 10 | 17 | Flow cytometry | X |  |  |  | Corticosteroid were used by some patients |
| Granylocytic/pol ymorphonuclear MDSCs (127) | Whole blood | IV: 28 | 11 | Flow cytometry | X |  |  |  | Corticosteroid were used by some patients |
| Granylocytic/pol ymorphonuclear MDSCs (129) | Whole blood | IV: 11 | 12 | Flow cytometry | X |  |  |  | Corticosteroid were used by some patients |
| Granylocytic/pol ymorphonuclear MDSCs (130) | Whole blood | IV: 52 | 52 | Flow cytometry | X |  |  |  | Corticosteroid were used by some patients |
| Granylocytic/pol ymorphonuclear MDSCs (128) | Whole blood | I+II: 33; III: 30; IV: 60 | 57 | Flow cytometry |  |  | X |  | Corticosteroid were used by some patients |
| Granylocytic/pol ymorphonuclear MDSCs (129) | Whole blood | IV: 11 | 12 | Flow cytometry |  |  | X |  | Corticosteroid were used by some patients |
| Regulatory T cells (21) | Whole blood | IV: 51 | 36 | Flow cytometry | X |  |  |  | Corticosteroid were used by some patients |
| Regulatory T cells (110) | Whole blood | IV: 26 | 15 | Flow cytometry | X |  |  |  | Corticosteroid were used by some patients |
| Regulatory T cells (114) | Whole blood | IV: 8 | 6 | Flow cytometry | X |  |  |  | Corticosteroid were used by some patients |
| Regulatory T cells (131) | Whole blood | IV: 10 | 6 | Flow cytometry | X |  |  |  | Both controls and patients corticosteroids |
| Regulatory T cells (132) | Whole blood | IV: 24 | 18 | Flow cytometry | X |  |  |  |  |
| Regulatory T cells (111) | Whole blood | IV: 65 | 94 | Quantitative methylation specific PCR |  | X |  |  | Corticosteroid were used by some patients |
| Regulatory T cells (114) | Whole blood | IV: 8 | 6 | Flow cytometry |  | X |  |  | Corticosteroid were used by some patients |
| Regulatory T cells (6) | Whole blood | IV: 53 | 9 | Flow cytometry |  |  | X |  | Corticosteroid were used by some patients |
| Regulatory T cells (21) | Whole blood | IV: 51 | 36 | Flow cytometry |  |  | X |  | Corticosteroid were used by some patients |
| Regulatory T cells (115) | Whole blood | IV: 11 | 16 | Flow cytometry |  |  | X |  |  |
| Regulatory T cells (133) | Whole blood | I; 2; II: 1; III: 6; IV: 29 | 18 | Flow cytometry |  |  | X |  |  |
| Regulatory T cells (6) | Whole blood | III: 8 | 9 | Flow cytometry |  |  | X |  | Corticosteroid were used by some patients |
| Total extracellular vesicles (134) | Plasma | IV: 43 | 33 | Transmission electron microscopy | X |  |  |  |  |
| Total extracellular vesicles (135) | Plasma | Unspecified amount of glioblastoma patients, anaplastic glioma patients | Not specified | Nanoparticle tracking analysis | X |  |  |  |  |
| Total microparticles (136) | Plasma | IV: 22 | 22 controls | Flow cytometry | X |  |  |  |  |
| Total exosomes (137) | Plasma | 19 glioma patients of which some with IDH wild type tumors | 19 controls | Flow cytometry | X |  |  |  |  |
| Total protein level in exosomes (138) | Plasma | 34 glioma patients | 10 controls | Flow cytometry | X |  |  |  |  |
| Total protein level in extracellular vesicles (140) | Serum | 23 glioma patients | 12 controls | Flow cytometry |  |  | X |  |  |
| Surface proteins expression profile on extracellular vesicles (141) | Plasma | IV: 24 | 8 controls | Flow cytometry |  |  |  | Acc: >90% |  |
| EGFR expression on extracellular vesicles (140) | Serum | 23 glioma patients | 12 controls | Flow cytometry | X |  |  | AUC: 0.9 |  |

# Supplemental Table 4: Tumor grade-differentiating marker table

| Marker (reference number) | Biosource | Glial tumor grades of glioma patients in study population if specified | Biomarker detection methodology | Significantly increased in patients with higher tumor grades compared to patients with lower tumor grades | Significantly decreased in patients with higher tumor grades compared to patients with lower tumor grades | Non-significantly changed in patients with higher tumor grades compared to patients with lower tumor grades | Marker AUC, accuracy or sensitivity (SE) and specificity (SP) if measured | Medication use before blood sampling in all or some patients if reported |
| --- | --- | --- | --- | --- | --- | --- | --- | --- |
| IL-1b (2) | Serum | 32 glioma patients | ELISA | X |  |  |  |  |
| IL-6 (9) | Serum | I-II: 18; III: 25; IV: 43 | ELISA | X |  |  |  |  |
| IL-6 (18) | Plasma | II-IV: 158 | ELISA |  |  | X |  | Corticosteroid were used by some patients |
| IL-6 (13) | Serum | I-II: 18; III-IV: 20 | ELISA |  |  | X |  |  |
| IL-6 (15) | Serum | I; 2; II: 14; III:2; IV: 17 | ELISA |  |  | X |  |  |
| S100 (29) | Serum | III: 6; IV: 42 | Immunoenzyme assay | X |  |  |  |  |
| S100b (48) | Plasma | IV: 34 | ELISA |  |  | X |  |  |
| Haptoglobin (35) | Serum | II: 26; III: 49; IV: 113 | ELISA | X |  |  | AUC: 0.7 |  |
| Haptoglobin (33) | Serum | I-III: 10; IV: 8 | Radial immunodiffusion |  |  | X |  |  |
| a1 antitrypsin (33) | Serum | I-III: 10; IV: 8 | Radial immunodiffusion |  |  | X |  |  |
| A1 acid glycoprotein (33) | Serum | I-III: 10; IV: 8 | Radial immunodiffusion |  |  | X |  |  |
| Fibrinogen (142) | Serum | II: 238; III: 154; IV: 314 | Not specified | X |  |  | AUC: 0.7 |  |
| Fibrinogen (143) | Plasma | I-II: 93 ; II-IV 110 | Not specified | X |  |  |  | Corticosteroids were not used by patients |
| Fibrinogen (144) | Whole blood | I-II: 165; III-IV: 195 | Not specified | X |  |  | AUC: 0.6 | Corticosteroids were not used by patients |
| F-NLR-AGR (143) | Serum | I-II: 93 ; II-IV 110 | Not specified | X |  |  |  | Corticosteroids were not used by patients |
| Albumin (142) | Serum | II: 238; III: 154; IV: 314 | Not specified |  | X |  | AUC: 0.6 |  |
| Albumin (144) | Whole blood | I-II: 165; III-IV: 195 | Not specified |  | X |  | AUC: 0.6 | Corticosteroids were not used by patients |
| Albumin (42) | Serum | I-II: 39; III-IV: 34 | Not specified |  |  | X | AUC: 0.7 | Corticosteroid were used by some patients |
| Albumin-Globulin-Ratio (142) | Serum | II: 238; III: 154; IV: 314 | Not specified |  | X |  | AUC: 0.7 |  |
| Albumin-Globulin-Ratio (42) | Serum | I-II: 39; III-IV: 34 | Not specified |  |  | X | AUC: 0.7 | Corticosteroid were used by some patients |
| Albumin-Globulin-Ratio (143) | Serum | I-II: 93 ; II-IV 110 | Not specified |  |  | X |  | Corticosteroids were not used by patients |
| Albumin-Globulin-Ratio (166) | Whole blood | III: 14 ; IV 102 | Not specified |  |  | X |  |  |
| Prognostic-Nutritional-IndeX (144) | Whole blood | I-II: 165; III-IV: 195 | Not specified |  | X |  | AUC: 0.6 | Corticosteroids were not used by patients |
| Prognostic-Nutritional-IndeX (41) | Whole blood | I: 81; II: 208; III: 169; IV: 292 | Not specified |  | X |  | AUC: 0.7 |  |
| Prognostic-Nutritional-IndeX (42) | Serum | I-II: 39; III-IV: 34 | Not specified |  | X |  | AUC: 0.7 | Corticosteroid were used by some patients |
| Prognostic-Nutritional-IndeX (142) | Serum | II: 238; III: 154; IV: 314 | Not specified |  | X |  | AUC: 0.6 |  |
| Prognostic-Nutritional-IndeX (145) | Serum | III: 90; IV: 98 | Not specified |  | X |  |  |  |
| Prognostic-Nutritional-IndeX (146) | Serum | I-II: 122; III-IV: 227 | Not specified |  | X |  |  |  |
| Fibrinogen-albumin-score (147) | Plasma/Serum | III: 157; IV: 169 | Not specified | X |  |  |  |  |
| Plasminogen activator inhibitor (66) | Serum | I-II: 26; III-IV: 31 | ELISA |  |  |  |  | Corticosteroids were not used by patients |
| GFAP (29) | Serum | III: 6; IV: 42 | Immunoenzyme assay | X |  |  |  |  |
| GFAP (43) | Serum | III: 13; IV: 14 | ELISA | X |  |  |  | Corticosteroid were used by some patients |
| GFAP (44) | Serum | II: 17; III: 14; IV: 50 | ELISA | X |  |  |  | Corticosteroid were used by some patients |
| GFAP (45) | Serum | I-III: 4; IV: 33 | immunofluorescence assay | X |  |  |  |  |
| GFAP (46) | Serum | II-III: 39; IV: 91 | ELISA | X |  |  |  |  |
| GFAP (48) | Plasma | I-II: 7; III: 10; IV: 34 | ELISA | X |  |  |  |  |
| GFAP (148) | Plasma | Not specified | Electrochemiluminescent immunoassay | X |  |  |  |  |
| YKL-40 (50) | Serum | I-II: 41; III-IV: 197 | ELISA | X |  |  |  |  |
| YKL-40 (149) | Serum | II-III: 20; IV: 45 | ELISA | X |  |  |  |  |
| YKL-40 (51) | Plasma | II:23; III:19; IV:30 | ELISA | X |  |  |  | Corticosteroid were used by some patients |
| YKL-40 (18) | Plasma | II-IV: 158 | ELISA |  |  | X |  | Corticosteroid were used by some patients |
| VEGF (2) | Serum | 32 glioma patients | ELISA | X |  |  |  |  |
| VEGF (52) | Serum | I; 12; II:18; III:19; IV: 11 | ELISA | X |  |  |  |  |
| VEGF (53) | Serum | I-II: 7; III-IV: 23 | ELISA |  |  | X |  |  |
| VEGF (150) | Serum | I-II: 19; III-IV: 7 | ELISA |  |  | X |  |  |
| VEGF (151) | Serum | II: 76; III: 76; IV: 76 | multiplex immunoassays |  |  | X |  |  |
| MicroRNA-21 (72) | Serum | I: 2; II: 13; III: 16; IV: 69 | qRT-PCR | X |  |  | 0.8 |  |
| MicroRNA-21 (69) | Plasma | II: 10; III: 10; IV: 10 | qRT-PCR |  |  | X |  |  |
| MicroRNA-182 (81) | Serum | 6 glioma patients | Electroimmunoassay | X |  |  |  |  |
| MicroRNA-182 (82) | Serum | I-II: 24; III-IV: 30 | RT-PCR | X |  |  |  |  |
| MicroRNA-222 (72) | Serum | I: 2; II: 13; III: 16; IV: 69 | qRT-PCR | X |  |  |  |  |
| Alu methylation (89) | Serum | I-II: 38; III-IV: 71 | DNA sequencing |  | X |  |  |  |
| Alu methylation (90) | Serum | I-II: 32; III-IV: 33 | DNA sequencing |  | X |  |  |  |
| Cfdna mutations (152) | Plasma | I; 5; II: 25; III: 35; IV: 222; grade unknown: 83 | Guardant360^®^ cfDNA digital sequencing | X |  |  |  |  |
| Circulating glial tumor cells (95) | Whole blood | II: 11; III: 9; IV: 12 | Immunofluorescence | X |  |  |  |  |
| Circulating glial tumor cells (93) | Whole blood | 10 glioma patients | Immunoprecipitation |  |  | X |  |  |
| Circulating glial tumor cells (97) | Whole blood | II: 11; III: 9; IV: 11 | SE-iFISH |  |  | X |  |  |
| Blood platelets (51) | Whole blood | II:23; III:19; IV:30 | Hematology analyzer | X |  |  |  | Corticosteroid were used by some patients |
| Blood platelets (41) | Whole blood | I: 81; II: 208; III: 169; IV: 292 | Hematology analyzer |  | X |  |  |  |
| Blood platelets (144) | Whole blood | I-II: 165; III-IV: 195 | Hematology analyzer |  |  | X |  | Corticosteroids were not used by patients |
| Blood platelets (146) | Whole blood | I-II: 122; III-IV: 227 | Hematology analyzer |  |  | X |  |  |
| Blood platelets (153) | Whole blood | I-II: 53; III-IV: 100 | Hematology analyzer |  |  | X |  | Corticosteroids were not used by patients |
| Blood platelets (154) | Whole blood | I: 14; II: 81; III: 27; IV: 49 | Hematology analyzer |  |  | X |  |  |
| Blood platelets (42) | Whole blood | I-II: 39; III-IV: 34 | Hematology analyzer |  |  | X |  | Corticosteroid were used by some patients |
| Blood platelets (155) | Whole blood | II: 127; III: 61; IV: 100 | Hematology analyzer |  |  | X |  |  |
| White Blood Cells (41) | Whole blood | I: 81; II: 208; III: 169; IV: 292 | Hematology analyzer | X |  |  |  |  |
| White Blood Cells (155) | Whole blood | II: 127; III: 61; IV: 100 | Hematology analyzer | X |  |  | AUC: 0.7 |  |
| White Blood Cells (156) | Whole blood | II: 69; III: 52; IV: 139 | Hematology analyzer | X |  |  |  |  |
| White Blood Cells (42) | Whole blood | I-II: 39; III-IV: 34 | Hematology analyzer |  |  | X | AUC: 0.6 | Corticosteroid were used by some patients |
| White Blood Cells (146) | Whole blood | I-II: 122; III-IV: 227 | Hematology analyzer |  |  | X |  |  |
| White Blood Cells (157) | Whole blood | I: 8; II: 73; III: 53; IV: 105 | Hematology analyzer |  |  | X |  |  |
| Lymphocytes (41) | Whole blood | I: 81; II: 208; III: 169; IV: 292 | Hematology analyzer |  | X |  |  |  |
| Lymphocytes (42) | Whole blood | I-II: 39; III-IV: 34 | Hematology analyzer |  | X |  | AUC: 0.6 | Corticosteroid were used by some patients |
| Lymphocyte (153) | Whole blood | I-II: 53; III-IV: 100 | Hematology analyzer |  | X |  |  | Corticosteroids were not used by patients |
| Lymphocytes (154) | Whole blood | I: 14; II: 81; III: 27; IV: 49 | Hematology analyzer |  | X |  |  |  |
| CD8+ cells (20) | Whole blood | II: 16; III: 7; IV: 26 | Flow cytometry |  | X |  |  | Corticosteroids or anti-epileptic drugs were not used by patients |
| Neutrophils (41) | Whole blood | I: 81; II: 208; III: 169; IV: 292 | Hematology analyzer | X |  |  |  |  |
| Neutrophils (42) | Whole blood | I-II: 39; III-IV: 34 | Hematology analyzer | X |  |  | AUC: 0.7 | Corticosteroid were used by some patients |
| Neutrophils (108) | Whole blood |  |  | X |  |  |  |  |
| Neutrophils (144) | Whole blood | I-II: 165; III-IV: 195 | Hematology analyzer | X |  |  | AUC: 0.6 | Corticosteroids were not used by patients |
| Neutrophils (153) | Whole blood | I-II: 53; III-IV: 100 | Hematology analyzer | X |  |  |  | Corticosteroids were not used by patients |
| Neutrophils (154) | Whole blood | I: 14; II: 81; III: 27; IV: 49 | Hematology analyzer | X |  |  |  |  |
| Neutrophils (155) | Whole blood | II: 127; III: 61; IV: 100 | Hematology analyzer | X |  |  | AUC: 0.7 |  |
| Neutrophils (156) | Whole blood | II: 69; III: 52; IV: 139 | Hematology analyzer | X |  |  |  |  |
| Neutrophils (157) | Whole blood | I: 8; II: 73; III: 53; IV: 105 | Hematology analyzer | X |  |  |  |  |
| Neutrophils (158) | Whole blood | IV: 7; I; 24; III: 11; II: 7 | Hematology analyzer | X |  |  |  |  |
| Neutrophils (159) | Whole blood | III: 13; IV: 151 | Hematology analyzer |  |  | X |  | Corticosteroid were used by some patients |
| Monocytes (41) | Whole blood | I: 81; II: 208; III: 169; IV: 292 | Hematology analyzer | X |  |  |  |  |
| Monocyte (144) | Whole blood | I-II: 165; III-IV: 195 | Hematology analyzer | X |  |  | AUC: 0.6 | Corticosteroids were not used by patients |
| Monocytes (154) | Whole blood | I: 14; II: 81; III: 27; IV: 49 | Hematology analyzer | X |  |  |  |  |
| Monocytes (42) | Whole blood | I-II: 39; III-IV: 34 | Hematology analyzer |  |  | X | AUC: 0.7 | Corticosteroid were used by some patients |
| Monocytes (154) | Whole blood | I: 14; II: 81; III: 27; IV: 49 | Hematology analyzer |  |  | X |  |  |
| Monocytes (156) | Whole blood | II: 69; III: 52; IV: 139 | Hematology analyzer |  |  | X |  |  |
| Monocytes (158) | Whole blood | IV: 7; I; 24; III: 11; II: 7 | Hematology analyzer |  |  | X |  |  |
| Neutrophil-Lymphocyte-Ratio (41) | Whole blood | I: 81; II: 208; III: 169; IV: 292 | Hematology analyzer | X |  |  | AUC: 0.8 |  |
| Neutrophil-Lymphocyte-Ratio (42) | Whole blood | I-II: 39; III-IV: 34 | Hematology analyzer | X |  |  | AUC: 0.8 | Corticosteroid were used by some patients |
| Neutrophil-Lymphocyte-Ratio (161) | Whole blood | I-II: 77 ; III-IV101 | Hematology analyzer | X |  |  |  | Corticosteroids were not used by patients |
| Neutrophil-Lymphocyte-Ratio (162) | Whole blood | I-II: 57; III-IV:162 | Hematology analyzer | X |  |  |  |  |
| Neutrophil-Lymphocyte-Ratio (163) | Whole blood | I; 22; II: 71; III: 63; IV: 268 | Hematology analyzer | X |  |  |  |  |
| Neutrophil-Lymphocyte-Ratio (165) | Whole blood | I: 23; II: 36; III: 35; IV18 | Hematology analyzer | X |  |  |  | Corticosteroids were not used by patients |
| Neutrophil-Lymphocyte-Ratio (51) | Whole blood | II:23; III:19; IV:30 | Hematology analyzer | X |  |  |  | Corticosteroid were used by some patients |
| NeutrophI-Lymphocyte-Ratio (142) | Whole blood | II: 238; III: 154; IV: 314 | Hematology analyzer | X |  |  | AUC: 0.7 |  |
| Neutrophil-Lymphocyte-Ratio (143) | Whole blood | I-II: 93 ; II-IV 110 | Hematology analyzer | X |  |  |  | Corticosteroids were not used by patients |
| Neutrophil-Lymphocyte-Ratio (144) | Whole blood | I-II: 165; III-IV: 195 | Hematology analyzer | X |  |  | AUC: 0.6 | Corticosteroids were not used by patients |
| Neutrophil-Lymphocyte-Ratio (146) | Whole blood | I-II: 122; III-IV: 227 | Hematology analyzer | X |  |  |  |  |
| Neutrophil-Lymphocyte-Ratio (154) | Whole blood | I: 14; II: 81; III: 27; IV: 49 | Hematology analyzer | X |  |  | AUC: 0.7 |  |
| Neutrophil-Lymphocyte-Ratio (155) | Whole blood | II: 127; III: 61; IV: 100 | Hematology analyzer | X |  |  | AUC: 0.7 |  |
| Neutrophil-Lymphocyte-Ratio (160) | Whole blood | I-II: 59; III-IV: 124 | Hematology analyzer | X |  |  |  |  |
| Platelet-Lymphocyte-Ratio (41) | Whole blood | I: 81; II: 208; III: 169; IV: 292 | Hematology analyzer | X |  |  | AUC: 0.7 |  |
| Platelet-Lymphocyte-Ratio (142) | Whole blood | II: 238; III: 154; IV: 314 | Hematology analyzer | X |  |  | AUC: 0.6 |  |
| Platelet-Lymphocyte-Ratio (154) | Whole blood | I: 14; II: 81; III: 27; IV: 49 | Hematology analyzer | X |  |  | AUC: 0.6 |  |
| Platelet-Lymphocyte-Ratio (160) | Whole blood | I-II: 59; III-IV: 124 | Hematology analyzer | X |  |  |  |  |
| Platelet-Lymphocyte-Ratio (42) | Whole blood | I-II: 39; III-IV: 34 | Hematology analyzer |  |  | X | AUC: 0.7 | Corticosteroid were used by some patients |
| Platelet-Lymphocyte-Ratio (155) | Whole blood | II: 127; III: 61; IV: 100 | Hematology analyzer |  |  | X |  |  |
| Platelet-Lymphocyte-Ratio (162) | Whole blood | I-II: 57; III-IV:162 | Hematology analyzer |  |  | X |  |  |
| Platelet-Lymphocyte-Ratio (165) | Whole blood | I: 23; II: 36; III: 35; IV18 | Hematology analyzer |  |  | X |  | Corticosteroids were not used by patients |
| Monocyte-Lymphocyte-Ratio (41) | Whole blood | I: 81; II: 208; III: 169; IV: 292 | Hematology analyzer | X |  |  | AUC: 0.7 |  |
| Monocyte-Lymphocyte-Ratio (144) | Whole blood | I-II: 165; III-IV: 195 | Hematology analyzer | X |  |  | AUC: 0.6 | Corticosteroids were not used by patients |
| Monocyte-Lymphocyte-Ratio (154) | Whole blood | I: 14; II: 81; III: 27; IV: 49 | Hematology analyzer | X |  |  | AUC: 0.6 |  |
| Monocyte-Lymphocyte-Ratio (160) | Whole blood | I-II: 59; III-IV: 124 | Hematology analyzer |  |  | X |  |  |
| Monocyte-Lymphocyte-Ratio (162) | Whole blood | I-II: 57; III-IV:162 | Hematology analyzer |  |  | X |  |  |
| Systemic immune-inflammation indeX (144) | Whole blood | I-II: 165; III-IV: 195 | Hematology analyzer | X |  |  | AUC: 0.6 | Corticosteroids were not used by patients |
| Systemic immune-inflammation indeX (146) | Whole blood | I-II: 122; III-IV: 227 | Hematology analyzer | X |  |  |  |  |
| Systemic immune-inflammation indeX (153) | Whole blood | I-II: 53; III-IV: 100 | Hematology analyzer | X |  |  | SE: 75% ; SP: 66% ; AUC: 0.8 | Corticosteroids were not used by patients |
| Systemic immune-inflammation indeX (166) | Whole blood | III: 14 ; IV 102 | Hematology analyzer | X |  |  |  |  |
| Total dendritic cells (125) | Whole blood | I: 3; II: 6; III: 3; IV: 17 | Flow cytometry |  | X |  |  | Corticosteroid were used by some patients |
| Myeloid/conventional dendritic cells  (mDC/CD11+DC  /CD1c+) (125) | Whole blood | I: 3; II: 6; III: 3; IV: 17 | Flow cytometry |  | X |  |  | Corticosteroid were used by some patients |
| Plasmacytoid dendritic cells  (pDC/CD123+DC  /CD303+) (125) | Whole blood | I: 3; II: 6; III: 3; IV: 17 | Flow cytometry |  | X |  |  | Corticosteroid were used by some patients |
| EGFR+ EVs (140) | Serum | I-II: 4 ; III-IV 13 | Flow cytometry | X |  |  |  |  |
| Glucose (167) | Serum | II: 65; III: 38; IV: 72 | Not specified | X |  |  |  |  |
| Lactate (168) | Serum | I-II: 54 ; III-IV 20 |  | X |  |  | 0.7 | Corticosteroid were used by some patients |
| Lactate (169) | Plasma | I-II: 67 ; III-IV 95 |  | X |  |  |  |  |
| Total protein level in exosomes (138) | Plasma | III: 5; IV: 6 | Flow cytometry | X |  |  |  |  |
| EGFR expression on extracellular vesicles (140) | Serum | I-II: 4; III-IV: 13 | Flow cytometry | X |  |  |  |  |

# Supplemental Table 5: Table for differentiating markers of glial tumors compared to other intracranial pathologies

| Marker (reference number) | Biosource | Glial tumor grades of glioma patients in study population if specified | Control population | Biomarker detection methodology | Marker significantly increased in glioma patients compared to patients with other brain tumor types | Marker significantly decreased in glioma or glioblastoma patients compared to patients with other brain tumor types | Marker non-significantly changed in glioma or glioblastoma patients compared to patients with other brain tumor types | Marker AUC, accuracy or sensitivity (SE) and specificity (SP) if measured | Medication use before blood sampling in all or some patients if reported |
| --- | --- | --- | --- | --- | --- | --- | --- | --- | --- |
| IL-6 (13) | Serum | I-II: 18; III-IV: 20 | 24 meningioma patients  18 schwannoma patients | ELISA |  |  | X |  |  |
| S100b (48) | Plasma | II: 7; III: 10; IV: 34 | 41 cerebral metastasis patients  13 meningioma patients | ELISA |  |  | X |  |  |
| Albumin (42) | Serum | I-II: 39; III-IV: 34 | 20 meningioma patients | Not specified | X |  |  |  | Corticosteroid were used by some patients |
| Albumin (41) | Serum | I: 81; II: 208; III: 169; IV: 292 | 271 meningioma patients  44 neuroma patients  102 epilepsy patients | Not specified |  | X |  |  |  |
| Albumin-Globulin-Ratio (42) | Serum | I-II: 39; III-IV: 34 | 20 meningioma patients | Not specified |  |  | X |  | Corticosteroid were used by some patients |
| Prognostic-Nutritional-index (42) | Serum | I-II: 39; III-IV: 34 | 20 meningioma patients | Not specified | X |  |  |  | Corticosteroid were used by some patients |
| Prognostic-Nutritional-index (41) | Serum | I: 81; II: 208; III: 169; IV: 292 | 271 meningioma patients  44 neuroma patients  102 epilepsy patients | Not specified |  | x |  |  |  |
| GFAP (29) | Serum | IV: 42 | 73 cerebral metastasis patients | Immunoenzyme assay | X |  |  |  |  |
| GFAP (44) | Serum | IV: 50 | 17 cerebral metastasis patients | ELISA | X |  |  |  | Corticosteroid were used by some patients |
| GFAP (46) | Serum | IV: 91 | 114 cerebral metastasis patients | ELISA | X |  |  |  |  |
| GFAP (171) | Serum | IV: 25 | 7 brain metastasis patients |  | X |  |  |  |  |
| GFAP (48) | Plasma | IV: 34 | 13 meningioma patients  41 cerebral metastases patients | ELISA | X |  |  |  |  |
| GFAP (236) | Serum | IV: 91 | 78 non-tumor CNS diseases such as multiple sclerosis, Parkinson’s disease and others | ELISA | X |  |  | SE: 27% or 85%  SP: 99% or 70% |  |
| GFAP (236) | Serum | IV: 33 | 80 patients with other intracranial tumors | ELISA |  |  |  |  |  |
| YKL-40 (47) | Plasma | IV: 111 | 40 nonglial brain tumors | ELISA |  |  | X |  |  |
| VEGF (56) | Serum | IV: 12 | 10 patients with cerebral metastases | ELISA |  | X |  |  |  |
| VEGF (172) | Plasma | IV: 22 | 28 patients with cerebral metastases | ELISA |  | X |  |  |  |
| VEGF (172) | Plasma | IV: 22 | 12 astrocytoma patients  16 meningioma patients | ELISA |  |  | X |  |  |
| VEGF (172) | Plasma | 12 astrocytoma patients | 28 patients with cerebral metastases | ELISA |  |  | X |  |  |
| VEGF (53) | Serum | III-IV: 23 | 6 patients with cerebral metastasis patients  9 patients with meningiomas  3 other brain tumor types | ELISA |  |  | X |  |  |
| MicroRNA-21 (69) | Plasma | II: 10; III: 10; IV: 10 | 10 meningioma patients  10 pituitary adenoma patients | qRT-PCR |  | X |  |  |  |
| MicroRNA-21 (76) | Plasma | IV: 25 | 25 primary central nervous system lymphoma patients | qRT-PCR |  | X |  | 0.9 |  |
| MicroRNA-21 (72) | Serum | I: 2; II: 13; III: 16; IV: 69 | 11 patients with cerebral metastases | qRT-PCR |  |  | X |  |  |
| MicroRNA-222 (72) | Serum | I: 2; II: 13; III: 16; IV: 69 | 11 patients with cerebral metastases | qRT-PCR | X |  |  |  |  |
| Total cfDNA (86) | Serum | IV: 122 | 55 cerebral metastasis | Fluorimetry | X |  |  |  |  |
| cfDNA methylome (88) | Plasma | 70 IDH-mutant glioma patients | 52 IDH-wild-type  60 meningiomas  9 hemangio-pericytoma patients  14 low-grade glial–neuronal tumors  15 cerebral metastasis patients | Illumina HumanBeadChip 850K array |  |  |  | AUC: 0.8 |  |
| cfDNA methylome (88) | Plasma | 52 IDH wild-type glioma patients | 70 IDH mutant gliomas  60 meningiomas  9 hemangio-pericytoma patients  14 low-grade glial–neuronal tumors  15 cerebral metastasis patients | Illumina HumanBeadChip 850K array |  |  |  | AUC: 0.7 |  |
| Blood platelets (41) | Whole blood | I: 81; II: 208; III: 169; IV: 292 | 271 meningioma patients  44 neuroma patients  102 epilepsy patients | Hematology analyzer |  |  | X |  |  |
| Blood platelets (108) | Whole blood | IV: 37 | 39 epilepsy patients  32 cerebral metastasis patients  32 meningioma patients | Hematology analyzer |  |  | X |  |  |
| White blood cells (173) | Whole blood | II: 6; III: 8; IV: 36 | 50 meningioma | Hematology analyzer | X |  |  |  |  |
| White blood cells (41) | Whole blood | I: 81; II: 208; III: 169; IV: 292 | 271 meningioma patients  44 neuroma patients  102 epilepsy patients | Hematology analyzer | X |  |  |  |  |
| White blood cells (42) | Whole blood | I-II: 39; III-IV: 34 | 20 meningioma patients | Hematology analyzer |  |  | X |  | Corticosteroid were used by some patients |
| Lymphocytes (41) | Whole blood | I: 81; II: 208; III: 169; IV: 292 | 271 meningioma patients | Hematology analyzer | X |  |  |  |  |
| Lymphocytes (41) | Whole blood | I: 81; II: 208; III: 169; IV: 292 | 44 neuroma patients | Hematology analyzer |  | X |  |  |  |
| Lymphocytes (41) | Whole blood | I: 81; II: 208; III: 169; IV: 292 | 102 epilepsy patients | Hematology analyzer |  |  | X |  |  |
| Lymphocytes (108) | Whole blood | IV: 37 | 39 epilepsy | Hematology analyzer |  | X |  |  |  |
| Lymphocytes (108) | Whole blood | IV: 37 | 32 cerebral metastasis patients  32 meningioma patients | Hematology analyzer |  |  | X |  |  |
| Lymphocytes (174) | Whole blood | IV: 80 | 70 cerebral metastasis patients | Hematology analyzer | X |  |  |  |  |
| Lymphocytes (42) | Whole blood | I-II: 39; III-IV: 34 | 20 meningioma patients | Hematology analyzer |  |  | X |  | Corticosteroid were used by some patients |
| Neutrophils (41) | Whole blood | I: 81; II: 208; III: 169; IV: 292 | 271 meningioma patients  44 neuroma patients  102 epilepsy patients | Hematology analyzer | X |  |  |  |  |
| Neutrophils (173) | Whole blood | II: 6; III: 8; IV: 36 | 50 meningioma patients | Hematology analyzer | X |  |  |  |  |
| Neutrophils (108) | Whole blood | IV: 37 | 39 epilepsy patients | Hematology analyzer | X |  |  |  |  |
| Neutrophils (42) | Whole blood | I-II: 39; III-IV: 34 | 20 meningioma patients | Hematology analyzer |  |  | X |  |  |
| Neutrophils (108) | Whole blood | IV: 37 | 32 cerebral metastasis patients, 32 meningioma | Hematology analyzer |  |  | X |  |  |
| Neutrophils (174) | Whole blood | IV: 80 | 70 cerebral metastases | Hematology analyzer |  |  | X |  |  |
| Monocytes (41) | Whole blood | I: 81; II: 208; III: 169; IV: 292 | 271 meningioma patients  44 neuroma patients  102 epilepsy patients | Hematology analyzer | X |  |  |  |  |
| Neutrophil-Lymphocyte-Ratio (41) | Whole blood | I: 81; II: 208; III: 169; IV: 292 | 271 meningioma patients  44 neuroma patients  102 epilepsy patients | Hematology analyzer | X |  |  |  |  |
| Neutrophil-Lymphocyte-Ratio (108) | Whole blood | IV: 37 | 39 epilepsy patients | Hematology analyzer | X |  |  |  |  |
| Neutrophil-Lymphocyte-Ratio (42) | Whole blood | I-II: 39; III-IV: 34 | 20 meningioma patients | Hematology analyzer |  |  | X |  | Corticosteroid were used by some patients |
| Neutrophil-Lymphocyte-Ratio (108) | Whole blood | IV: 37 | 32 cerebral metastasis patients  32 meningioma patients | Hematology analyzer |  |  | X |  |  |
| Neutrophil-Lymphocyte-Ratio (174) | Whole blood | IV: 80 | 70 cerebral metastasis patients | Hematology analyzer |  |  | X |  |  |
| Platelet-Lymphocyte-Ratio (41) | Whole blood | I: 81; II: 208; III: 169; IV: 292 | 102 epilepsy patients | Hematology analyzer | X |  |  |  |  |
| Platelet-Lymphocyte-Ratio (108) | Whole blood | IV: 37 | 39 epilepsy patients | Hematology analyzer | X |  |  |  |  |
| Platelet-Lymphocyte-Ratio (108) | Whole blood | IV: 37 | 32 cerebral metastases patients | Hematology analyzer |  | X |  |  |  |
| Platelet-Lymphocyte-Ratio (108) | Whole blood | IV: 37 | 32 meningioma patients | Hematology analyzer |  |  | X |  |  |
| Platelet-Lymphocyte-Ratio (174) | Whole blood | IV: 80 | 70 cerebral metastases patients | Hematology analyzer |  | X |  |  |  |
| Platelet-Lymphocyte-Ratio (41) | Whole blood | I: 81; II: 208; III: 169; IV: 292 | 271 meningioma patients  44 neuroma patients | Hematology analyzer |  |  | X |  |  |
| Platelet-Lymphocyte-Ratio (42) | Whole blood | I-II: 39; III-IV: 34 | 20 meningioma patients | Hematology analyzer |  |  | X |  |  |
| Monocyte-Lymphocyte-Ratio (174) | Whole blood | IV: 80 | 70 cerebral metastases patients | Hematology analyzer |  | X |  |  |  |
| Monocyte-Lymphocyte-Ratio (41) | Whole blood | I: 81; II: 208; III: 169; IV: 292 | 271 meningioma patients  44 neuroma patients  102 epilepsy patients | Hematology analyzer | X |  |  |  |  |
| Myeloid derived suppressor cells (123) | Whole blood | IV: 10 | 5 meningioma patients  6 pituitary adenoma patients | Hematology analyzer | X |  |  |  |  |
| Extracellular vesicles (134) | Plasma | IV: 13 | 13 patients with brain metastases | Hematology analyzer | X |  |  |  |  |

# Supplemental Table 6: Prognostic marker table

| Marker (reference number) | Biosource | Glial tumor grades of glioma patients in study population if specified | Biomarker detection methodology | Marker significantly increased at baseline or increase during treatment in glioma or glioblastoma patients with worse survival | Marker significantly decreased at baseline or decrease during treatment in glioma or glioblastoma patients with worse survival | Marker non-significantly changed in glioma or glioblastoma patients with long compared to short survival | Hazard ratio (HR) if measured | Medication use before blood sampling in all or some patients if reported |
| --- | --- | --- | --- | --- | --- | --- | --- | --- |
| IL-6 (9) | Serum | I-II: 18; III: 25; IV: 43 | ELISA | X |  |  |  |  |
| IL-6 (175) | Serum | I-II: 21; III-IV: 48 | Radioimmunoassay method | X |  |  | 2.6 for 60 month mortality and 4.0 for 12 month mortality in glioma patients |  |
| IL-6 (176) | Plasma |  |  | X |  |  |  | Corticosteroid were used by some patients |
| IL-6 (18) | Plasma | II-IV: 158 | ELISA |  |  | X |  | Corticosteroid were used by some patients |
| IL-6 (177) | Plasma | II-IV 40 | ELISA |  |  | X |  |  |
| IL-6 (10) | Serum | IV: 47 | ELISA |  |  | X |  | Corticosteroid were used by some patients |
| IL-6 (16) | Serum | III-IV: 44 | ELISA |  |  | X |  |  |
| IL-6 (31) | Serum | IV: 14 | ELISA |  |  | X |  |  |
| IL-10 (6) | Serum | III: 8; IV: 53 | Immunoassay |  |  | X |  | Corticosteroid were used by some patients |
| S100b (178) | Serum | II-IV 20 | immunoluminometric assay | X |  |  |  |  |
| S100b (22) | Serum | IV: 125 | ELISA | X |  |  |  |  |
| S100b (179) | Serum | III: 1; IV: 85 | Immunoassay |  |  | X |  | Corticosteroid were used by some patients |
| S100b (179) | Serum | 27 glioma patients | Immunoassay | X |  |  |  | Corticosteroid were used by some patients |
| sTNF-R1 (32) | Plasma | IV: 112 | ELISA | X |  |  |  | Corticosteroid were used by some patients |
| Fibrinogen (142) | Plasma | II: 238; III: 154; IV: 314 | Not specified | X |  |  |  |  |
| Fibrinogen (143) | Plasma | I-II: 93 ; II-IV 110 | Not specified | X |  |  |  | Corticosteroids were not used by patients |
| Fibrinogen (142) | Plasma | IV: 314 | Not specified | X |  |  |  |  |
| Fibrinogen (180) | Plasma | IV 187 | Not specified | X |  |  | 1.5 | Corticosteroids were not used by patients |
| Fibrinogen (10) | Plasma | IV: 47 | ELISA |  |  | X |  | Corticosteroid were used by some patients |
| Fibrinogen albumin score (147) | Serum | III: 157; IV: 169 | Not specified | X |  |  | 1-3.03 depending on FA score |  |
| Fibrinogen-NLR score (180) | Plasma | IV 187 | Not specified | X |  |  | 1-2.78 depending on F-NLR-score | Corticosteroids were not used by patients |
| F-NLR-AGR (143) | Serum | I-II: 93 ; II-IV 110 | Not specified | X |  |  | 1-3.8 depending on F-NLR-AGR score | Corticosteroids were not used by patients |
| CRP (37) | Serum | IV: 28 | Antibody microarray | X |  |  | 1.0 |  |
| CRP (181) | Serum | IV 565 | Not specified | X |  |  |  | Corticosteroid were used by some patients |
| CRP (182) | Serum | I-II: 23; III-IV: 142 | Turbidimetry | X |  |  |  | Corticosteroid were used by some patients |
| CRP (10) | Plasma | IV: 47 | ELISA |  |  | X |  | Corticosteroid were used by some patients |
| CRP (38) | Plasma | IV: 14 | Quantitative targeted absolute proteomics |  |  | X |  |  |
| CRP (183) | Plasma | IV 497 | Not specified |  |  | X |  |  |
| CRP (184) | Serum | II: 20; III: 53; IV: 86 | Not specified |  |  | X |  | Corticosteroids were not used by patients |
| CRP/albumin (185) | Serum | IV: 153 | Not specified | X |  |  | 2.4 | Corticosteroid were used by some patients |
| Albumin (186) | Serum | IV: 214 | Not specified |  | X |  | 1.0 | Corticosteroid were used by some patients |
| Albumin (187) | Serum | IV: 24 | Not specified |  | X |  |  | Corticosteroid were used by some patients |
| Albumin (188) | Serum | IV: 685 | Not specified |  | X |  |  |  |
| Albumin (142) | Serum | II: 238; III: 154; IV: 314 | Not specified |  | X |  |  |  |
| Albumin (142) | Serum | IV: 314 | Not specified |  | X |  |  |  |
| Albumin (189) | Serum | IV: 166 | Not specified |  |  | X |  |  |
| Albumin (190) | Serum | IV: 84 | Not specified |  |  | X |  |  |
| Albumin (191) | Serum | IV: 282 | Not specified |  |  | X |  |  |
| Albumin (166) | Serum | III: 14; IV: 102 | Not specified |  |  | X |  |  |
| Complement C9 (38) | Plasma | IV: 14 | Quantitative targeted absolute proteomics |  |  | X |  |  |
| Albumin/globulin ratio (142) | Serum | II: 238; III: 154; IV: 314 | Not specified |  | X |  |  |  |
| Albumin/globulin ratio (142) | Serum | IV: 314 | Not specified |  | X |  |  |  |
| Albumin/globulin ratio (143) | Serum | I-II: 93; II-IV: 110 | Not specified |  | X |  |  | Corticosteroids were not used by patients |
| Albumin/globulin ratio (166) | Serum | III: 14 ; IV: 102 | Not specified |  | X |  | 0.6 |  |
| Albumin/globulin ratio (192) | Serum | II-III: 404; IV: 188 | Not specified |  |  | X |  |  |
| Progostic nutritional index (142) | Serum | II: 238; III: 154; IV: 314 | Not specified |  | X |  |  |  |
| Progostic nutritional index (142) | Serum | IV: 314 | Not specified |  | X |  |  |  |
| Prognostic nutritional index (147) | Serum | III: 157; IV: 169 | Not specified |  | X |  |  |  |
| Prognostic nutritional index (190) | Serum | IV: 84 | Not specified |  | X |  | 0.5 |  |
| Prognostic nutritional index (191) | Serum | IV: 282 | Not specified |  |  | X |  |  |
| Progostic nutritional index (193) | Serum | IV: 300 | Not specified |  |  | x |  |  |
| Sanbo scoring system (193) | Serum | IV: 300 | Not specified |  | X |  | 1-0.7 depending on SSS-score 194 |  |
| Complement factor 4 (195) | Serum | IV: 26 | Not specified |  |  | X |  | Corticosteroid were used by some patients |
| GFAP (43) | Serum | III-IV: 14 | ELISA | X |  |  | 5.9 | Corticosteroid were used by some patients |
| GFAP (46) | Serum | IV: 91 | ELISA | X |  |  |  |  |
| GFAP (45) | Serum | IV: 33 | Immunofluorescence assay |  |  | X |  |  |
| YKL-40 (50) | Serum | I-II: 41; III-IV: 197 | ELISA | X |  |  | 1.4 |  |
| YKL-40 (196) | Serum | III: 66 ; IV 77 | ELISA | X |  |  | 1.4 in glioblastoma and 2.2 in anaplastic glioma |  |
| YKL-40 (51) | Plasma | II:23; III:19; IV:30 | ELISA | X |  |  | 1.0 | Corticosteroid were used by some patients |
| YKL-40 (18) | Plasma | II-IV: 158 | ELISA |  |  | X | 2.1 | Corticosteroid were used by some patients |
| YKL-40 (197) | Serum | IV 60 | Immunoassay | X |  |  | 2.0 |  |
| YKL-40 (18) | Plasma | IV 94 | ELISA | X |  |  |  | Corticosteroid were used by some patients |
| YKL-40 (198) | Serum | IV 55 | ELISA |  |  | X |  | Corticosteroid were used by some patients |
| VEGF (19) | Plasma | IV 20 | Immunoassay | X |  |  | 3.1 |  |
| VEGF (199) | Plasma | II:1 ; III:5 ; IV 20 | ELISA | X |  |  | 3.2 | Corticosteroid were used by some patients |
| VEGF (10) | Serum | IV: 47 | ELISA |  |  | X |  | Corticosteroid were used by some patients |
| VEGF (31) | Serum | IV: 14 | ELISA |  |  | X |  |  |
| VEGF (16) | Serum | III-IV: 44 | ELISA |  |  | X |  |  |
| VEGF (150) | Serum | I-II: 19; III-IV: 7 | ELISA |  |  | X |  |  |
| Von Willebrand factor antigen (200) | Plasma | IV: 57 | Immunoturbimetric assay | X |  |  | 5.8 |  |
| D-dimer (201) | Plasma | 49 glioma | ELISA | X |  |  | 2.3 | Corticosteroids |
| D-dimer (202) | Plasma | IV 23 | quantitative latex assay | X |  |  | 10.8 | Corticosteroids |
| Prothrombin fragments 1+2 (201) | Plasma | 49 glioma | ELISA | X |  |  | 2.9 | Corticosteroids |
| Plasminogen activator inhibitor (66) | Serum | I-II: 26; III-IV: 31 | ELISA | X |  |  |  | Corticosteroids were not used by patients |
| aPTT (203) | Plasma | IV: 153 | Not specified | X |  |  | 1.1 |  |
| Prothrombin time (203) | Plasma | IV: 153 | Not specified | X |  |  | 0.9 |  |
| MicroRNA-21 (74) | Serum | IV: 20 | qRT-PCR | X |  |  |  |  |
| MicroRNA-182 (204) | Serum | IV: 106 | nCounter Human v2 miRNA Expression Assay (NanoString Technologies) | X |  |  |  |  |
| MicroRNA-182 (83) | Plasma | I: 18; II: 23; III: 32; IV: 39 | qRT-PCR | X |  |  | 0.6 |  |
| MicroRNA-222 (204) | Serum | IV: 106 | nCounter Human v2 miRNA Expression Assay (NanoString Technologies) |  |  | X |  |  |
| MicroRNA-222 (205) | Serum | IV: 20 | qRT-PCR | X |  |  |  |  |
| MicroRNA-222 (85) | Plasma | 50 glioma patients | qRT-PCR | X |  |  |  |  |
| Total cfDNA (87) | Plasma | IV: 42 | qRT-PCR | X |  |  |  |  |
| Alu methylation (89) | Serum | I-II: 38; III-IV: 71 | DNA sequencing |  | X |  |  |  |
| Alu methylation (90) | Serum | I-II: 32; III-IV: 33 | DNA sequencing |  | X |  |  |  |
| cfDNA mutation (207) | Plasma | IV: 5 | DNA sequencing |  |  | X |  |  |
| Circulating glial tumor cells (100) | Whole blood | IV: 13 | Immuno-precipitation | X |  |  |  |  |
| Blood platelets (203) | Whole blood | IV: 153 | Hematology analyzer | X |  |  | 1.6 |  |
| Blood platelets (51) | Whole blood | II:23; III:19; IV:30 | Hematology analyzer |  |  | X |  | Corticosteroid were used by some patients |
| Blood platelets (155) | Whole blood | II: 127; III: 61; IV: 100 | Hematology analyzer |  |  | X |  |  |
| Blood platelets (161) | Whole blood | I-II: 77 ; III-IV: 101 | Hematology analyzer |  |  | X |  | Corticosteroids were not used by patients |
| Blood platelets (166) | Whole blood | III: 14 ; IV: 102 | Hematology analyzer |  |  | X |  |  |
| Blood platelets (183) | Whole blood | IV: 497 | Hematology analyzer |  |  | X |  |  |
| Blood platelet (190) | Whole blood | IV: 84 | Hematology analyzer |  |  | X |  |  |
| Blood platelets (208) | Whole blood | IV: 84 | Hematology analyzer |  |  | X |  |  |
| Blood platelets (209) | Whole blood | IV: 107 | Hematology analyzer |  |  | X |  | Corticosteroid were used by some patients |
| Blood platelets (210) | Whole blood | IV: 152 | Hematology analyzer |  |  | X |  | Corticosteroids were not used by patients |
| Blood platelets (211) | Whole blood | IV: 80 | Hematology analyzer |  |  | X |  | Corticosteroid were used by some patients |
| Blood platelet (212) | Whole blood | IV: 140 | Hematology analyzer |  |  | X |  |  |
| White Blood Cells (155) | Whole blood | II: 127; III: 61; IV: 100 | Hematology analyzer | X |  |  |  |  |
| White Blood Cells (21) | Whole blood | IV: 51 | Hematology analyzer | X |  |  | 3.0 | Corticosteroid were used by some patients |
| White Blood Cell (181) | Whole blood | IV: 565 | Hematology analyzer | X |  |  |  | Corticosteroid were used by some patients |
| White Blood Cells (159) | Whole blood | III: 13; IV: 151 | Hematology analyzer |  |  | X |  | Corticosteroid were used by some patients |
| White Blood Cells (183) | Whole blood | IV: 497 | Hematology analyzer |  |  | X |  |  |
| White Blood Cells (190) | Whole blood | IV: 84 | Hematology analyzer |  |  | X |  |  |
| Lymphocytes (213) | Whole blood | III: 22; IV: 113 | Hematology analyzer |  | X |  | 0.6 |  |
| Lymphocytes (155) | Whole blood | II: 127; III: 61; IV: 100 | Hematology analyzer |  |  | X |  |  |
| Lymphocytes (159) | Whole blood | III: 13; IV: 151 | Hematology analyzer |  |  | X |  | Corticosteroid were used by some patients |
| Lymphocytes (183) | Whole blood | IV: 497 | Hematology analyzer |  |  | X |  |  |
| Lymphocytes (190) | Whole blood | IV: 84 | Hematology analyzer |  |  | X |  |  |
| Lymphocytes (217) | Whole blood | IV: 122 | Hematology analyzer |  |  | X |  |  |
| Lymphocytes (191) | Whole blood | IV: 282 | Hematology analyzer |  |  | X |  |  |
| Lymphocytes (195) | Whole blood | IV: 26 | Hematology analyzer |  |  | X |  | Corticosteroid were used by some patients |
| Lymphocytes (208) | Whole blood | IV: 84 | Hematology analyzer |  |  | X |  |  |
| Lymphocytes (210) | Whole blood | IV: 152 | Hematology analyzer |  |  | X |  | Corticosteroids were not used by patients |
| Lymphocytes (211) | Whole blood | IV: 80 | Hematology analyzer |  |  | X |  | Corticosteroid were used by some patients |
| Lymphocytes (212) | Whole blood | IV: 140 | Hematology analyzer |  |  | X |  |  |
| Lymphocytes (215) | Whole blood | IV: 84 | Hematology analyzer |  |  | X |  | Corticosteroids were not used by patients |
| Lymphocytes (216) | Whole blood | IV: 385 | Hematology analyzer |  |  | X |  |  |
| Lymphocytes (166) | Whole blood | III: 14; IV: 102 | Hematology analyzer |  |  | X |  |  |
| Lymphocytes (183) | Whole blood | IV: 497 | Hematology analyzer |  |  | X |  |  |
| CD3+ cells (195) | Whole blood | IV: 26 | Flow cytometry |  |  | X |  | Corticosteroid were used by some patients |
| CD+4-cells (125) | Whole blood | I: 3; II: 6; III: 3; IV: 17 | Flow cytometry |  | X |  |  | Corticosteroid were used by some patients |
| CD+4-cells (195) | Whole blood | IV: 26 | Flow cytometry |  | X |  |  | Corticosteroid were used by some patients |
| NK-cells (21) | Whole blood | IV: 51 | Flow cytometry |  | X |  |  | Corticosteroid were used by some patients |
| NK-cells (118) | Whole blood | 25 glioma patients | Flow cytometry |  |  | X |  |  |
| CD8+ cells (21) | Whole blood | IV: 51 | Flow cytometry |  | X |  |  | Corticosteroid were used by some patients |
| CD8+ cells (195) | Whole blood | IV: 26 | Flow cytometry |  |  | X |  | Corticosteroid were used by some patients |
| Neutrophils (155) | Whole blood | II: 127; III: 61; IV: 100 | Hematology analyzer | X |  |  |  |  |
| Neutrophils (159) | Whole blood | III: 13; IV:151 | Hematology analyzer | X |  |  | 1.6 | Corticosteroid were used by some patients |
| Neutrophils (216) | Whole blood | IV: 385 | Hematology analyzer | X |  |  | 1.6 |  |
| Neutrophils (166) | Whole blood | III: 14; IV: 102 | Hematology analyzer |  |  | X |  |  |
| Neutrophils (213) | Whole blood | III: 22; IV: 113 | Hematology analyzer |  |  | X |  |  |
| Neutrophils (183) | Whole blood | IV 497 | Hematology analyzer |  |  | X |  |  |
| Neutrophils (190) | Whole blood | IV: 84 | Hematology analyzer |  |  | X |  |  |
| Neutrophils (210) | Whole blood | IV: 152 | Hematology analyzer |  |  | X |  | Corticosteroids were not used by patients |
| Neutrophils (211) | Whole blood | IV: 80 | Hematology analyzer |  |  | X |  | Corticosteroid were used by some patients |
| Neutrophils (212) | Whole blood | IV: 140 | Hematology analyzer |  |  | X |  |  |
| Neutrophils (215) | Whole blood | IV: 84 | Hematology analyzer |  |  | X |  | Corticosteroids were not used by patients |
| Monocytes (183) | Whole blood | IV: 497 | Hematology analyzer |  |  | X |  |  |
| Monocytes (190) | Whole blood | IV: 84 | Hematology analyzer |  |  | X |  |  |
| Monocytes (216) | Whole blood | IV: 385 | Hematology analyzer |  |  | X |  |  |
| Monocytes (159) | Whole blood | III: 13; IV: 151 | Hematology analyzer |  |  | X |  | Corticosteroid were used by some patients |
| Neutrophil-Lymphocyte -Ratio (51) | Whole blood | II:23; III:19; IV:30 | Hematology analyzer | X |  |  | 1.2 | Corticosteroid were used by some patients |
| Neutrophil-Lymphocyte -Ratio (142) | Whole blood | II: 238; III: 154; IV: 314 | Hematology analyzer | X |  |  |  |  |
| Neutrophil-Lymphocyte -Ratio (142) | Whole blood | IV: 314 | Hematology analyzer | X |  |  |  |  |
| Neutrophil-Lymphocyte -Ratio (143) | Whole blood | I-II: 93 ; II-IV 110 | Hematology analyzer | X |  |  |  | Corticosteroids were not used by patients |
| Neutrophil-Lymphocyte -Ratio (220) | Whole blood | III-IV: 135 | Hematology analyzer |  |  | X |  |  |
| Neutrophil-Lymphocyte -Ratio (155) | Whole blood | II: 127; III: 61; IV: 100 | Hematology analyzer | X |  |  |  |  |
| Neutrophil-Lymphocyte -Ratio (156) | Whole blood | II: 69; III: 52; IV: 139 | Hematology analyzer | X |  |  |  |  |
| Neutrophil-Lymphocyte -Ratio (162) | Whole blood | I-II: 57; III-IV:162 | Hematology analyzer | X |  |  | 1.8 |  |
| Neutrophil-Lymphocyte -Ratio (165) | Whole blood | I: 23; II: 36; III: 35; IV18 | Hematology analyzer | X |  |  | 1.9 | Corticosteroids were not used by patients |
| Neutrophil-Lymphocyte -Ratio (184) | Whole blood | II: 20; III: 53; IV: 86 | Hematology analyzer | X |  |  | 1.8 | Corticosteroids were not used by patients |
| Neutrophil-Lymphocyte -Ratio (192) | Whole blood | I-II: 358 | Hematology analyzer | X |  |  | 1.5 |  |
| Neutrophil-Lymphocyte -Ratio (192) | Whole blood | III-IV: 170 | Hematology analyzer |  |  |  | 2.2 |  |
| Neutrophil-Lymphocyte -Ratio (213) | Whole blood | III: 22; IV: 113 | Hematology analyzer | X |  |  | 1.7 |  |
| Neutrophil-Lymphocyte -Ratio (219) | Whole blood | II: 226 | Hematology analyzer | X |  |  | 1.2 |  |
| Neutrophil-Lymphocyte -Ratio (124) | Whole blood | II-III: 39; IV: 33 | Hematology analyzer | X |  |  | 2.0 | Corticosteroid were used by some patients |
| Neutrophil-Lymphocyte -Ratio (157) | Whole blood | IV 105 | Hematology analyzer | X |  |  | 2.0 |  |
| Neutrophil-Lymphocyte -Ratio (180) | Whole blood | IV: 187 | Hematology analyzer | X |  |  | 1.8 | Corticosteroids were not used by patients |
| Neutrophil-Lymphocyte -Ratio (209) | Whole blood | IV: 107 | Hematology analyzer | X |  |  |  | Corticosteroid were used by some patients |
| Neutrophil-Lymphocyte -Ratio (210) | Whole blood | IV: 152 | Hematology analyzer | X |  |  | 1.1 | Corticosteroids were not used by patients |
| Neutrophil-Lymphocyte -Ratio (215) | Whole blood | IV: 84 | Hematology analyzer | X |  |  | 1.6 | Corticosteroids were not used by patients |
| Neutrophil-Lymphocyte -Ratio (221) | Whole blood | IV: 192 | Hematology analyzer | X |  |  |  |  |
| Neutrophil-Lymphocyte -Ratio (222) | Whole blood | IV: 166 | Hematology analyzer | X |  |  | 1.7 | Corticosteroids were not used by patients |
| Neutrophil-Lymphocyte -Ratio (223) | Whole blood | IV: 51 | Hematology analyzer | X |  |  |  |  |
| Neutrophil-Lymphocyte -Ratio (224) | Whole blood | IV: 90 | Hematology analyzer | X |  |  | 2.4 | Corticosteroids were not used by patients |
| Neutrophil-Lymphocyte -Ratio (159) | Whole blood | III: 13; IV: 151 | Hematology analyzer |  |  | X |  | Corticosteroid were used by some patients |
| Neutrophil-Lymphocyte -Ratio (161) | Whole blood | I-II: 77 ; III-IV:101 | Hematology analyzer |  |  | X |  | Corticosteroids were not used by patients |
| Neutrophil-Lymphocyte -Ratio (183) | Whole blood | IV: 497 | Hematology analyzer |  |  | X |  |  |
| Neutrophil-Lymphocyte -Ratio (190) | Whole blood | IV: 84 | Hematology analyzer |  |  | X |  |  |
| Neutrophil-Lymphocyte -Ratio (211) | Whole blood | IV: 80 | Hematology analyzer |  |  | X |  | Corticosteroid were used by some patients |
| Neutrophil-Lymphocyte -Ratio (212) | Whole blood | IV: 140 | Hematology analyzer |  |  |  |  |  |
| Neutrophil-Lymphocyte -Ratio (225) | Whole blood | IV: 85 | Hematology analyzer |  |  | X |  | Corticosteroids were not used by patients |
| Platelet-Lymphocyte -Ratio (222) | Whole blood | IV: 166 | Hematology analyzer | X |  |  | 2.1 | Corticosteroids were not used by patients |
| Platelet-Lymphocyte -Ratio (162) | Whole blood | I-II: 57; III-IV:162 | Hematology analyzer |  |  | X |  |  |
| Platelet-Lymphocyte -Ratio (165) | Whole blood | I: 23; II: 36; III: 35; IV18 | Hematology analyzer |  |  | X |  | Corticosteroids were not used by patients |
| Platelet-Lymphocyte -Ratio (184) | Whole blood | II: 20; III: 53; IV: 86 | Hematology analyzer |  |  | X |  | Corticosteroids were not used by patients |
| Platelet-Lymphocyte -Ratio (192) | Whole blood | II-III: 404; IV: 188 | Hematology analyzer |  |  | X |  |  |
| Platelet-Lymphocyte -Ratio (180) | Whole blood | IV: 187 | Hematology analyzer |  |  | X |  | Corticosteroids were not used by patients |
| Platelet-Lymphocyte -Ratio (190) | Whole blood | IV: 84 | Hematology analyzer |  |  | X |  |  |
| Platelet-Lymphocyte -Ratio (210) | Whole blood | IV: 152 | Hematology analyzer |  |  | X |  | Corticosteroids were not used by patients |
| Platelet-Lymphocyte -Ratio (211) | Whole blood | IV: 80 | Hematology analyzer |  |  | X |  | Corticosteroid were used by some patients |
| Platelet-Lymphocyte -Ratio (212) | Whole blood | IV 140 | Hematology analyzer |  |  | X |  |  |
| Platelet-Lymphocyte -Ratio (221) | Whole blood | IV: 192 | Hematology analyzer |  |  | X |  |  |
| Platelet-Lymphocyte -Ratio (224) | Whole blood | IV: 90 | Hematology analyzer |  |  | X |  | Corticosteroids were not used by patients |
| Monocyte-Lymphocyte -Ratio (190) | Whole blood | IV: 84 | Hematology analyzer |  |  | X |  |  |
| Monocyte-Lymphocyte -Ratio (221) | Whole blood | IV: 192 | Hematology analyzer |  |  | X |  |  |
| Monocyte-Lymphocyte -Ratio (222) | Whole blood | IV: 166 | Hematology analyzer |  |  | X |  | Corticosteroids were not used by patients |
| Monocyte-Lymphocyte -Ratio (160) | Whole blood | I-II: 59; III-IV: 124 | Hematology analyzer |  |  | X |  |  |
| Monocyte-Lymphocyte -Ratio (192) | Whole blood | II-III: 404; IV: 188 | Hematology analyzer |  |  | X |  |  |
| Systemic immune-inflammation index (221) | Whole blood | IV: 192 | Hematology analyzer | X |  |  | 0.6 |  |
| Systemic immune-inflammation index (166) | Whole blood | III: 14; IV: 102 | Hematology analyzer | X |  |  | 1.6 |  |
| Myeloid derived suppressor cells (177) | Whole blood | II-IV: 40 | Flow cytometry | X |  |  |  |  |
| Glucose (226) | Serum | III: 70; IV: 297 | Not specified | X |  |  |  |  |
| Glucose (167) | Not specified | II: 65; III: 38; IV: 72 | Not specified | X |  |  | 1.2 |  |
| Glucose (227) | Not specified | IV: 106 | Not specified | X |  |  |  |  |
| Glucose (228) | Not specified | IV: 191 | Not specified | X |  |  |  | Corticosteroid were used by some patients |

# Supplemental Table 7: Predictive marker table

| Marker (reference number) | Biosource | Patient population with less malignant type of marker | Patient population with more malignant type of marker | Biomarker detection methodology | Marker significantly increased in glioma or glioblastoma patients with more malignant type of histopathological marker | Marker significantly decreased in glioma or glioblastoma patients with more malignant type of histopathological marker | Marker non-significantly changed in glioma or glioblastoma patients | Marker AUC, accuracy or sensitivity (SE) and specificity (SP) if measured | Medication use before blood sampling in all or some patients if reported |
| --- | --- | --- | --- | --- | --- | --- | --- | --- | --- |
| CRP (184) | Serum | 79  IDH-mutant glioma patients | 80 IDH-WT glioma patients | Not specified | X |  |  |  | Corticosteroids were not used by patients |
| Albumin-globulin-ratio (142) | Serum | 311 IDH-mutant glioma patient | 395 IDH-WT glioma patients | Not specified |  | X |  |  |  |
| Fibrinogen (142) | Serum | 311 IDH-mutant glioma patient | 395 IDH-WT glioma patients | Not specified |  |  | X |  |  |
| Albumin (142) | Serum | 311 IDH-mutant glioma patient | 395 IDH-WT glioma patients | Not specified |  |  | X |  |  |
| Prognostic nutritional index (142) | Serum | 311 IDH-mutant glioma patient | 395 IDH-WT glioma patients | Not specified |  |  | X |  |  |
| GFAP (43) | Serum | 8 IDH-mutant glioma patients | 27 IDH-WT glioma patients | ELISA | X |  |  |  | Corticosteroid were used by some patients |
| GFAP (43) | Serum | 14 glioma patients with high Ki67 proliferation index | 13 glioma patients with high Ki67 proliferation index | ELISA | X |  |  |  |  |
| MGMT methylation in cfDNA (229) | Serum | 17 astrocytic glioma patients without MGMT methylation | 24 astrocytic glioma patients without MGMT methylation | Methylation-specific PCR | X |  |  | SE: 59%  SP: 100% |  |
| MGMT methylation in cfDNA (229) | Serum | 15 oligodendro-glial glioma patients with MGMT methylation | 14 oligodendro-glial glioma patients without MGMT methylation | Methylation-specific PCR |  |  |  |  |  |
| MGMT methylation in cfDNA (230) | Serum | 51 glioma patients with MGMT methylation | 38 glioma patients without MGMT methylation | Methylation-specific PCR | X |  |  | SE: 38%  SP: 100% |  |
| MGMT methylation in cfDNA (231) | Serum | 2 glioma patients with MGMT methylation | 7 glioma patients without MGMT methylation | Methylation-specific polymerase chain reaction | X |  |  | SE: 50%  SP: 100% |  |
| MGMT methylation in cfDNA (232) | Plasma | 2 glioma patients with MGMT methylation | 8 glioma patients without MGMT methylation | Methylation specific polymerase chain reaction | X |  |  | SE: 50%  SP: 100% |  |
| MGMT methylation in cfDNA (233) | Plasma | 35 glioma patients with MGMT methylation | 38 glioma patients without MGMT methylation | Methylation-specific PCR | X |  |  | SE: 31%  SP: 96% |  |
| MGMT methylation in cfDNA (233) | Plasma | 39 glioma patients with MGMT methylation | 35 glioma patients without MGMT methylation | Pyrosequencing | X |  |  | SE: 38%  SP: 76% |  |
| IDH mutation in cfDNA (234) | Plasma | 25 glioma patients with IDH-mutant type glioma | 14 glioma patients with IDH-wild type glioma | Digital PCR | X |  |  |  |  |
| Lymphocytes (155) | Whole blood | 144 glioma patients with IDH-mutant type glioma | 139 glioma patients with IDH-wild type glioma | Hematology analyzer |  |  | X |  |  |
| Neutrophils (155) | Whole blood | 144 glioma patients with IDH-mutant type glioma | 139 glioma patients with IDH-wild type glioma | Hematology analyzer | X |  |  |  |  |
| Neutrophil-Lymphocyte-Ratio (142) | Whole blood | 311 IDH-mutant glioma patient | 395 IDH-WT glioma patients | Hematology analyzer | X |  |  | X |  |
| Neutrophil-Lymphocyte-Ratio (155) | Whole blood | 144 glioma patients with IDH-mutant type glioma | 139 glioma patients with IDH-wild type glioma | Hematology analyzer | X |  |  | X |  |
| Neutrophil-Lymphocyte-Ratio (156) | Whole blood | 167 glioma patients with IDH-mutant type glioma | 158 glioma patients with IDH-wild type glioma | Hematology analyzer | X |  |  |  |  |
| Neutrophil-Lymphocyte-Ratio (184) | Whole blood | 79  IDH-mutant glioma patients | 80 IDH-WT glioma patients | Hematology analyzer | X |  |  |  | Corticosteroids were not used by patients |
| Neutrophil-Lymphocyte-Ratio (157) | Whole blood | 24  IDH-mutant glioma patients | 81 IDH-WT glioma patients | Hematology analyzer |  |  | X |  |  |
| Neutrophil-Lymphocyte-Ratio (160) | Whole blood | 56 patients with low ki-67 proliferation index | 127 patients with high ki-67 proliferation index | Hematology analyzer | X |  |  |  |  |
| Neutrophil-Lymphocyte-Ratio (221) | Whole blood | 47 patients with low ki-67 proliferation index | 64 patients with high ki-67 proliferation index | Hematology analyzer | X |  |  |  |  |
| Neutrophil-Lymphocyte-Ratio (210) | Whole blood | 53 glioma patients with MGMT methylation | 99 glioma patients with MGMT methylation | Hematology analyzer |  |  | X |  | Corticosteroids were not used by patients |
| Neutrophil-Lymphocyte-Ratio (221) | Whole blood | 37 glioma patients with MGMT methylation | 28 glioma patients with MGMT methylation | Hematology analyzer |  |  | X |  |  |
| Neutrophil-Lymphocyte-Ratio (221) | Whole blood | 38 glioma patients with IDH mutation | 30 glioma patients with IDH wild type | Hematology analyzer |  |  | X |  |  |
| Platelet-Lymphocyte-Ratio (142) | Whole blood | 311 glioma patients with IDH mutation | 395 glioma patients with IDH wild type | Hematology analyzer | X |  |  |  |  |
| Platelet-Lymphocyte-Ratio (155) | Whole blood | 144 glioma patients with IDH-mutant type glioma | 139 glioma patients with IDH-wild type glioma | Hematology analyzer |  |  | X |  |  |
| Platelet-Lymphocyte-Ratio (184) | Whole blood | 79  IDH-mutant glioma patients | 80 IDH-WT glioma patients | Hematology analyzer |  |  | X |  | Corticosteroids were not used by patients |
| Platelet-Lymphocyte-Ratio (221) | Whole blood | 38 glioma patients with IDH mutation | 30 glioma patients with IDH wild type | Hematology analyzer |  |  | X |  |  |
| Platelet-Lymphocyte-Ratio (222) | Whole blood | 31 glioma patients with IDH mutation | 135 glioma patients with IDH wild type | Hematology analyzer |  |  | X |  | Corticosteroids were not used by patients |
| Platelet-Lymphocyte-Ratio (221) | Whole blood | 37 glioma patients with MGMT methylation | 28 glioma patients with MGMT methylation | Hematology analyzer |  |  | X |  |  |
| Platelet-Lymphocyte-Ratio (221) | Whole blood | 47 patients with low ki-67 proliferation index | 64 patients with high ki-67 proliferation index | Hematology analyzer |  |  | X |  |  |
| Monocyte-lymphocyt e-Ratio (222) | Whole blood | 31 glioma patients with IDH mutation | 135 glioma patients with IDH wild type | Hematology analyzer |  |  | X |  | Corticosteroids were not used by patients |
| Monocyte-lymphocyte-Ratio (160) | Whole blood | 56 patients with low ki-67 proliferation index | 127 patients with high ki-67 proliferation index | Hematology analyzer |  |  | X |  |  |
| Systemic immune-inflammation index (153) | Whole blood | 6 patients with low ki-67 proliferation index | 94 patients with high ki-67 proliferation index | Hematology analyzer | X |  |  |  | Corticosteroids were not used by patients |
| Systemic immune-inflammation index (221) | Whole blood | 37 glioma patients with MGMT methylation | 28 glioma patients with MGMT methylation | Hematology analyzer |  |  | x |  |  |
| Systemic immune-inflammation index (221) | Whole blood | 47 patients with low ki-67 proliferation index | 64 patients with high ki-67 proliferation index | Hematology analyzer |  |  | X |  |  |
| Systemic immune-inflammation index (221) | Whole blood | 38 glioma patients with IDH mutation | 30 glioma patients with IDH wild type | Hematology analyzer |  |  | X |  |  |
| EGFR+ EVs (140) | Serum | Not specified | Not specified | Flow cytometry | X |  |  |  |  |

# Supplemental Table 8: Therapy monitoring marker table for tumor volume

| Marker (reference number) | Biosource | Glial tumor grades of glioma patients in study population if specified | Biomarker detection methodology | Marker increased in glioma or glioblastoma patients with higher tumor volume | Marker decreased in glioma or glioblastoma patients with lower tumor volume | Marker non-significantly changed in glioma or glioblastoma patients with high compared to low tumor volumes | Medication use before blood sampling in all or some patients if reported |
| --- | --- | --- | --- | --- | --- | --- | --- |
| S100b (27) | Serum | III-IV: 14 | ELISA |  |  | X |  |
| S100b (28) | Serum | III: 4; IV: 27 | Turbidimetric method |  |  | X | Corticosteroid were used by some patients |
| CRP (38) | Plasma | IV: 14 | Quantitative targeted absolute proteomics | X |  |  |  |
| Complement C9 (38) | Plasma | IV: 14 | Quantitative targeted absolute proteomics | X |  |  |  |
| GFAP (28) | Serum | III: 4; IV: 27 | Turbidimetric method | X |  |  | Corticosteroid were used by some patients |
| GFAP (43) | Serum | III: 13; IV: 14 | ELISA | X | IV: 14 III: 13 | ELISA | Corticosteroid were used by some patients |
| GFAP (44) | Serum | IV: 50 | ELISA | X |  |  | Corticosteroid were used by some patients |
| GFAP (236) | Serum | IV: 33 | ELISA | X |  |  |  |
| GFAP (47) | Plasma | IV: 111 | ELISA | X |  |  |  |
| GFAP (45) | Serum | IV: 33 | Immuno-fluorescence assay |  |  | X |  |
| YKL-40 (50) | Serum | I-II: 41; III-IV: 197 | ELISA |  |  | X |  |
| YKL-40 (149) | Serum | II-III: 20; IV: 45 | ELISA |  |  | X |  |
| YKL-40 (196) | Serum | III: 66 ; IV 77 | ELISA |  |  | X |  |
| YKL-40 (47) | Plasma | IV: 111 | ELISA |  |  | X |  |
| VEGF (2) | Serum | 32 glioma patients | ELISA |  |  | X |  |
| MicroRNA-21 (74) | Serum | IV: 20 | qRT-PCR | X |  |  |  |
| Total cfdna (87) | Plasma | IV: 42 | qRT-PCR | X |  |  |  |
| Total cfdna (237) | Plasma | IV: 8 | Agilent 4200 TapeStation System |  |  | X |  |
| Total cfdna (238) | Plasma | IV: 42 | Guardant Health (Guardant360) |  |  | X |  |
| Alu methylation (89) | Serum | I-II: 38; III-IV: 71 | DNA sequencing |  |  | X |  |
| Alu methylation (90) | Serum | I-II: 32; III-IV: 33 | DNA sequencing |  |  | X |  |
| Circulating glial tumor cells (101) | Whole blood | IV: 13 | Immunostaining |  |  | X |  |
| Neutrophil-Lymphocyte-Ratio (180) | Whole blood | IV: 187 | Hematology analyzer |  |  | X | Corticosteroids were not used by patients |
| Monocyte-Lymphocyte-Ratio (162) | Whole blood | I-II: 57; III-IV:162 | Hematology analyzer |  |  | X |  |
| Systemic immune-inflammation index (166) | Whole blood | III: 14; IV: 102 | Hematology analyzer |  |  | X |  |
| Extracellular vesicles (134) | Plasma | IV: 43 | Transmission electron microscopy |  |  | X |  |

# Supplemental Table 9: Therapy monitoring marker table for tumor progression

| Marker (reference number) | Biosource | Glial tumor grades of glioma patients in study population if specified | Biomarker detection methodology | Marker increased in glioma or glioblastoma patients with tumor progression as opposed to stable disease or pseudoprogression | Marker decreased in glioma or glioblastoma patients with tumor progression as opposed to stable disease or pseudoprogression | Marker non-significantly changed in glioma or glioblastoma patients with tumor progression as opposed to stable disease or pseudoprogression |
| --- | --- | --- | --- | --- | --- | --- |
| Circulating glial tumor cell (95) | Whole blood | II: 11; III: 9; IV: 12 | Immunofluorescence | X |  |  |
| Circulating glial tumor cell (96) | Whole blood | II-IV: 11 | Telomerase promoter assay | X |  |  |
| Circulating glial tumor cell (99) | Whole blood | IV: 33 | STEAM immunofluorescence | X |  |  |
| Neutrophil-lymphocyte-ratio (220) | Whole blood | III-IV: 135 | Hematology analyzer | X |  |  |
| Microvesicle count (239) | Plasma | IV: 11 | Flow cytometry | X |  |  |
| CfDNA amount (237) | Plasma | IV: 8 | Agilent 4200 TapeStation System | X |  |  |
| Extracellular vesicle amount (134) | Plasma | IV: 43 | Transmission electron microscopy | X |  |  |

# Supplemental Table 10: Panels of Biomarkers

| Markers within panel (reference number) | Biomarker type | Biosource | Glial tumor grades of glioma patients in study population if specified | Control population | Biomarker detection methodology | Biomarker function | Marker AUC, accuracy or sensitivity (SE) and specificity (SP) if measured | Hazard ratio | Medication use before blood sampling in all or some patients if reported |
| --- | --- | --- | --- | --- | --- | --- | --- | --- | --- |
| miR-182-5p, miR-328-3p, miR-339-5p, miR-340-5p, miR-485-3p, miR-486-5p, and miR-543 (240) | miRNA | Serum | IV: 12 | 9 healthy individuals | RNA sequencing | Diagnostic | Acc.: 92% |  |  |
| RNU6-1, miR-320 and miR-574-3p (241) | RNA and miRNA | Serum | IV: 50 | 30 healthy individuals | qRT-PCR | Diagnostic | AUC: 0.8 |  |  |
| Panel of 180 miRNAs (242) | miRNA | Peripheral blood | IV: 20 | 20 healthy individuals | RT-PCR | Diagnostic | Acc.: 81%  SE: 83%  SP: 79% |  |  |
| miR-320e, miR-223, miR-16-5p, miR-484, miR-520a, miR-532, miR-630, miR651, miR-761 (70) | miRNA | Serum | I-II: 47; IV: 44 | 17 healthy individuals | Droplet digital PCR | Diagnostic | Acc.: 99.8% |  |  |
| Mir-15b, mir-21 (71) | miRNA | Serum | II: 8; III: 6; IV: 16 | 30 patients with miscellaneous neurologic disorders | qRT-PCR | Diagnostic | SE: 90%  SP: 100% |  |  |
| miR-21, 222 and mir-124-3 (72) | miRNA | Serum | IV: 44 | 30 healthy individuals | qRT-PCR | Diagnostic | AUC: 0.9 |  |  |
| miR-21, 222 and mir-124-3 (72) | miRNA | Serum | III-IV: 60 | I-II: 15 | qRT-PCR | Tumor grade differentiation | AUC: 0.8 |  |  |
| miR-15b-5p, miR-16-5p, miR-19a-3p, miR-19b-3p, miR-20a-5p, miR-106a-5p, miR-130a-3p, miR-181b-5p, and miR-208a-3p (77) | miRNA | Serum | 50 astrocytoma patients | 50 healthy individuals | qRT-PCR |  | AUC: 1.0 |  |  |
| miR-19a-3p, miR-106a-5p, miR-181b-5p (77) | miRNA | Serum | 90 astrocytoma patients of which some with short survival | 90 astrocytoma patients of which some with long survival | qRT-PCR | Prognostic |  | HR: 3.1 |  |
| miR-4763-3p, miR-1915-3p, and miR-3679-5p (243) | miRNA | Serum | 57 diffuse glioma patients in validation set | 114 healthy individuals in validation set | Microarray | Diagnostic | AUC: 1.0 |  |  |
| Panel of 48 miRNAs (243) | miRNA | Serum | 17 Glioblastoma patients in validation set | 8 PCNSL patients, 5 cerebral metastasis patients in validation set | Microarray | Diagnostic | SE: 94% |  |  |
| miR-497 and miR-125b (244) | miRNA | Serum | IV: 10 | II-III: 12 | qRT-PCR | Tumor grade differentiation | AUC: 0.9 |  |  |
| miR-363-3p, miR-93-3p, miR-22-5p, miR-451a, miR-222-3p and miR-140-3p  (173) | miRNA | Plasma | 17 glioma patients with postsurgical pulmonary embolisms | 33 glioma patients without postsurgical pulmonary embolisms | qRT-PCR | Risk prediction for postsurgical pulmonary embolisms | AUC: 0.8 |  |  |
| IL10, IL17, IL15, MIP1α, IL2, LIF, TNFα, IL6, FGFbasic, IL4, GM-CSF, IFNγ, IL7, IL1Rα, SCGFβ, IL12/P40, βNGF, IL3 (8) | Proteins | Serum | IV: 74 glioma patients in validation set | 13 healthy individuals in validation set | Human cytokine kits | Diagnostic | ACC.: 97% |  |  |
| IL10, IL17, IL15, MIP1α, IL2, LIF, TNFα, IL6, FGFbasic, IL4, GM-CSF, IFNγ, IL7, IL1Rα, SCGFβ, IL12/P40, βNGF, IL3 (8) | Proteins | Serum | II: 24 | 26 healthy individuals | Human cytokine kits | Diagnostic | ACC.: 96% |  |  |
| IL10, IL17, IL15, MIP1α, IL2, LIF, TNFα, IL6, FGFbasic, IL4, GM-CSF, IFNγ, IL7, IL1Rα, SCGFβ, IL12/P40, βNGF, IL3 (8) | Proteins | Serum | III: 22 | 26 healthy individuals | Human cytokine kits | Diagnostic | ACC.: 96% |  |  |
| Panel of markers with mass spectrometry peaks at: m/z values 1296.77, 2105.86, 2769.20,  2932.82,  4210.20,  4266.27, 4964.31, 5634.09, 6379.58,  6530.04, 8142.23  (245) | Peptides | Serum | I-II: 24 ; III: 15 ; IV: 14 | 69 healthy individuals | MALDI-TOF mass spectrometry | Diagnostic | AUC: 1.0 |  |  |
| Panel of markers with 47 mass spectrometry peaks (246) | Proteins | Serum | 26 astrocytoma patients in validation set | 22 healthy individuals in validation set | SELDI-TOF mass spectrometry | Diagnostic | SE: 85%  SP: 86% |  |  |
| Panel of markers with mass spectrometry peaks at: 8214.77 m/z, 8926.76 m/z, 4815.11 m/z, 8612.23 m/z, 2082.19 m/z, 4299.87 m/z, 2103.55 m/z, 7764.82 m/z, 2368.19 m/z, 3226.97 m/z, 2389.55 m/z, 2021.78 m/z, 4469.09 m/z, 6457.054 m/z, 8702.416 m/z (247) | Proteins | Serum | II-III: 9 astrocytoma patients | 13 patients with benign brain tumors | SELDI-TOF-MS | Tumor type differentiation | ACC.: 96% |  |  |
| Panel of markers with mass spectrometry peaks at: 2256.76 m/z, 23481.05 m/z, 9198.31 m/z, 22513.91 m/z, 22888.73 m/z, 23087.61 m/z, 4155.28 m/z, 2489.11 m/z, 2246.47 m/z, 2617.14 m/z, 15099.38 m/z, 22666.41 m/z, 29047.13 m/z, 14378.33 m/z, 24002.85 m/z, 2891.43 m/z, 14047.77 m/z, 2006.00 m/z, 14951.04 m/z, 2267.22 m/z, 23672.58 m/z, 22331.29 m/z (247) | Proteins | Serum | III-IV: 13 | I-II: 15 | SELDI-TOF-MS | Tumor grade differentiation | Acc.: 86% |  |  |
| NGPT1, TIMP1, IP10, and TGFβ1 (248) | Proteins | Serum | 59 astrocytoma patients | 43 healthy individuals | Human Protein Antibody Array | Diagnostic | Acc.: 74% |  |  |
| OPN and IP10 (248) | Proteins |  | 13 GBM patients who survived less than one year | 26 patients who survived more than one year | Human Protein Antibody Array | Prognostic |  |  |  |
| Panel of 274 peptides (249) | Peptides | Serum | 34 glioma patients | 22 healthy controls | MALDI-TOF Mass Spectrometry | Diagnostic | Acc.: 96% |  |  |
| IFN-γ/TNF-α/IL-17a (250) | Protein | Serum | 145 glioblastoma patients of which some with short survival | 145 glioblastoma patients of which some with long survival | ELISA | Prognostic |  | HR: 2.3 |  |
| Panel of 11 protein (12) | Protein | Plasma | IV: 18 | 17 healthy controls | Antibody microarray | Diagnostic | AUC: 0.6 |  |  |
| IL12A, LYAM1, BHE40, CRP, SSR4 (37) | Protein | Serum | IV: 28 | 27 healthy controls | Antibody microarray | Diagnostic | Acc.: 90% |  |  |
| GFAP and YKL-40 (47) | Protein | Plasma | IV: 111 | 99 healthy controls | ELISA | Diagnostic | AUC: 0.8 |  |  |
| Panel of 6 metabolites (170) | Metabolites | Plasma | IDH-WT glioma patients | IDH-mutant glioma patients | Liquid chromatography triple quadrupole mass spectrometry | Prediction of mutation of IDH-gene in tumor | ACC.: 94% |  | Medication use: corticosteroids, antiglycemic medication, seizure medication |
| Panel of 18 metabolites (170) | Metabolites | Plasma | III-IV: 28 | I-II: 17 | Liquid chromatography triple quadrupole mass spectrometry | Tumor grade differentiation | ACC.: 91% |  | Medication use: corticosteroids, antiglycemic medication, seizure medication |
| Creatine, Citrate, Glucose, Pyruvate, Glutamine (251) | Metabolites | Plasma | IV: 28 | 28 healthy volunteers | NMR spectroscopy | Diagnostic | AUC: 1.0 |  |  |
| Citrate, Creatinine, Formate, Creatine, Glucose (251) | Metabolites | Plasma | II-III: 10 oligodendroglioma patients | 28 healthy volunteers | NMR spectroscopy | Diagnostic | AUC: 0.9 |  |  |
| Creatine, Citrate, Formate, Creatinine, Glucose (251) | Metabolites | Plasma | II-III: 5 astrocytoma patients | 28 healthy volunteers | NMR spectroscopy | Diagnostic | AUC: 0.9 |  |  |
| Creatine, Citrate, Formate, Glucose, Pyruvate (251) | Metabolites | Plasma | II-IV: 49 glioma patients | 28 healthy volunteers | NMR spectroscopy | Diagnostic | AUC: 0.9 |  |  |
| Panel of 3 metabolites (251) | Metabolites | Plasma | IV: 28 | 11 meningioma patients | NMR spectroscopy | Tumor type differentiation | AUC: 0.7 |  |  |
| Panel of 3 metabolites (251) | Metabolites | Plasma | IV: 28 | I-II: 12 | NMR spectroscopy | Tumor grade differentiation | AUC: 0.7 |  |  |
| Panel of 5 metabolites (252) | Metabolites | Serum | 57 glioblastoma of which some patients with short survival | 57 glioblastoma of which some patients with short survival | GC-TOF-MS | Prognostic | AUC: 1.0 |  |  |
| Panel of 13 metabolites (252) | Metabolites | Serum | 20 glioblastoma of which some patients with short survival | 20 glioblastoma of which some patients with short survival | GC-TOF-MS | Prognostic | AUC: 1.0 |  |  |
| Panel of 13 metabolites (252) | Metabolites | Serum | IV: 57 | 20  Oligodendroglioma | GC-TOF-MS | Tumor type differentiation | AUC: 0.8 |  |  |
| Panel of 12 metabolites (252) | Metabolites | Serum | 20  oligodendroglioma of which some with grade III tumors | 20  oligodendroglioma of which some with grade II tumors | GC-TOF-MS | Tumor grade differentiation | AUC: 0.9 |  |  |
| Arginine, methionine, and kynurenate (254) | Metabolites | Plasma | IV: 33 with short survival | IV: 46 with short survival | Liquid chromatography triple quadrupole mass spectrometry | Prognostic |  | HR: 2.4 | Corticosteroid were used by some patients, anti-epileptic and anti-glycemic drug use |

# Supplementary table 11: Glioma biomarkers involved in hallmarks or enabling characteristics of cancer

| Current hallmark, emerging hallmark or enabling characteristic | Biomarker as mediating or expressing factor in a hallmark or enabling characteristic |
| --- | --- |
| Sustaining proliferative signalling | miR-182^256^; miR-21^257^ |
| Evading growth suppressors | - |
| Resisting cell death | IL-6^258^; YKL-40^259^; miR-21^260^; IL-1b^261^ |
| Enabling replicative immortality | - |
| Inducing angiogenesis | IL-6^262^; IL1b^263^; YKL-40^259^; VEGF^264^; S100^265^; TNF^266^ |
| Activating invasion and metastasis | IL-1b^261^; circulating glial tumor cell count; miR-182^267^; IL-6^262^; S100^265^; TNF^266^ |
| Deregulated cellular energetics | Metabolites implicated in amino acid metabolism^170,252,255,268^; lipid metabolism^252,255,268^; nucleic acid metabolism^170,255^ and carbohydrate metabolism^170,252,255,268^ |
| Avoiding immune destruction | IL-10^269,270^; MDSC count; regulatory T-cell count; immature dendritic cell count; dendritic, CD4+- and CD8+-cell counts; M2-macrophage cell count |
| Genome instability and mutation | Alu methylation in cfDNA^89,90^; cfDNA methylome^88^; MGMT methylation in cfDNA^229–233^; DNA mutations in circulating glial tumor cells^101^; IDH1 mutation in cfDNA^234^; ctDNA^87,92,152,238,271,272^ |
| Tumor promoting inflammation | Various APRPs and inflammatory cell populations; TNF^273^; S100^274^ |

**References**

1. Nijaguna MB, Patil V, Urbach S, et al. Glioblastoma-derived macrophage colony-stimulating factor (MCSF) induces microglial release of insulin-like growth factor-binding protein 1 (IGFBP1) to promote angiogenesis. *J. Biol. Chem.* 2015;290(38):23401–23415.

2. Shamsdin SA, Mehrafshan A, Rakei SM, Mehrabanilare D. Evaluation of VEGF, FGF and PDGF and Serum levels of inflammatory cytokines in patients with Glioma and Meningioma in Southern Iran. *Asian Pacific J. Cancer Prev.* 2019;20(10):2883–2890.

3. Ren J, Jia P, Feng H, et al. Involvement of poly(Adp-ribose) polymerase-1 in chinese patients with gliomaa potential target for effective patient care. *Int. J. Biol. Markers*. 2018;33(1):68–72.

4. Gao Y, Zhang E, Liu B, et al. Integrated analysis identified core signal pathways and hypoxic characteristics of human glioblastoma. *J. Cell. Mol. Med.* 2019;23(9):6228–6237.

5. Albulescu R, Codrici E, Popescu ID, et al. Cytokine patterns in brain tumour progression. *Mediators Inflamm.* 2013;2013:979748.

6. Löhr M, Freitag B, Technau A, et al. High-grade glioma associated immunosuppression does not prevent immune responses induced by therapeutic vaccines in combination with Treg depletion. *Cancer Immunol. Immunother.* 2018;67(10):1545–1558.

7. Bryant NL, Suarez-Cuervo C, Gillespie GY, et al. Characterization and immunotherapeutic potential of γδ T-cells in patients with glioblastoma. *Neuro. Oncol.* 2009;11(4):357–367.

8. Nijaguna MB, Patil V, Hegde AS, et al. An eighteen serum cytokine signature for discriminating Glioma from normal healthy individuals. *PLoS One*. 2015;10(9):e0137524.

9. Shan Y, He X, Song W, et al. Role of IL-6 in the invasiveness and prognosis of glioma. *Int. J. Clin. Exp. Med.* 2015;8(6):9114–20.

10. Reynés G, Vila V, Martín M, et al. Circulating markers of angiogenesis, inflammation, and coagulation in patients with glioblastoma. *J. Neurooncol.* 2011;102(1):35–41.

11. Deniz ÇD, Gürbilek M, Koç M. Prognostic value of interferon-gamma, interleukin-6, and tumor necrosis factor-alpha in the radiation response of patients diagnosed with locally advanced non-small-cell lung cancer and glioblastoma multiforme. *Turkish J. Med. Sci.* 2018;48(1):117–123.

12. Carlsson A, Persson O, Ingvarsson J, et al. Plasma proteome profiling reveals biomarker patterns associated with prognosis and therapy selection in glioblastoma multiforme patients. *Proteomics - Clin. Appl.* 2010;4(6–7):591–602.

13. Doroudchi M, Pishe ZG, Malekzadeh M, et al. Elevated serum IL-17A but not IL-6 in glioma versus meningioma and schwannoma. *Asian Pacific J. Cancer Prev.* 2013;14(9):5225–5230.

14. Kmiecik J, Poli A, Brons NHC, et al. Elevated CD3+ and CD8+ tumor-infiltrating immune cells correlate with prolonged survival in glioblastoma patients despite integrated immunosuppressive mechanisms in the tumor microenvironment and at the systemic level. *J. Neuroimmunol.* 2013;264(1–2):71–83.

15. Hu J, Mao Y, Li M, Lu Y. The profile of Th17 subset in glioma. *Int. Immunopharmacol.* 2011;11(9):1173–1179.

16. Demirci U, Yaman M, Buyukberber S, et al. Prognostic importance of markers for inflammation, angiogenesis and apoptosis in high grade glial tumors during temozolomide and radiotherapy. *Int. Immunopharmacol.* 2012;14(4):546–549.

17. Hands JR, Abel P, Ashton K, et al. Investigating the rapid diagnosis of gliomas from serum samples using infrared spectroscopy and cytokine and angiogenesis factors. *Anal. Bioanal. Chem.* 2013;405(23):7347–7355.

18. Holst CB, Christensen IJ, Skjøth-Rasmussen J, et al. Systemic Immune Modulation in Gliomas: Prognostic Value of Plasma IL-6, YKL-40, and Genetic Variation in YKL-40. *Front. Oncol.* 2020;10:478.

19. Xu BJ, An QA, Gowda SS, et al. Identification of blood protein biomarkers that aid in the clinical assessment of patients with malignant glioma. *Int. J. Oncol.* 2012;40(6):1995–2003.

20. Gousias K, Markou M, Arzoglou V, et al. Frequent abnormalities of the immune system in gliomas and correlation with the WHO grading system of malignancy. *J. Neuroimmunol.* 2010;226(1–2):136–142.

21. Mostafa H, Pala A, Högel J, et al. Immune phenotypes predict survival in patients with glioblastoma multiforme. *J. Hematol. Oncol.* 2016;9(1):77.

22. Arora A, Patil V, Kundu P, et al. Serum biomarkers identification by iTRAQ and verification by MRM: S100A8/S100A9 levels predict tumor-stroma involvement and prognosis in Glioblastoma. *Sci. Rep.* 2019;9(1):1–12.

23. Popescu ID, Codrici E, Albulescu L, et al. Potential serum biomarkers for glioblastoma diagnostic assessed by proteomic approaches. *Proteome Sci.* 2014;12(1):47.

24. Gautam P, Nair SC, Gupta MK, et al. Proteins with Altered Levels in Plasma from Glioblastoma Patients as Revealed by iTRAQ-Based Quantitative Proteomic Analysis. *PLoS One*. 2012;7(9):e46153.

25. Zupancic K, Blejec A, Herman A, et al. Identification of plasma biomarker candidates in glioblastoma using an antibody-array-based proteomic approach. *Radiol. Oncol.* 2014;48(3):257–266.

26. Gielen PR, Schulte BM, Kers-Rebel ED, et al. Elevated levels of polymorphonuclear myeloid-derived suppressor cells in patients with glioblastoma highly express S100A8/9 and arginase and suppress T cell function. *Neuro. Oncol.* 2016;18(9):1253–1264.

27. Oktay K, Olguner SK, Sarac ME, et al. Evaluation of Serum S100B Values in High Grade Glioma Patients. *J. Neurol. Sci.* 2015;32(4):738–746.

28. Brommeland T, Rosengren L, Fridlund S, Hennig R, Isaksen V. Serum levels of glial fibrillary acidic protein correlate to tumour volume of high-grade gliomas. *Acta Neurol. Scand.* 2007;116(6):380–384.

29. Lyubimova N V, Toms MG, Popova EE, et al. Neurospecific Proteins in the Serum of Patients with Brain Tumors. *Eksp. Biol. i Meditsiny*. 2010;150(12):678–681.

30. Rahbar A, Cederarv M, Wolmer-Solberg N, et al. Enhanced neutrophil activity is associated with shorter time to tumor progression in glioblastoma patients. *Oncoimmunology*. 2016;5(2):e1075693.

31. Chiorean R, Berindan-Neagoe I, Braicu C, et al. Quantitative expression of serum biomarkers involved in angiogenesis and inflammation, in patients with glioblastoma multiforme: Correlations with clinical data. *Cancer Biomarkers*. 2014;14(2–3):185–194.

32. Ahluwalia MS, Bou-Anak S, Burgett ME, et al. Correlation of higher levels of soluble TNF-R1 with a shorter survival, independent of age, in recurrent glioblastoma. *J. Neurooncol.* 2017;131(3):449–458.

33. Joseph F. Weiss, Robert A. Morantz WPB and PBC. Serum Acute-Phase Proteins and lmmunoglobulins in Patients with Gliomas. *Cancer Res.* 1979;39:542–544.

34. Matsuura H, Nakazawa S. Prognostic significance of serum alpha 1-acid glycoprotein in patients with glioblastoma multiforme: a preliminary communication. *Neurosurgery, and Psychiatry*. 1985;48(8):835–837.

35. Mohan Kumar D, Thota B, Vijay Shinde S, et al. Proteomic Identification of Haptoglobin alpha2 as a Glioblastoma Serum Biomarker: Implications in Cancer Cell Migration and Tumor Growth. *J Proteome Res .* 2010;9(11):5557–5567.

36. Gollapalli K, Ray S, Srivastava R, et al. Investigation of serum proteome alterations in human glioblastoma multiforme. *Proteomics*. 2012;12(14):2378–2390.

37. Nijaguna MB, Schröder C, Patil V, et al. Definition of a serum marker panel for glioblastoma discrimination and identification of Interleukin 1β in the microglial secretome as a novel mediator of endothelial cell survival induced by C-reactive protein. *J. Proteomics*. 2015;128:251–261.

38. Miyauchi E, Furuta T, Ohtsuki S, et al. Identification of blood biomarkers in glioblastoma by SWATH mass spectrometry and quantitative targeted absolute proteomics. *PLoS One*. 2018;13(3):e0193799.

39. Petrik V, Saadoun S, Loosemore A, et al. Serum α2-HS glycoprotein predicts survival in patients with glioblastoma. *Clin. Chem.* 2008;54(4):713–722.

40. Jaksch-Bogensperger H, Spiegl-Kreinecker S, Arosio P, et al. Ferritin in glioblastoma. *Br. J. Cancer*. 2020;122(10):1441–1444.

41. Zheng SH, Huang JL, Chen M, et al. Diagnostic value of preoperative inflammatory markers in patients with glioma: A multicenter cohort study. *J. Neurosurg.* 2018;129(3):583–592.

42. Liu S, Zhu Y, Zhang C, et al. The Clinical Significance of Soluble Programmed Cell Death-Ligand 1 (sPD-L1) in Patients With Gliomas. *Front. Oncol.* 2020;10:9.

43. Kiviniemi A, Gardberg M, Frantzén J, et al. Serum levels of GFAP and EGFR in primary and recurrent high-grade gliomas: correlation to tumor volume, molecular markers, and progression-free survival. *J. Neurooncol.* 2015;124(2):237–245.

44. C. S. Jung, C. Foerch, A. Schänzer, A. Heck, K. H. Plate, V. Seifert, H. Steinmetz, A. Raabe MS. Serum GFAP is a diagnostic marker for glioblastoma multiforme | Brain | Oxford Academic. *Brain*. 2007;3336–3341.

45. Vietheer JM, Rieger J, Wagner M, et al. Serum concentrations of glial fibrillary acidic protein (GFAP) do not indicate tumor recurrence in patients with glioblastoma. *J. Neurooncol.* 2017;135(1):193–199.

46. Lyubimova N V., Timofeev YS, Mitrofanov AA, et al. Glial Fibrillary Acidic Protein in the Diagnosis and Prognosis of Malignant Glial Tumors. *Bull. Exp. Biol. Med.* 2020;168(4):503–506.

47. Gállego Pérez-Larraya J, Paris S, Idbaih A, et al. Diagnostic and prognostic value of preoperative combined GFAP, IGFBP-2, and YKL-40 plasma levels in patients with glioblastoma. *Cancer*. 2014;120(24):3972–3980.

48. Ilhan-Mutlu A, Wagner L, Widhalm G, et al. Exploratory investigation of eight circulating plasma markers in brain tumor patients. *Neurosurg. Rev.* 2013;36(1):45–56.

49. M H Kazakova, D N Staneva, I G Koev, D G Staikov, N Mateva, P T Timonov, G A Miloshev VSS. Protein and mRNA levels of YKL-40 in high-grade glioma. *Folia Biol*. 2014;60(6):261–270.

50. Iwamoto FM, Hottinger AF, Karimi S, et al. Serum YKL-40 is a marker of prognosis and disease status in high-grade gliomas. *Neuro. Oncol.* 2011;13(11):1244–1251.

51. Gandhi P, Khare R, VasudevGulwani H, Kaur S. Circulatory YKL-40 & NLR: Underestimated prognostic indicators in diffuse glioma. *Int. J. Mol. Cell. Med.* 2018;7(2):111–118.

52. Yang J, Zhao Z, Zhong X. Correlation analysis of the clinicopathological features of glioma and expression of p53 and VEGF. *Int J Clin Exp Med*. 2017;10(2):3606–3611.

53. Nowacka A, Smuczyński W, Rość D, Woźniak—Dąbrowska K, Śniegocki M. Serum VEGF-A concentrations in patients with central nervous system (CNS) tumors. *PLoS One*. 2018;13(3):e0192395.

54. Corsini E, Ciusani E, Gaviani P, et al. Decrease in circulating endothelial progenitor cells in treated glioma patients. *J. Neurooncol.* 2012;108(1):123–129.

55. Salmaggi A, Eoli M, Frigerio S, et al. Intracavitary VEGF, bFGF, IL-8, IL-12 levels in primary and recurrent malignant glioma. *J. Neurooncol.* 2003;62(3):297–303.

56. Rafat N, Beck GC, Schulte J, Tuettenbeg J, Vajkoczy P. Circulating endothelial progenitor cells in malignant gliomas. Clinical article. *J. Neurosurg.* 2010;112(1):43–49.

57. Reynés G, Martínez-Sales V, Vila V, et al. Phase II trial of irinotecan and metronomic temozolomide in patients with recurrent glioblastoma. *Anticancer. Drugs*. 2016;27(2):133–137.

58. Rodrigues JC, Gonzalez GC, Zhang L, et al. Normal human monocytes exposed to glioma cells acquire myeloid-derived suppressor cell-like properties. *Neuro. Oncol.* 2010;12(4):351–365.

59. Labussière M, Cheneau C, Prahst C, et al. Angiopoietin-2 May Be Involved in the Resistance to Bevacizumab in Recurrent Glioblastoma. *Cancer Invest.* 2016;34(1):39–44.

60. Ribom D, Larsson A, Pietras K, Smits A. Growth factor analysis of low-grade glioma CSF: PDGF and VEGF are not detectable. *Neurol. Sci.* 2003;24(2):70–73.

61. Takano S, Yoshii Y, Kondo S, et al. Concentration of Vascular Endothelial Growth Factor in the Serum and Tumor Tissue of Brain Tumor Patients. *Cancer Res.* 1996;56(9):2185–2190.

62. Stockhammer G, Obwegeser A, Kostron H, et al. Vascular endothelial growth factor (VEGF) is elevated in brain tumor cysts and correlates with tumor progression. *Acta Neuropathol.* 2000;100(1):101–105.

63. Crocker M, Ashley S, Giddings I, et al. Serum angiogenic profile of patients with glioblastoma identifies distinct tumor subtypes and shows that TIMP-1 is a prognostic factor. *Neuro. Oncol.* 2011;13(1):99–108.

64. Rość D, Grabarczyk E, Bierwagen M, et al. A preliminary estimation of tissue factor pathway inhibitor (TFPI) and protein C in patients with intracranial tumors. *Adv. Clin. Exp. Med.* 2017;26(8):1219–1224.

65. Marx S, Splittstöhser M, Kinnen F, et al. Platelet activation parameters and platelet-leucocyte-conjugate formation in glioblastoma multiforme patients. *Oncotarget*. 2018;9(40):25860–25876.

66. Yasuo Iwadate, Masayo Hayama, Akihiko Adachi, Tomoo Matsutani, Yuichiro Nagai, Takaki Hiwasa NS. High serum level of plasminogen activator inhibitor-1 predicts histological grade of intracerebral gliomas. *Anticancer Res.* 2008;28(1b):415–418.

67. Gerlach R, Scheuer T, Böhm M, et al. Increased levels of plasma tissue factor pathway inhibitor in patients with glioblastoma and intracerebral metastases. *Neurol. Res.* 2003;25(4):335–338.

68. Siegal T, Charbit H, Paldor I, et al. Dynamics of circulating hypoxia-mediated miRNAs and tumor response in patients with high-grade glioma treated with bevacizumab. *J. Neurosurg.* 2016;125(4):1008–1015.

69. Wang Q, Li P, Li A, et al. Plasma specific miRNAs as predictive biomarkers for diagnosis and prognosis of glioma. *J. Exp. Clin. Cancer Res.* 2012;31(1):97.

70. Morokoff A, Jones J, Nguyen H, et al. Serum microRNA is a biomarker for post-operative monitoring in glioma. *J. Neurooncol.* 2020;149(3):391–400.

71. Ivo D’Urso P, Fernando D’Urso O, Damiano Gianfreda C, et al. miR-15b and miR-21 as Circulating Biomarkers for Diagnosis of Glioma. *Curr. Genomics*. 2015;16(5):304–311.

72. Santangelo A, Imbrucè P, Gardenghi B, et al. A microRNA signature from serum exosomes of patients with glioma as complementary diagnostic biomarker. *J. Neurooncol.* 2018;136(1):51–62.

73. ParvizHamidi M, Haddad G, Ostadrahimi S, et al. Circulating miR-26a and miR-21 as biomarkers for glioblastoma multiform. *Biotechnol. Appl. Biochem.* 2019;66(2):261–265.

74. Labib EM, Ezz LR, Arab E, et al. Relevance of circulating MiRNA-21 and MiRNA-181 in prediction of glioblastoma multiforme prognosis Relevance of circulating MiRNA-21 and MiRNA-181 in prediction of glioblastoma multiforme prognosis. *Arch. Physiol. Biochem.* 2020;1–6.

75. Ilhan-Mutlu A, Wagner L, Wöhrer A, et al. Plasma microrna-21 concentration may be a useful biomarker in glioblastoma patients. *Cancer Invest.* 2012;30(8):615–621.

76. Yang K, Wang S, Cheng Y, Tian Y, Hou J. Role of miRNA-21 in the diagnosis and prediction of treatment efficacy of primary central nervous system lymphoma. *Oncol. Lett.* 2019;17(3):3475–3481.

77. Zhi F, Shao N, Wang R, et al. Identification of 9 serum microRNAs as potential noninvasive biomarkers of human astrocytoma. *Neuro. Oncol.* 2015;17(3):383–391.

78. Shi R, Wang PY, Li XY, et al. Exosomal levels of miRNA-21 from cerebrospinal fluids associated with poor prognosis and tumor recurrence of glioma patients. *Oncotarget*. 2015;6(29):26971–26981.

79. Lu Z, Tang H, Wu D, et al. Amplified voltammetric detection of miRNA from serum samples of glioma patients via combination of conducting magnetic microbeads and ferrocene-capped gold nanoparticle/streptavidin conjugates. *Biosens. Bioelectron.* 2016;86:502–507.

80. Wang J, Yi X, Tang H, et al. Direct quantification of MicroRNA at low picomolar level in sera of glioma patients using a competitive hybridization followed by amplified voltammetric detection. *Anal. Chem.* 2012;84(15):6400–6406.

81. Wang J, Lu Z, Tang H, et al. Multiplexed Electrochemical Detection of MiRNAs from Sera of Glioma Patients at Different Stages via the Novel Conjugates of Conducting Magnetic Microbeads and Diblock Oligonucleotide-Modified Gold Nanoparticles. *Anal. Chem.* 2017;89(20):10834–10840.

82. Li J, Yuan H, Xu H, Zhao H, Xiong N. Hypoxic Cancer-Secreted Exosomal miR-182-5p Promotes Glioblastoma Angiogenesis by Targeting Kruppel-like Factor 2 and 4. *Mol. Cancer Res.* 2020;18(8):1218–1231.

83. Xiao Y, Zhang L, Song Z, et al. Potential diagnostic and prognostic value of plasma circulating microrna-182 in human glioma. *Med. Sci. Monit.* 2016;22:855–862.

84. Bookland M, Gillan E, Song X, Kolmakova A. Peripheral circulation miRNA expression of pediatric brain tumors and its relation to tumor miRNA expression levels. *J. Neurosurg. Pediatr.* 2020;26(2):136–144.

85. Zhang R, Pang B, Xin T, et al. Plasma miR-221/222 Family as Novel Descriptive and Prognostic Biomarkers for Glioma. *Mol. Neurobiol.* 2016;53(3):1452–1460.

86. Faria G, Silva E, Da Fonseca C, Quirico-Santos T. Circulating cell-free DNA as a prognostic and molecular marker for patients with brain tumors under perillyl alcohol-based therapy. *Int. J. Mol. Sci.* 2018;19(6):1610.

87. Bagley SJ, Ali Nabavizadeh S, Mays JJ, et al. Clinical utility of plasma cell-free DNA in adult patients with newly diagnosed glioblastoma: A pilot prospective study. *Clin. Cancer Res.* 2020;26(2):397–407.

88. Nassiri F, Chakravarthy A, Feng S, et al. Detection and discrimination of intracranial tumors using plasma cell-free DNA methylomes. *Nat. Med.* 2020;26(7):1044–1047.

89. Chen J, Huan W, Zuo H, et al. Alu methylation serves as a biomarker for non-invasive diagnosis of glioma. *Oncotarget*. 2016;7(18):26099–26106.

90. Chen J, Gong M, Lu S, et al. Detection of serum alu element hypomethylation for the diagnosis and prognosis of glioma. *J. Mol. Neurosci.* 2013;50(2):368–375.

91. Shi W, Lv C, Qi J, et al. Prognostic value of free DNA quantification in serum and cerebrospinal fluid in glioma patients. *J. Mol. Neurosci.* 2012;46(3):470–475.

92. Mouliere F, Chandrananda D, Piskorz AM, et al. Enhanced detection of circulating tumor DNA by fragment size analysis. *Sci. Transl. Med.* 2018;10(466):4921.

93. Bang-Christensen SR, Pedersen RS, Pereira MA, et al. Capture and Detection of Circulating Glioma Cells Using the Recombinant VAR2CSA Malaria Protein. *Cells*. 2019;8(9):998.

94. Van Gool SW, Makalowski J, Bonner ER, et al. Addition of Multimodal Immunotherapy to Combination Treatment Strategies for Children with DIPG: A Single Institution Experience. *Medicines*. 2020;7(5):29.

95. Zhang W, Bao L, Yang S, et al. Tumor-selective replication herpes simplex virus-based technology significantly improves clinical detection and prognostication of viable circulating tumor cells. *Oncotarget*. 2016;7(26):39768–39783.

96. MacArthur KM, Kao GD, Chandrasekaran S, et al. Detection of brain tumor cells in the peripheral blood by a telomerase promoter-based assay. *Cancer Res.* 2014;74(8):2152–2159.

97. Gao F, Cui Y, Jiang H, et al. Circulating tumor cell is a common property of brain glioma and promotes the monitoring system. *Oncotarget*. 2016;7(44):71330–71340.

98. Müller C, Holtschmidt J, Auer M, et al. Cancer: Hematogenous dissemination of glioblastoma multiforme. *Sci. Transl. Med.* 2014;6(247):247.

99. Sullivan JP, Nahed B V., Madden MW, et al. Brain tumor cells in circulation are enriched for mesenchymal gene expression. *Cancer Discov.* 2014;4(11):1299–1309.

100. Lynch D, Powter B, Po JW, et al. Isolation of Circulating Tumor Cells from Glioblastoma Patients by Direct Immunomagnetic Targeting. *Appl. Sci.* 2020;10(9):3338.

101. Krol I, Castro-Giner F, Maurer M, et al. Detection of circulating tumour cell clusters in human glioblastoma. *Br. J. Cancer*. 2018;119(4):487–491.

102. Subeikshanan V, Dutt A, Basu D, et al. A prospective comparative clinical study of peripheral blood counts and indices in patients with primary brain tumors. *J. Postgrad. Med.* 2016;62(2):86–90.

103. Best MG, In ’t Veld SGJG, Sol N, Wurdinger T. RNA sequencing and swarm intelligence–enhanced classification algorithm development for blood-based disease diagnostics using spliced blood platelet RNA. *Nat. Protoc.* 2019;14(4):1206–1234.

104. Sol N, In ’t Veld GJG, Vancura A, et al. Tumor-Educated Platelet RNA for the Detection and (Pseudo)progression Monitoring of Glioblastoma. *Cell Reports Med.* 2020;1(7):100101.

105. Best MG, Sol N, Kooi I, et al. RNA-Seq of Tumor-Educated Platelets Enables Blood-Based Pan-Cancer, Multiclass, and Molecular Pathway Cancer Diagnostics. *Cancer Cell*. 2015;28(5):666–676.

106. Fossati G, Ricevuti G, Edwards SW, et al. Neutrophil infiltration into human gliomas. *Acta Neuropathol.* 1999;98(4):349–354.

107. Adachi-Hayama M, Adachi A, Shinozaki N, et al. Circulating anti-filamin C autoantibody as a potential serum biomarker for low-grade gliomas. *BMC Cancer*. 2014;14(1):452.

108. Kayhan A, Korkmaz TS, Baran O, et al. Preoperative systemic inflammatory markers in different brain pathologies: An analysis of 140 patients. *Turk. Neurosurg.* 2019;29(6):799–803.

109. Uegaki M, Kobayashi S, Kuramoto S, Yokoyama MM. Lymphocyte subsets in patients with brain tumors. *J. Neurooncol.* 1988;6(1):25–28.

110. Gustafson MP, Lin Y, New KC, et al. Systemic immune suppression in glioblastoma: The interplay between CD14 +HLA-DR lo/neg monocytes, tumor factors, and dexamethasone. *Neuro. Oncol.* 2010;12(7):631–644.

111. Wiencke JK, Accomando WP, Zheng S, et al. Epigenetic biomarkers of T-cells in human glioma. *Epigenetics*. 2012;7(12):1391–1402.

112. Bahador M, Gras Navarro A, Rahman MA, et al. Increased infiltration and tolerised antigen-specific CD8+ TEM cells in tumor but not peripheral blood have no impact on survival of HCMV+ glioblastoma patients. *Oncoimmunology*. 2017;6(8):e1336272.

113. M K Bhondeley 1, R D Mehra, N K Mehra, A K Mohapatra, P N Tandon, S Roy VB. Imbalances in T cell subpopulations in human gliomas in. *J. Neurosurg.* 1988;68(4):589–593.

114. Fecci PE, Mitchell DA, Whitesides JF, et al. Increased regulatory T-cell fraction amidst a diminished CD4 compartment explains cellular immune defects in patients with malignant glioma. *Cancer Res.* 2006;66(6):3294–3302.

115. Vasco C, Canazza A, Rizzo A, et al. Circulating T regulatory cells migration and phenotype in glioblastoma patients: An in vitro study. *J. Neurooncol.* 2013;115(3):353–363.

116. Mohme M, Schliffke S, Maire CL, et al. Immunophenotyping of Newly Diagnosed and Recurrent Glioblastoma Defines Distinct Immune Exhaustion Profiles in Peripheral and Tumor-infiltrating Lymphocytes. *Clin. Cancer Res.* 2018;24(17):4187–4200.

117. Goods BA, Hernandez AL, Lowther DE, et al. Functional differences between PD-1+ and PD-1- CD4+ effector T cells in healthy donors and patients with glioblastoma multiforme. *PLoS One*. 2017;12(9):e0181538.

118. Li XXX, Wang B, Gu L, et al. Tim-3 expression predicts the abnormal innate immune status and poor prognosis of glioma patients. *Clin. Chim. Acta*. 2018;476:178–184.

119. Ogden AT, Horgan D, Waziri A, et al. Defective Receptor Expression and Dendritic Cell Differentiation of Monocytes in Glioblastomas. *Neurosurgery*. 2006;59(4):902–910.

120. Pinton L, Masetto E, Vettore M, et al. The immune suppressive microenvironment of human gliomas depends on the accumulation of bone marrow-derived macrophages in the center of the lesion. *J. Immunother. Cancer*. 2019;7(1):58.

121. Guo Y, Hong W, Zhang P, et al. Abnormal polarization of macrophage-like cells in the peripheral blood of patients with glioma. *Oncol. Lett.* 2020;20(1):947–954.

122. Vidyarthi A, Agnihotri T, Khan N, et al. Predominance of M2 macrophages in gliomas leads to the suppression of local and systemic immunity. *Cancer Immunol. Immunother.* 2019;68(12):1995–2004.

123. Sippel TR, White J, Nag K, et al. Neutrophil degranulation and immunosuppression in patients with GBM: Restoration of cellular immune function by targeting arginase I. *Clin. Cancer Res.* 2011;17(22):6992–7002.

124. Wiencke JK, Koestler DC, Salas LA, et al. Immunomethylomic approach to explore the blood neutrophil lymphocyte ratio (NLR) in glioma survival. *Clin. Epigenetics*. 2017;9(1):10.

125. Gousias K, von Ruecker A, Voulgari P, Simon M. Phenotypical analysis, relation to malignancy and prognostic relevance of ICOS + T regulatory and dendritic cells in patients with gliomas. *J. Neuroimmunol.* 2013;264(1–2):84–90.

126. Pinzon-Charry A, Ho CSK, Laherty R, et al. A population of HLA-DR+ immature cells accumulates in the blood dendritic cell compartment of patients with different types of cancer. *Neoplasia*. 2005;7(12):1112–1122.

127. Raychaudhuri B, Ireland PRJ, Ko J, et al. Myeloid-derived suppressor cell accumulation and function in patients with newly diagnosed glioblastoma. *Neuro. Oncol.* 2011;13(6):591–599.

128. Gabrusiewicz K, Rodriguez B, Wei J, et al. Glioblastoma-infiltrated innate immune cells resemble M0 macrophage phenotype. *JCI Insight*. 2016;1(2):e85841.

129. Gielen PR, Schulte BM, Kers-Rebel ED, et al. Increase in Both CD14-Positive and CD15-Positive Myeloid-Derived Suppressor Cell Subpopulations in the Blood of Patients with Glioma but Predominance of CD15-Positive Myeloid-Derived Suppressor Cells in Glioma Tissue. *J. Neuropathol. Exp. Neurol.* 2015;74(5):390–400.

130. Dubinski D, Wölfer J, Hasselblatt M, et al. CD4+ T effector memory cell dysfunction is associated with the accumulation of granulocytic myeloid-derived suppressor cells in glioblastoma patients. *Neuro. Oncol.* 2016;18(6):807–818.

131. Andaloussi A El, Lesniak MS. An increase in CD4+CD25+FOXP3+ regulatory T cells in tumor-infiltrating lymphocytes of human glioblastoma multiforme. *Neuro. Oncol.* 2006;8(3):234–243.

132. Li Z, Liu X, Guo R, Wang P. CD4+Foxp3− type 1 regulatory T cells in glioblastoma multiforme suppress T cell responses through multiple pathways and are regulated by tumor-associated macrophages. *Int. J. Biochem. Cell Biol.* 2016;81(Pt A):1–9.

133. Jacobs JFM, Idema AJ, Bol KF, et al. Regulatory T cells and the PD-L1/PD-1 pathway mediate immune suppression in malignant human brain tumors. *Neuro. Oncol.* 2009;11(4):394–402.

134. Osti D, Bene M Del, Rappa G, et al. Clinical significance of extracellular vesicles in plasma from glioblastoma patients. *Clin. Cancer Res.* 2019;25(1):266–276.

135. Ricklefs FL, Maire CL, Reimer R, et al. Imaging flow cytometry facilitates multiparametric characterization of extracellular vesicles in malignant brain tumours. *J. Extracell. Vesicles*. 2019;8(1):1588555.

136. Reynés G, Vila V, Fleitas T, et al. Circulating Endothelial Cells and Procoagulant Microparticles in Patients with Glioblastoma: Prognostic Value. *PLoS One*. 2013;8(7):e69034.

137. Cumba Garcia LM, Peterson TE, Cepeda MA, Johnson AJ, Parney IF. Isolation and Analysis of Plasma-Derived Exosomes in Patients With Glioma. *Front. Oncol.* 2019;10(9):3338.

138. Muller L, Muller-Haegele S, Mitsuhashi M, et al. city and might predict survival. *Oncoimmunology*. 2015;4(6):e1008347.

139. van der Mijn JC, Sol N, Mellema W, et al. Analysis of AKT and ERK1/2 protein kinases in extracellular vesicles isolated from blood of patients with cancer. *J. Extracell. Vesicles*. 2014;3(1):25657.

140. Wang H, Jiang D, Li W, et al. Evaluation of serum extracellular vesicles as noninvasive diagnostic markers of glioma. *Theranostics*. 2019;9(18):5347–5358.

141. Shao H, Chung J, Balaj L, et al. Protein typing of circulating microvesicles allows real-time monitoring of glioblastoma therapy. *Nat. Med.* 2012;18(12):1835–1840.

142. Wang PF, Meng Z, Song HW, et al. Preoperative changes in hematological markers and predictors of glioma grade and survival. *Front. Pharmacol.* 2018;9(AUG):886.

143. Wu Y, Song Z, Sun K, et al. A novel scoring system based on peripheral blood test in predicting grade and prognosis of patients with glioma. *Onco. Targets. Ther.* 2019;12:11413–11423.

144. Huang Z, Wu L, Hou Z, et al. Eosinophils and other peripheral blood biomarkers in glioma grading: a preliminary study. *BMC Neurol.* 2019;19(1):313.

145. He ZQ, Ke C, Al-Nahari F, et al. Low preoperative prognostic nutritional index predicts poor survival in patients with newly diagnosed high-grade gliomas. *J. Neurooncol.* 2017;132(2):239–247.

146. Xu W, Wang D, Zheng · Xiaobin, et al. Sex-dependent association of preoperative hematologic markers with glioma grade and progression. *J. Neurooncol.* 2018;137:279–287.

147. He ZQ, Duan H, Ke C, et al. Evaluation of cumulative prognostic score based on pretreatment plasma fibrinogen and serum albumin levels in patients with newly diagnosed high-grade gliomas. *Oncotarget*. 2017;8(30):49605–49614.

148. Husain H, Savage W, Everett A, et al. The role of plasma GFAP as a biomarker for glioblastoma. *J. Clin. Oncol.* 2011;29(15):2095–2095.

149. Meena K. Tanwar MRG and ECH. Gene Expression Microarray Analysis Reveals YKL-40 to Be a Potential Serum Marker for Malignant Character in Human Glioma | Cancer Research. *Cancer Res.* 2002;62(15):4364–4368.

150. Peles E, Lidar Z, Simon AJ, et al. Angiogenic factors in the cerebrospinal fluid of patients with astrocytic brain tumors. *Neurosurgery*. 2004;55(3):562–567.

151. Nazari PMS, Marosi C, Moik F, et al. Low systemic levels of chemokine C-C motif ligand 3 (CCL3) are associated with a high risk of venous thromboembolism in patients with glioma. *Cancers (Basel).* 2019;11(12):2020.

152. Piccioni DE, Achrol AS, Kiedrowski LA, et al. Analysis of cell-free circulating tumor DNA in 419 patients with glioblastoma and other primary brain tumors. *CNS Oncol.* 2019;8(2):CNS34.

153. Liang R, Chen N, Li M, et al. Significance of systemic immune-inflammation index in the differential diagnosis of high- and low-grade gliomas. *Clin. Neurol. Neurosurg.* 2018;164:50–52.

154. Kemerdere R, Akgun MY, Toklu S, Alizada O, Tanriverdi T. Preoperative systemic inflammatory markers in low- and high-grade gliomas: A retrospective analysis of 171 patients. *Heliyon*. 2019;5(5):e01681.

155. Wang Z, Zhong L, Li G, et al. Pre-treatment neutrophils count as a prognostic marker to predict chemotherapeutic response and survival outcomes in glioma: A single-center analysis of 288 cases. *Am. J. Transl. Res.* 2020;12(1):90–104.

156. Wang ZL, Zhang CB, Liu YQ, Wang Z, Jiang T. Peripheral blood test provides a practical method for glioma evaluation and prognosis prediction. *CNS Neurosci. Ther.* 2019;25(8):876–883.

157. Weng W, Chen X, Gong S, Guo L, Zhang X. Preoperative neutrophil–lymphocyte ratio correlated with glioma grading and glioblastoma survival. *Neurol. Res.* 2018;40(11):917–922.

158. Wilson JRF, Saeed F, Tyagi AK, et al. Pre-operative neutrophil count and neutrophil-lymphocyte count ratio (NLCR) in predicting the histological grade of paediatric brain tumours: a preliminary study. *Acta Neurochir. (Wien).* 2018;160(4):793–800.

159. Schernberg A, Nivet A, Dhermain F, et al. Neutrophilia as a biomarker for overall survival in newly diagnosed high-grade glioma patients undergoing chemoradiation. *Clin. Transl. Radiat. Oncol.* 2018;10:47–52.

160. Weng Y, Zhang X, Han J, et al. Do Selected Blood Inflammatory Markers Combined with Radiological Features Predict Proliferation Index in Glioma Patients? *World Neurosurg.* 2018;118:e137–e146.

161. Auezova R, Ryskeldiev N, Doskaliyev A, et al. Association of preoperative levels of selected blood inflammatory markers with prognosis in gliomas. *Onco. Targets. Ther.* 2016;9:6111–6117.

162. Bao Y, Yang M, Jin C, et al. Preoperative Hematologic Inflammatory Markers as Prognostic Factors in Patients with Glioma. *World Neurosurg.* 2018;119:e710–e716.

163. Zadora P, Dabrowski W, Czarko K, et al. Preoperative neutrophil-lymphocyte count ratio helps predict the grade of glial tumor – a pilot study. *Neurol. Neurochir. Pol.* 2015;49(1):41–44.

164. Ashwath KG, Aggarwal A, Praneeth K, Singla N, Gupta K. Neutrophil-To-lymphocyte ratio: Can it be used as an adjunct tool to predict histopathological grade of brain tumor? *J. Neurosci. Rural Pract.* 2019;10(4):648–652.

165. Wang J, Xiao W, Chen W, Hu Y. Prognostic significance of preoperative neutrophil-to-lymphocyte ratio and platelet-to-lymphocyte ratio in patients with glioma. *EXCLI J.* 2019;17:505–512.

166. Liang R, Li J, Tang X, Liu Y. The prognostic role of preoperative systemic immune-inflammation index and albumin/globulin ratio in patients with newly diagnosed high-grade glioma. *Clin. Neurol. Neurosurg.* 2019;184:105397.

167. Zhao S, Cai J, Li J, et al. Bioinformatic Profiling Identifies a Glucose-Related Risk Signature for the Malignancy of Glioma and the Survival of Patients. *Mol. Neurobiol.* 2017;54(10):8203–8210.

168. Shih CC, Lee TS, Tsuang FY, et al. Pretreatment serum lactate level as a prognostic biomarker in patients undergoing supratentorial primary brain tumor resection. *Oncotarget*. 2017;8(38):63715–63723.

169. Branco M, Linhares P, Carvalho B, et al. Serum lactate levels are associated with glioma malignancy grade. *Clin. Neurol. Neurosurg.* 2019;186:105546.

170. Zhao H, Heimberger AB, Lu Z, et al. Metabolomics profiling in plasma samples from glioma patients correlataes with tumor phenotypes. *Oncotarget*. 2016;7(15):20486–20495.

171. Baumgarten P, Quick-Weller J, Gessler F, et al. Pre- and early postoperative GFAP serum levels in glioma and brain metastases. *J. Neurooncol.* 2018;139(3):541–546.

172. Aysegul Ilhan, Wolfgang Gartner, Dashurie Neziri, Thomas Czech, Wolfgang Base, Walter H Hörl LW. Angiogenic factors in plasma of brain tumour patients - PubMed. *Anticancer Res.* 2009;29(2):731–736.

173. Oto J, Plana E, Solmoirago MJ, et al. MicroRNAs and markers of neutrophil activation as predictors of early incidental post-surgical pulmonary embolism in patients with intracranial tumors. *Cancers (Basel).* 2020;12(6):1–19.

174. Baran O, Kemerdere R, Korkmaz TS, Kayhan A, Tanriverdi T. Can preoperative neutrophil to lymphocyte, lymphocyte to monocyte, or platelet to lymphocyte ratios differentiate glioblastoma from brain metastasis? *Med. (United States)*. 2019;98(50):e18306.

175. Bunevicius A, Radziunas A, Tamasauskas S, et al. Prognostic role of high sensitivity C-reactive protein and interleukin-6 in glioma and meningioma patients. *J. Neurooncol.* 2018;138(2):351–358.

176. Lee EQ, Duda DG, Muzikansky A, et al. Phase I and biomarker study of plerixafor and bevacizumab in recurrent high-grade glioma. *Clin. Cancer Res.* 2018;24(19):4643–4649.

177. Marinari E, Allard M, Gustave R, et al. Inflammation and lymphocyte infiltration are associated with shorter survival in patients with high-grade glioma. *Oncoimmunology*. 2020;9(1):1779990.

178. Vos MJ, Postma TJ, Martens F, et al. Serum Levels of S-100B Protein and Neuron-specific Enolase in Glioma Patients: A Pilot Study. *Anticancer Res.* 2004;24(4):2511–2514.

179. Holla FK, Postma TJ, Blankenstein MA, et al. Prognostic value of the S100B protein in newly diagnosed and recurrent glioma patients: a serial analysis. *J. Neurooncol.* 2016;129(3):525–532.

180. Hao Y, Li X, Chen H, et al. A Cumulative Score Based on Preoperative Neutrophil-Lymphocyte Ratio and Fibrinogen in Predicting Overall Survival of Patients with Glioblastoma Multiforme. *World Neurosurg.* 2019;128:e427–e433.

181. Pierscianek D, Ahmadipour Y, Michel A, et al. Prediction of preoperative survival in patients with glioblastoma by routine inflammatory laboratory parameters. *Anticancer Res.* 2020;40(2):1161–1166.

182. Tadej Strojnik TŠ and TTL. Prognostic Value of Erythrocyte Sedimentation Rate and C-Reactive Protein in the Blood of Patients with Glioma. *Anticancer Res.* 2014;34(1):339–347.

183. Maas SLN, Draaisma K, Snijders TJ, et al. Routine Blood Tests Do Not Predict Survival in Patients with Glioblastoma—Multivariable Analysis of 497 Patients. *World Neurosurg.* 2019;126:e1081–e1091.

184. Auezova R, Ivanova N, Akshulakov S, et al. Isocitrate dehydrogenase 1 mutation is associated with reduced levels of inflammation in glioma patients. *Cancer Manag. Res.* 2019;11:3227–3236.

185. Topkan E, Besen AA, Mertsoylu H, et al. Prognostic Value of C-Reactive Protein to Albumin Ratio in Glioblastoma Multiforme Patients Treated with Concurrent Radiotherapy and Temozolomide. *Int. J. Inflam.* 2020;2020:6947382.

186. Han S, Huang Y, Li Z, Hou H, Wu A. The prognostic role of preoperative serum albumin levels in glioblastoma patients. *BMC Cancer*. 2015;15(1):108.

187. Schwartzbaum JA, Lal P, Evanoff W, et al. Presurgical serum albumin levels predict survival time from glioblastoma multiforme. *J. Neurooncol.* 1999;43(1):35–41.

188. Borg N, Guilfoyle MR, Greenberg DC, Watts C, Thomson S. Serum albumin and survival in glioblastoma multiforme. *J. Neurooncol.* 2011;105(1):77–81.

189. Xu WZ, Li F, Xu ZK, et al. Preoperative albumin-to-globulin ratio and prognostic nutrition index predict prognosis for glioblastoma. *Onco. Targets. Ther.* 2017;10:725–733.

190. Zhou XW, Dong H, Yang Y, et al. Significance of the prognostic nutritional index in patients with glioblastoma: A retrospective study. *Clin. Neurol. Neurosurg.* 2016;151:86–91.

191. Rigamonti A, Imbesi F, Silvani A, et al. Prognostic nutritional index as a prognostic marker in glioblastoma: Data from a cohort of 282 Italian patients. *J. Neurol. Sci.* 2019;400:175–179.

192. Zhang ZY, Zhan YB, Zhang FJ, et al. Prognostic value of preoperative hematological markers combined with molecular pathology in patients with diffuse gliomas. *Aging (Albany. NY).* 2019;11(16):6252–6272.

193. Ding JD, Yao K, Wang PF, Yan CX. Clinical significance of prognostic nutritional index in patients with glioblastomas. *Med. (United States)*. 2018;97(48):e13218.

194. Wang PF, Zhang J, Cai HQ, et al. Sanbo scoring system, based on age and pre-treatment hematological markers, is a non-invasive and independent prognostic predictor for patients with primary glioblastomas: A retrospective multicenter study. *J. Cancer*. 2019;10(23):5654–5660.

195. Gousias K, Voulgaris S, Vartholomatos G, et al. Prognostic value of the preoperative immunological profile in patients with glioblastoma. *Surg. Neurol. Int.* 2014;5:89.

196. Hormigo A, Gu B, Karimi S, et al. YKL-40 and matrix metalloproteinase-9 as potential serum biomarkers for patients with high-grade gliomas. *Clin. Cancer Res.* 2006;12(19):5698–5704.

197. Bernardi D, Padoan A, Ballin A, et al. Serum YKL-40 following resection for cerebral glioblastoma. *J Neurooncol*. 2012;107(2):299–305.

198. van Linde ME, van der Mijn JC, Pham T V., et al. Evaluation of potential circulating biomarkers for prediction of response to chemoradiation in patients with glioblastoma. *J. Neurooncol.* 2016;129(2):221–230.

199. Tabouret E, Boudouresque F, Barrie M, et al. Association of matrix metalloproteinase 2 plasma level with response and survival in patients treated with bevacizumab for recurrent high-grade glioma. *Neuro. Oncol.* 2014;16(3):392–399.

200. Marfia G, Navone SE, Fanizzi C, et al. Prognostic value of preoperative von Willebrand factor plasma levels in patients with Glioblastoma. *Cancer Med.* 2016;5(8):1783–1790.

201. Pace A, Mandoj C, Antenucci A, et al. A predictive value of von Willebrand factor for early response to Bevacizumab therapy in recurrent glioma. *J. Neurooncol.* 2018;138(3):527–535.

202. Hoke M, Dieckmann K, Koppensteiner R, et al. Prognostic value of plasma d-dimer levels in patients with glioblastoma multiforme - Results from a pilot study. *Wien. Klin. Wochenschr.* 2011;123(7–8):199–203.

203. Brockmann MA, Giese A, Mueller K, et al. Preoperative thrombocytosis predicts poor survival in patients with glioblastoma. *Neuro. Oncol.* 2007;9(3):335–342.

204. Zhao H, Shen J, Hodges TR, et al. Serum microRNA profiling in patients with glioblastoma: A survival analysis. *Mol. Cancer*. 2017;16(1):59.

205. Swellam M, Ezz El Arab L, Al-Posttany AS, B. Said S. Clinical impact of circulating oncogenic MiRNA-221 and MiRNA-222 in glioblastoma multiform. *J. Neurooncol.* 2019;144(3):545–551.

206. Zhang R, Pang B, Xin T, et al. Plasma miR-221/222 Family as Novel Descriptive and Prognostic Biomarkers for Glioma. *Mol. Neurobiol.* 2016;53(3):1452–1460.

207. Li J hui, He Z qiang, Lin F hua, et al. Assessment of ctDNA in CSF may be a more rapid means of assessing surgical outcomes than plasma ctDNA in glioblastoma. *Mol. Cell. Probes*. 2019;46:101411.

208. Williams M, Zi •, Liu W, et al. Change in platelet levels during radiotherapy with concurrent and adjuvant temozolomide for the treatment of glioblastoma: a novel prognostic factor for survival. *J Cancer Res Clin Oncol*. 2012;138(10):1683–1688.

209. McNamara MG, Lwin Z, Jiang H, et al. Factors impacting survival following second surgery in patients with glioblastoma in the temozolomide treatment era, incorporating neutrophil/lymphocyte ratio and time to first progression. *J. Neurooncol.* 2014;117(1):147–152.

210. Han S, Liu Y, Li Q, et al. Pre-treatment neutrophil-to-lymphocyte ratio is associated with neutrophil and T-cell infiltration and predicts clinical outcome in patients with glioblastoma. *BMC Cancer*. 2015;15(1):617.

211. Yersal Ö, Odabaşi E, Özdemir Ö, Kemal Y. Prognostic significance of pre‑treatment neutrophil‑to‑lymphocyte ratio and platelet‑to‑lymphocyte ratio in patients with glioblastoma. *Mol. Clin. Oncol.* 2018;9(4):453.

212. Lopes M, Carvalho B, Vaz R, Linhares P. Influence of neutrophil–lymphocyte ratio in prognosis of glioblastoma multiforme. *J. Neurooncol.* 2018;136(1):173–180.

213. Gan Y, Zhou X, Niu X, et al. Neutrophil/Lymphocyte Ratio Is an Independent Prognostic Factor in Elderly Patients with High-Grade Gliomas. *World Neurosurg.* 2019;127:e261–e267.

214. Vaios EJ, Winter SF, Muzikansky A, Nahed B V, Dietrich J. Eosinophil and lymphocyte counts predict bevacizumab response and survival in recurrent glioblastoma. *Neuro-Oncology Adv.* 2020;2(1):1–11.

215. Bambury RM, Teo MY, Power DG, et al. The association of pre-treatment neutrophil to lymphocyte ratio with overall survival in patients with glioblastoma multiforme. *J. Neurooncol.* 2013;114(1):149–154.

216. Bertaut A, Truntzer C, Madkouri R, et al. Blood baseline neutrophil count predicts bevacizumab efficacy in glioblastoma. *Oncotarget*. 2016;7(43):70948–70958.

217. Boonyawan K, Hess KR, Yang J, et al. A relative increase in circulating platelets following chemoradiation predicts for poor survival of patients with glioblastoma. *Oncotarget*. 2017;8(52):90488–90495.

218. Evans SM, Putt M, Yang X-Y, et al. Initial evidence that blood-borne microvesicles are biomarkers for recurrence and survival in newly diagnosed glioblastoma patients. *J. Neurooncol.* 2016;127(2):391–400.

219. Tan Z, Shen L, Wu H, et al. Preoperative Neutrophil/Lymphocyte Ratio Is an Independent Prognostic Biomarker in Patients with Low-Grade Gliomas. *World Neurosurg.* 2019;132:e585–e590.

220. Huang Y, Ding H, Wu Q, et al. Neutrophil–lymphocyte ratio dynamics are useful for distinguishing between recurrence and pseudoprogression in high-grade gliomas. *Cancer Manag. Res.* 2019;11:6003–6009.

221. Lv Y, Zhang S, Liu Z, et al. Prognostic value of preoperative neutrophil to lymphocyte ratio is superior to systemic immune inflammation index for survival in patients with Glioblastoma. *Clin. Neurol. Neurosurg.* 2019;181:24–27.

222. Wang PF, Song HW, Cai HQ, et al. Preoperative inflammation markers and IDH mutation status predict glioblastoma patient survival. *Oncotarget*. 2017;8(30):50117–50123.

223. Alexiou G, Vartholomatos E, Voulgaris S, Zagorianakou P. Prognostic significance of neutrophil-to-lymphocyte ratio in glioblastoma. *Neuroimmunol. Neuroinflammation*. 2014;1(3):131–134.

224. Kaya V, Yildirim M, Yazici G, et al. Prognostic significance of indicators of systemic inflammatory responses in glioblastoma patients. *Asian Pacific J. Cancer Prev.* 2017;18(12):3287–3291.

225. Brenner A, Friger M, Geffen DB, Kaisman-Elbaz T, Lavrenkov K. The prognostic value of the pretreatment neutrophil/lymphocyte ratio in patients with glioblastoma multiforme brain tumors: A retrospective cohort study of patients treated with combined modality surgery, radiation therapy, and temozolomide chemotherapy. *Oncol.* 2019;97(5):255–263.

226. McGirt MJ, Chaichana KL, Gathinji M, et al. Persistent outpatient hyperglycemia is independently associated with decreased survival after primary resection of malignant brain astrocytomas. *Neurosurgery*. 2008;63(2):286–291.

227. Mayer A, Vaupel P, Struss HG, et al. Ausgeprägt negativer prognostischer Einfluss von hyperglykämischen Episoden während der adjuvanten Radiochemotherapie des Glioblastoma multiforme. *Strahlentherapie und Onkol.* 2014;190(10):933–938.

228. Derr RL, Ye X, Islas MU, et al. Association between hyperglycemia and survival in patients with newly diagnosed glioblastoma. *J. Clin. Oncol.* 2009;27(7):1082–1086.

229. Lavon I, Refael M, Zelikovitch B, Shalom E, Siegal T. Serum DNA can define tumor-specific genetic and epigenetic markers in gliomas of various grades. *Neuro. Oncol.* 2010;12(2):173–180.

230. Wang Z, Jiang W, Wang Y, et al. MGMT promoter methylation in serum and cerebrospinal fluid as a tumor-specific biomarker of glioma. *Biomed. Reports*. 2015;3(4):543–548.

231. Majchrzak-Celińska A, Paluszczak J, Kleszcz R, et al. Detection of MGMT, RASSF1A, p15INK4B, and p14ARF promoter methylation in circulating tumor-derived DNA of central nervous system cancer patients. *J. Appl. Genet.* 2013;54(3):335–344.

232. Weaver KD, Grossman SA, Herman JG. Methylated Tumor-Specific DNA as a Plasma Biomarker in Patients with Glioma. *Cancer Invest.* 2006;24(1):35–40.

233. Estival A, Sanz C, Ramirez JL, et al. Pyrosequencing versus methylation-specific PCR for assessment of MGMT methylation in tumor and blood samples of glioblastoma patients. *Sci. Rep.* 2019;9(1):11125.

234. Boisselier B, Pérez-Larraya JG, Rossetto M, et al. Detection of IDH1 mutation in the plasma of patients with glioma. *Neurology*. 2012;79(16):1693–1698.

235. Capper D, von Deimling A, Brandes AA, et al. Biomarker and histopathology evaluation of patients with recurrent glioblastoma treated with galunisertib, lomustine, or the combination of galunisertib and lomustine. *Int. J. Mol. Sci.* 2017;18(5):995.

236. Tichy J, Spechtmeyer S, Mittelbronn M, et al. Prospective evaluation of serum glial fibrillary acidic protein (GFAP) as a diagnostic marker for glioblastoma. *J. Neurooncol.* 2015;126(2):361–369.

237. Nørøxe DS, Østrup O, Yde CW, et al. Cell-free DNA in newly diagnosed patients with glioblastoma – a clinical prospective feasibility study. *Oncotarget*. 2019;10(43):4397–4406.

238. Nabavizadeh SA, Ware JB, Guiry S, et al. Imaging and histopathologic correlates of plasma cell-free DNA concentration and circulating tumor DNA in adult patients with newly diagnosed glioblastoma. *Neuro-Oncology Adv.* 2020;2(1):1–9.

239. Koch CJ, Lustig RA, Yang XY, et al. Microvesicles as a biomarker for tumor progression versus treatment effect in radiation/temozolomide-treated glioblastoma patients. *Transl. Oncol.* 2014;7(6):752–758.

240. Ebrahimkhani S, Vafaee F, Hallal S, et al. Deep sequencing of circulating exosomal microRNA allows non-invasive glioblastoma diagnosis. *npj Precis. Oncol.* 2018;2(1):1–9.

241. Manterola L, Guruceaga E, Pérez-Larraya JG, et al. A small noncoding RNA signature found in exosomes of GBM patient serum as a diagnostic tool. *Neuro. Oncol.* 2014;16(4):520–527.

242. Roth P, Wischhusen J, Happold C, et al. A specific miRNA signature in the peripheral blood of glioblastoma patients. *J. Neurochem.* 2011;118(3):449–457.

243. Ohno M, Matsuzaki J, Kawauchi J, et al. Assessment of the Diagnostic Utility of Serum MicroRNA Classification in Patients With Diffuse Glioma. *JAMA Netw. open*. 2019;2(12):e1916953.

244. Regazzo G, Terrenato I, Spagnuolo M, et al. A restricted signature of serum miRNAs distinguishes glioblastoma from lower grade gliomas. *J. Exp. Clin. Cancer Res.* 2016;35(1):124.

245. Li Z, Lu H, Yang J, et al. Analysis of the raw serum peptidomic pattern in glioma patients. *Clin. Chim. Acta*. 2013;425:221–226.

246. Zhang H, Wu G, Tu H, Huang F. Discovery of serum biomarkers in astrocytoma by SELDI-TOF MS and proteinchip technology. *J. Neurooncol.* 2007;84(3):315–323.

247. Liu J, Zheng S, Yu J kai, Zhang J min, Chen Z. Serum protein fingerprinting coupled with artificial neural network distinguishes glioma from healthy population or brain benign tumor. *J. Zhejiang Univ. Sci. B.* 2005;6(1):4–10.

248. Vaitkiene P, Urbanaviciute R, Grigas P, et al. Identification of Astrocytoma Blood Serum Protein Profile. *Cells*. 2019;9(1):16.

249. Villanueva J, Philip J, Entenberg D, et al. Serum Peptide Profiling by Magnetic Particle-Assisted, Automated Sample Processing and MALDI-TOF Mass Spectrometry. *Anal. Chem.* 2004;76(6):1560–1570.

250. Zhenjiang L, Rao M, Luo X, et al. Cytokine Networks and Survivin Peptide-Specific Cellular Immune Responses Predict Improved Survival in Patients With Glioblastoma Multiforme. *EBioMedicine*. 2018;33:49–56.

251. Baranovi Cová E, Tomáš Galanda |, Miroslav Galanda |, et al. Metabolomic profiling of blood plasma in patients with primary brain tumours: Basal plasma metabolites correlated with tumour grade and plasma biomarker analysis predicts feasibility of the successful statistical discrimination from healthy subjects-a pre. *IUBMB Life*. 2019;71(12):1994–2002.

252. Mörén L, Tommy Bergenheim A, Ghasimi S, et al. Metabolomic screening of tumor tissue and serum in glioma patients reveals diagnostic and prognostic information. *Metabolites*. 2015;5(3):502–520.

253. Björkblom B, Wibom C, Jonsson P, et al. Metabolomic screening of pre-diagnostic serum samples identifies association between α- and γ-tocopherols and glioblastoma risk. *Oncotarget*. 2016;7(24):37043–37053.

254. Shen J, Song R, Hodges TR, Heimberger AB, Zhao H. Identification of metabolites in plasma for predicting survival in glioblastoma. *Mol. Carcinog.* 2018;57(8):1078–1084.

255. Huang J, Weinstein SJ, Kitahara CM, et al. A prospective study of serum metabolites and glioma risk. *Oncotarget*. 2017;8(41):70366–70377.

256. Qu Y, Li W-C, Hellem MR, et al. MiR-182 and miR-203 induce mesenchymal to epithelial transition and self-sufficiency of growth signals via repressing SNAI2 in prostate cells. *Int. J. Cancer*. 2013;133(3):544–555.

257. Yang CH, Yue J, Pfeffer SR, et al. MicroRNA-21 promotes glioblastoma tumorigenesis by down-regulating insulin-like growth factor-binding protein-3 (IGFBP3). *J. Biol. Chem.* 2014;289(36):25079–25087.

258. Rahaman SO, Harbor PC, Chernova O, et al. Inhibition of constitutively active Stat3 suppresses proliferation and induces apoptosis in glioblastoma multiforme cells. *Oncogene*. 2002;21(55):8404–8413.

259. Schultz NA, Johansen JS. YKL-40-a protein in the field of translational medicine: A role as a biomarker in cancer patients? *Cancers (Basel).* 2010;2(3):1453–1491.

260. Chan JA, Krichevsky AM, Kosik KS. MicroRNA-21 is an antiapoptotic factor in human glioblastoma cells. *Cancer Res.* 2005;65(14):6029–6033.

261. Paugh BS, Bryan L, Paugh SW, et al. Interleukin-1 regulates the expression of sphingosine kinase 1 in glioblastoma cells. *J. Biol. Chem.* 2009;284(6):3408–3417.

262. Liu Q, Li G, Li R, et al. IL-6 promotion of glioblastoma cell invasion and angiogenesis in U251 and T98G cell lines. *J. Neurooncol.* 2010;100(2):165–176.

263. Tarassishin L, Casper D, Lee SC. Aberrant expression of interleukin-1β and inflammasome activation in human malignant gliomas. *PLoS One*. 2014;9(7):e103432.

264. Plate KH, Breier G, Weich HA, Mennel HD, Risau W. Vascular endothelial growth factor and glioma angiogenesis: Coordinate induction of VEGF receptors, distribution of VEGF protein and possible In vivo regulatory mechanisms. *Int. J. Cancer*. 1994;59(4):520–529.

265. Bresnick AR, Weber DJ, Zimmer DB. S100 proteins in cancer. *Nat. Rev. Cancer*. 2015;15(2):96–109.

266. Villeneuve J, Tremblay P, Vallières L. Tumor necrosis factor reduces brain tumor growth by enhancing macrophage recruitment and microcyst formation. *Cancer Res.* 2005;65(9):3928–3936.

267. Liu Z, Liu J, Segura MF, et al. MiR-182 overexpression in tumourigenesis of high-grade serous ovarian carcinoma. *J. Pathol.* 2012;228(2):204–215.

268. Kelimu A, Xie R, Zhang K, et al. Metabonomic signature analysis in plasma samples of glioma patients based on 1H-nuclear magnetic resonance spectroscopy. *Neurol. India*. 2016;64(2):246–251.

269. Hishii M, Nitta T, Ishida H, et al. Human glioma-derived interleukin-10 inhibits antitumor immune responses in vitro. *Neurosurgery*. 1995;37(6):1160–1167.

270. Huettner C, Paulus W, Roggendorf W. Messenger RNA expression of the immunosuppressive cytokine II-10 in human gliomas. *Am. J. Pathol.* 1995;146(2):317–322.

271. Ahmed K, Govardhan H, Roy M, et al. Cell-free circulating tumor DNA in patients with high-grade glioma as diagnostic biomarker - A guide to future directive. *Indian J. Cancer*. 2019;56(1):65–69.

272. Bettegowda C, Sausen M, Leary RJ, et al. Detection of circulating tumor DNA in early- and late-stage human malignancies. *Sci. Transl. Med.* 2014;6(224):24.

273. Balkwill F. Tumour necrosis factor and cancer. *Nat. Rev. Cancer*. 2009;9(5):361–371.

274. Xia C, Braunstein Z, Toomey AC, Zhong J, Rao X. S100 proteins as an important regulator of macrophage inflammation. *Front. Immunol.* 2018;8(Jan):1.
